# Triazole-Based Radioligands for PET of P2X_7_R: Syntheses, Conformational Studies, and Preliminary Autoradiographic Evaluation of **[^18^F]AM-10**

**DOI:** 10.1021/acsomega.5c04531

**Published:** 2025-08-06

**Authors:** Anna Marešová, Michal Jurášek, Ivan Raich, Bohumil Dolenský, Vladimir Shalgunov, Matthias Manfred Herth, Petr Džubák, Libor Procházka, Hana Vinšová, Daniel Seifert, Ondřej Lebeda, Pavel Drašar, Paul Cumming, Alexander Popkov

**Affiliations:** † Department of Chemistry of Natural Compounds, 52735 University of Chemistry and Technology, Technická 5, 160 00 Prague, Czech Republic; ‡ Department of Analytical Chemistry, 52735 University of Chemistry and Technology, Technická 5, 160 00 Prague, Czech Republic; § Department of Drug Design and Pharmacology, University of Copenhagen, Jagtvej 162, 2100 Copenhagen, Denmark; ∥ Faculty of Medicine and Dentistry, Palacký University and University Hospital in Olomouc, Institute of Molecular and Translational Medicine (IMTM), Hněvotínská 1333/5, 779 00 Olomouc, Czech Republic; ⊥ Laboratory of Experimental Medicine, IMTM, University Hospital Olomouc, Hněvotínská 976/3, 779 00 Olomouc, Czech Republic; # Department of Radiopharmaceuticals, Nuclear Physics Institute of the Czech Academy of Sciences, Husinec - Řež 130, 250 68 Řež, Czech Republic; ∇ Department of Nuclear Medicine, 27252 University Hospital Bern, Freiburgstrasse 18, 3010 Bern, Switzerland; ⬡ School of Psychology and Counselling, Queensland University of Technology, Kelvin Grove QLD 4059, Australia; ▼ Institute of Organic Chemistry, Johannes Kepler University Linz, Altenberger Straße 69, 4040 Linz, Austria; □ Samo Biomedical Centre, Na Klinku 1082, 530 06 Pardubice, Czech Republic

## Abstract

The P2X_7_ receptor is an emerging target for molecular imaging of inflammation in the brain and peripheral tissues. In this work, we focus on five triazole-based ligands with high affinity and selectivity for P2X_7_ receptors (**JNJ-64413739**, **JNJ-55308942**, **AM-10**, **AM-12**, and **AM-15**), which are amenable to autoradiography and positron emission tomography (PET) imaging. We studied the phenomenon of conformational and rotational changes of these molecules by NMR and *ab initio* calculations. The reaction of ligands **AM-10** and **AM-12** with [^18^F]­fluoride resulted in an isotopic exchange on the pyrimidine ring, leaving the halogen atoms on the acyl moieties intact. The reaction yielded **[** ^**18**^ **F]­AM-10** with a radiochemical yield as high as 27% and a molar activity as high as 152 GBq/μmol. Quantitative autoradiography with **[** ^**18**^ **F]­AM-10** in sagittal mouse brain cryostat sections indicated a maximum specific binding (*B*_max_) of 15.8 ± 2.8 pmol/g of wet weight and a dissociation constant (*K*_D_) of 16.6 ± 5.1 nM. Thus, we present the first synthesis of **[** ^**18**^ **F]­AM-10** by isotopic exchange and confirm its specific binding at mouse brain P2X_7_ receptors, which should warrant its use in animal and human PET investigations.

## Graphical abstract

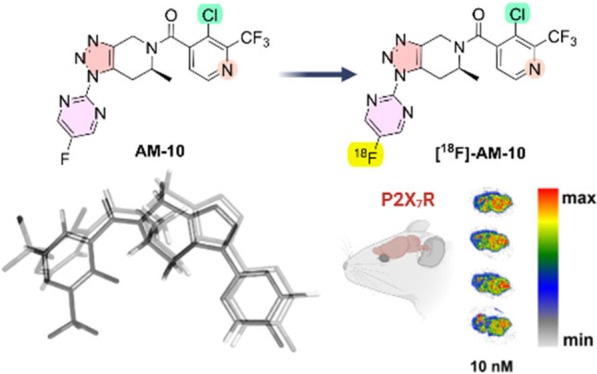

## Introduction

P2X_7_ is a ligand-gated ion channel, which has widespread expression in the brain, immunocompetent cells of the central and peripheral nervous system, and in peripheral

tissues.^1,2^ Activation of P2X_7_ receptors by the endogenous ligand ATP can mediate host immune responses against exogenous pathogens or endogenous factors,^3,4^ with important involvement in tumor biology.^5,6^ Due to its roles in diverse aspects of human physiology and pathology, the P2X_7_ receptor is a rapidly emerging target for the development of pharmaceuticals^7,8^ and radioligands for molecular imaging by positron emission tomography (PET)^9–11^ (Figure 1A).

**Figure 1:**
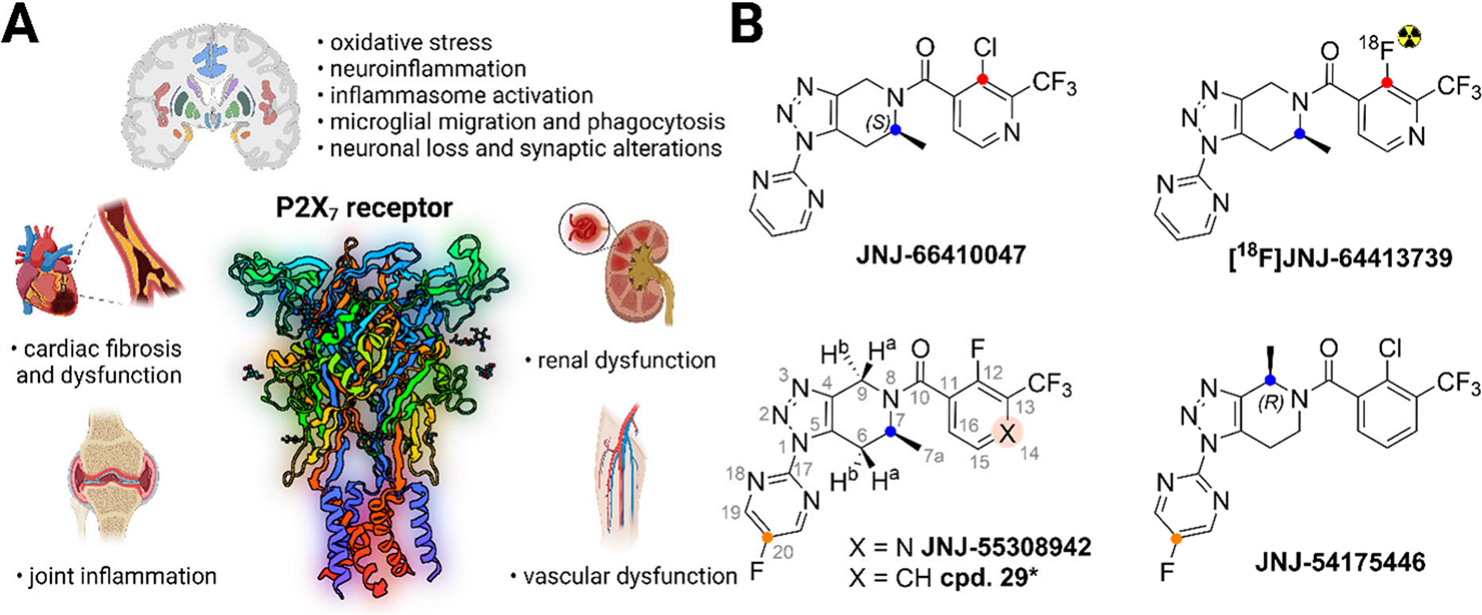
Importance of the P2X_7_ receptor in pathology and disease (A) and molecular structures of triazole-based P2X_7_ ligands (B). Created with Biorender.com.

Tricyclic triazole derivatives show high affinity *in vitro* for the P2X_7_ receptor.^7^ The main pharmacophore is considered to be a rigid triazolo-piperidine arrangement. One of the most affine and selective ligands for P2X_7_ yet described is the antagonist ligand **JNJ-55308942** (Figure 1B), which binds with high affinity at recombinant P2X_7_ receptors from human (IC_50_ 4.8 ± 1.1 nM) and rat (IC_50_ 5.9 ± 1.0 nM), while showing little affinity for cytochrome P450 enzymes (IC_50_ > 10 μM). Compound **29** (Figure 1B) is a structurally similar tricyclic ligand possessing higher lipophilicity, which might be a beneficial property for brain PET imaging studies. Furthermore, Chrovian et al.^8^ showed good affinity for **29** at human (IC_50_ 10.0 ± 3.7 nM) and rat P2X_7_ receptors (IC_50_ 15.0 ± 7.4 nM) *in vitro*. Given these results, we hypothesized that **29** and **[** ^**18**^ **F]­JNJ-55308942** might both serve admirably in brain PET studies. Although there has been no report on the radiosynthesis and binding properties of **[** ^**18**^ **F]­JNJ-55308942**, its structural congener **[** ^**18**^ **F]­JNJ-64413739** (Figure 1B) showed high-affinity binding at human (IC_50_ 1.0 ± 0.2 nM) and rat (IC_50_ 2.0 ± 0.6 nM) P2X_7_ receptors.^12^ Furthermore, PET investigations in nonhuman primates with **[** ^**18**^ **F]­JNJ-64413739** showed occupancy *in vivo* by the P2X_7_ antagonist **JNJ-54175446** (Figure 1B),^12^ and reversible binding in the brains of healthy volunteers, with quantitation relative to an image-derived arterial input function.^13^ Other work established the dosimetry and test-retest reproducibility **[** ^**18**^ **F]­JNJ-64413739** for PET quantification of P2X_7_ receptors in the human brain.^14^ Whereas the original SAR publication stated that the fluorine substituent in the pyrazine fragment ensured higher specificity for the P2X_7_ receptor, it has been uncertain why fluorine was replaced with hydrogen during the development of the PET radiopharmaceutical **[** ^**18**^ **F]­JNJ-64413739**. While earlier radiosynthesis employed aromatic nucleophilic substitution of the chlorine atom with [^18^F]­fluorine, we hypothesized that the fluorine-19 substituent in the pyrazine fragment of compounds **AM**-**10** and **AM**-**12** would also be amenable to ^18^F-labeling by an isotopic exchange mechanism.

In this work, we describe the preparation of three ^18^F-radiolabeled ligands of P2X_7_ receptors, namely, the structurally similar PET radiopharmaceutical candidates **AM**-**10** and **AM-12** (Scheme 1B), which differ with respect to their lipophilicity, and the known PET radiopharmaceutical **[** ^**18**^ **F]­JNJ-64413739** (Figure 1B, Scheme 1A), which may represent the current gold standard for P2X_7_ receptor PET investigations. For the precursors **AM**-**10** and **AM**-**12** (Scheme 1C), we tested the hypothesis that [^18^F]fluorine isotopic exchange in the pyrazine fragment of the precursor would lead to the labeled products **[** ^**18**^ **F]­AM-10** and **[** ^**18**^ **F]­AM-12**, rather than ^18^F-labeling of the acyl residues by the nucleophilic substitution of the respective halogen atoms (Scheme 1C). We also undertook a preliminary quantitative autoradiographic study of **[** ^**18**^ **F]­AM-10** binding in mouse brain cryostat sections. Since there is poor understanding of the rotational and conformational behavior of triazole-based ligands, we undertook a detailed NMR study of these properties of **JNJ-55308942**, **AM-10**, **AM-12**, and **AM-15**, with confirmation of experimental observations by *ab initio* calculations.

**Scheme 1:**
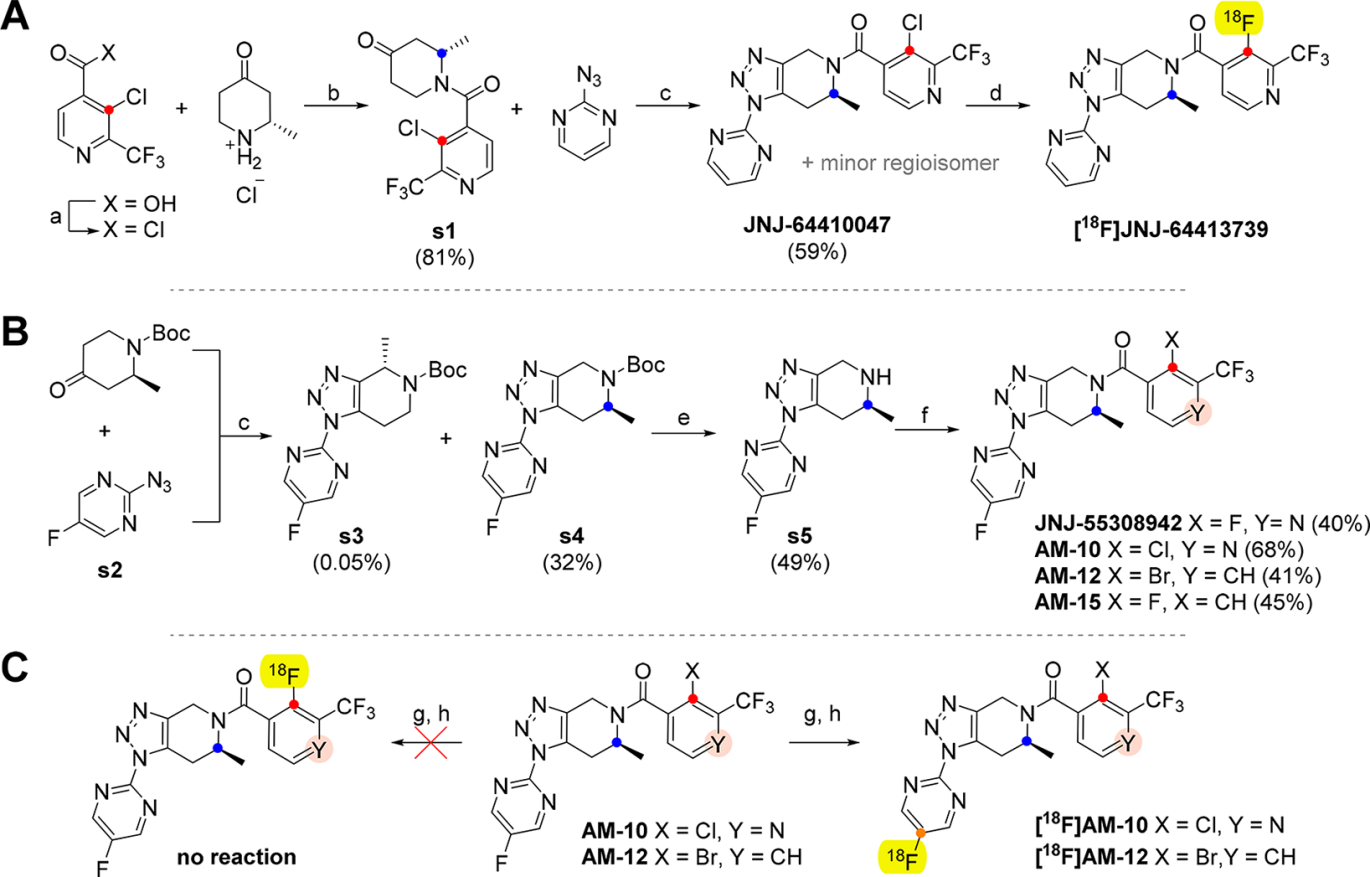
Synthesis of Triazole-Based P2X_7_ Receptor Ligand Precursors and Radioligands^a^

a A – Reproduction of the synthesis of **[^18^F]­JNJ-64413739** according to Kolb et al., 2019.; B – Synthesis of analogues of **JNJ-55308942**; C – Radiolabelling of **AM-10** and **AM-12**. *Reagents and conditions*: (a) (COCl)_2_, DCM, DMF (cat.), 17 h, RT; (b) TEA, DCM, 3 h, RT; (c) *i*. toluene, pyrrolidine, *t* = 4 h for JNJ-64410047 and *t* = 20 min for **s3** and **s4**, 110 °C; *ii*. 0 °C, DCM, NaHCO_3_, *m*-CPBA, NaOH (aq.), 30 min, RT; (d) K_222_/K oxalate and carbonate/[^18^F]­F^–^, DMSO, 0 min, 135 °C; (e) *i*. HCl in 1,4-dioxane, DCM, RT, 4 h; *ii*. MeOH, RT, 3 days; *iii*. NaHCO_3_, DCM, RT, 1 h; (f) *i.* (hetero)­aromatic acyl chlorides were prepared by procedure (a); *ii.* TEA, DCM, RT, 1 h; (g) Bu_4_NOMs/K^+^/[^18^F]­F^–^, DMSO, 135 °C, 5 min or K_222_/K^+^/[^18^F]­F^–^, DMSO, 135 °C, 5 min; (h) Bu_4_NOMs/K^+^/[^18^F]­F^–^, DMSO, 135 °C, 5 min.

## Results and Discussion

### Chemistry and Radiochemistry

For the synthesis of **[** ^**18**^ **F]­JNJ-64413739** (Scheme 1A), we preformed the synthesis of the precursor **JNJ-64410047** according to the procedure described by Chrovian et al.^8^ Kolb et al.^12^ lacked sufficient detail to enable reproduction of the synthesis. However, there is a brief analytical description and report of chemical yields in the Supplemental data of Kolb et al.^12^ In the reaction, the acylation of (*S*)-2-methyl-4-oxopiperidine with 3-chloro-2-(trifluoromethyl)­isonicotinoyl chloride provided a ketone (**s1**), which was then transformed by a 1,3-dipolar cycloaddition/Cope elimination to give the desired chromatographic standard **JNJ-55308942** in 59% yield (ref 8 40%). The cycloaddition yielded two regioisomers but always favored the C-7 methylation (see Figure 1B for numbering). NMR analysis of our **JNJ-64410047** product clearly showed the presence of rotamers, although HPLC analysis did not confirm the presence of the second expected regioisomer. Thus, we suppose that the second regioisomer was present in a negligible proportion. The radiotracer **[** ^**18**^ **F]­JNJ-64413739** was obtained after due optimization of the reaction conditions (Table 1), with 2–6% decay-corrected radiochemical yield (RCY; Table 1), which is (in the best-case scenario) superior to the published report of 3.1 ± 2.0% RCY.^12^ The radiochemical purity (RCP) was >99%, and the total synthesis time was 70 min.

**Table 1:** Synthesis of [^18^F]­F-Labeled **JNJ-64413739**

				labeling				radiochemical yield
entry	**initial activity** **^18^F [**GBq]	**QMA for trapping ^18^F (form, weight)**	eluent/catalyst	solvent	precursor (mg)	temperature (°C)	time (min)	(%, d.c.)
**1**	24	K_2_CO_3_,130 mg	8.3 mg Bu_4_NHCO_3_	0.8 mL DMSO	1.8	130	20	none
**2**	31			0.9 mL DMF	0.9	100	10	none
**3**	15		15 mg K_222_ + 4.8 mg KHCO_3_		0.9	100	10	none
**4**	5		8.3 mg Bu_4_NHCO_3_	0.9 mL DMSO	2.0	100	10	none
**5**	4	C_2_K_2_O_4_,130 mg	15 mg K_222_ + 5.8 mg C_2_K_2_O_4_ + 0.04 mg K_2_CO_3_		2.8	135	10	5.6
**6**	11	C_2_K_2_O_4_,46 mg	10.5 mg 18-Crown-6 + 6.4 mg C_2_K_2_O_4_ + 0.04 mg K_2_CO_3_	0.6 mL DMSO	0.6	135	10	none
**7**	11		15 mg K_222_ + 5.8 mg C_2_K_2_O_4_ + 0.04 mg K_2_CO_3_	0.6 mL DMA	1.8	135	10	0.1
**8**	11	C_2_K_2_O_4_,60 mg		0.6 mL DMSO	1.0	135	10	6.1
**9**	44				1.4	135	10	2.1
**10**	15			0.6 mL DMI	2.1	135	10	none
**11**	33	C_2_K_2_O_4_,130 mg		0.6 mL 2,3-dimethyl-2-butanol	1.3	135	10	none
**12**	14		15 mg K_222_ + 9.6 mg KH_2_PO_4_ + 0.04 mg K_2_CO_3_	0.6 mL DMSO	1.6	135	10	0.0

The approach of Chrovian et al.^8^ inspired our syntheses of the ligands **JNJ-55308942**, **AM-10**, **AM-12**, and **AM-15** (Scheme 1B). However, our approach differed from that for the preparation of **JNJ-64410047** with respect to our use of a Boc-protected ketone and 2-azido-5-fluoropyrimidine (**s2**) for the cycloaddition/Cope elimination reaction. The yield of mixed triazole products **s3** and **s4** was 47%, compared to the 65% reported by Chrovian et al.^8^ Kolb et al.^12^ introduced supercritical fluid chromatography (SFC) purification for regioisomer separation, whereas we used silica gel column chromatography (CHCl_3_-MeOH 100:1, *v*/*v*) for partial separation of the regioisomers by fractionation of the eluent (see Supporting Information 2. Synthetic Procedures). After the first separation, we obtained pure **s4** in 24% yield and another fraction containing both isomers. Chromatography of the impure fraction gave an additional 8% yield of **s4** and traces of pure **s3** (0.05%). NMR spectra of the separated regioisomers are shown in Supporting Information Figure S1. We performed Boc cleavage by treating **s4** with HCl to give the secondary amine **s5** with 49% yield (ref 53%).^8^ The final reactions were *N*-acylations of **s5** by appropriate substituted (hetero)­aromatic carboxylic acid chlorides, closely following the procedure described above for **JNJ-64410047** production. The yields of acylation giving **AM-10**, **AM-12**, and **AM-15** were generally lower than the 87% for **JNJ-55308942** (for structure, see Figure 1B).

Surprisingly, during radiolabeling of **AM-10** nor **AM-12** with fluorine-18, we observed no aromatic nucleophilic substitution of chlorine or bromine at the carbon-12 with [^18^F]fluorine (Scheme 1C, Table 2). Therefore, we hypothesized that the reaction proceeded by fluorine-18 isotopic exchange in the pyrazine fragment of the precursor at C-20 (for numbering used, see Figure 1B). This model may explain the Szardening group’s decision to develop **[** ^**18**^ **F]­JNJ-64413739**, which omits the fluorine-19 atom in the pyrazine fragment that is present in **JNJ-55308942**. In their reports,^12,14^ the authors have not explicitly expressed any rationale for their decision to prepare an analogue without the fluorine atom in the pyrazine fragment. In the case of **[** ^**18**^ **F]­AM-10**, the intact chlorine atom secured higher affinity toward P2X_7_ receptors compared to the analogue where the chlorine is substituted with fluorine (**JNJ-55308942**).^8^

**Table 2:** Radio-TLC Yields **[** ^**18**^ **F]­AM-10** and **[** ^**18**^ **F]­AM-12** According to the Temperature and Time of the Reaction^a^

entry	compd. AM-10/12 [mg]	precondition **of QMA** ^b^	elution of QMA^b^	*T* [°C]	time [min]	RCC^c^ [%]	RCY^d^ [%]
**1**	**10** 2.8 mg	C_2_K_2_O_4_	K_222_ (40 μmol), C_2_K_2_O_4_ (35 μmol), K_2_CO_3_ (0.3 μmol), 50% MeCN	135	5	61	N/A
**2**	**10** 2.8 mg	C_2_K_2_O_4_	K_222_ (40 μmol), C_2_K_2_O_4_ (35 μmol), K_2_CO_3_ (0.3 μmol), 50% MeCN	135	10	52	N/A
**3**	**10** 2.8 mg	C_2_K_2_O_4_	K_222_ (40 μmol), C_2_K_2_O_4_ (35 μmol), K_2_CO_3_ (0.3 μmol), 50% MeCN	135	10	N/A	6
**4**	**12** 2.8 mg	K_2_CO_3_	Bu_4_NOMs (20 μmol), 50% MeCN	155	5	71	N/A
**5**	**12** 2.8 mg	K_2_CO_3_	Bu_4_NOMs (20 μmol), 50% MeCN	155	10	70	N/A
**6**	**10** 2.8 mg	K_2_CO_3_	Bu_4_NOMs (20 μmol), 50% MeCN	155	5	59	N/A
**7**	**10** 2.8 mg	K_2_CO_3_	Bu_4_NOMs (20 μmol), 50% MeCN	155	10	55	N/A
**8**	**10** 2.0 mg	K_2_CO_3_	Bu_4_NOMs (20 μmol), 50% MeCN	135	5	73	N/A
**9**	**10** 2.0 mg	K_2_CO_3_	Bu_4_NOMs (20 μmol), 50% MeCN	135	10	65	N/A
**10**	**10** 1.0 mg	K_2_CO_3_	Bu_4_NOMs (20 μmol), 50% MeCN	135	5	56	N/A
**11**	**10** 1.0 mg	K_2_CO_3_	Bu_4_NOMs (20 μmol), 50% MeCN	135	10	45	N/A
**12**	**10** 0.5 mg	K_2_CO_3_	Bu_4_NOMs (20 μmol), 50% MeCN	135	5	36	N/A
**13**	**10** 0.5 mg	K_2_CO_3_	Bu_4_NOMs (20 μmol), 50% MeCN	135	10	35	N/A
**14**	**10** 0.1 mg	K_2_CO_3_	Bu_4_NOMs (20 μmol), 50% MeCN	135	5	8	N/A
**15**	**10** 0.1 mg	K_2_CO_3_	Bu_4_NOMs (20 μmol), 50% MeCN	135	10	6	N/A
**16**	**10** 0.2 mg	K_2_CO_3_	Bu_4_NOMs (20 μmol), 50% MeCN	135	5	N/A	2.5
**17**	**10** 0.2 mg	K_2_CO_3_	Bu_4_NOMs (20 μmol), 50% MeCN	135	5	N/A	4.7

a The QMAs were preconditioned with C_2_K_2_O_4_ or K_2_CO_3_ (0.5 M, 10 mL).

b Anion-exchange cartridge.

c Radiochemical conversion.

d Radiochemical yield.

The literature procedure for the compound **JNJ-64410047** with chlorine substitution by fluorine-18 (ref 8) inspired our first experiments toward labeling of **AM**-**10**. We thus prepared **[** ^**18**^ **F]­AM-10** by reaction of the corresponding chloro-precursor **AM-10** with [^18^F] fluoride in the presence of potassium carbonate and Kryptofix 222 (K_222_). The radiochemical conversion (RCC) was 61% after five min, and repetition under the same conditions gave a 6% RCY, which is superior to the previously published value of 3.1 ± 2.0 for ^18^F-JNJ-64413739 (ref 12). To increase the molar activity, we applied a modified procedure, which secured higher RCYs, while using a lower amount of the precursor.^15^ The corresponding chloro-precursor **AM**-**10** or bromo-precursor **AM**-**12** reacted with [^18^F]­fluoride in the presence of Bu_4_NOMs (Table 2). Furthermore, in comparing the yields of the reaction at 135 and 155 °C, we found a better RCC at the lower temperature. When starting with 8.28 GBq of ^18^F-fluoride, the lowest tested amount of precursor **AM**-**10** (0.2 mg) gave the highest molar activity (9.3 GBq/μmol), which is sufficient for autoradiography *in vitro* and small animal PET experiments and also meets the general requirements for human PET applications^16^ (Table 2).

After due optimization, our most successful radiosynthesis with this precursor gave a molar activity of 152 GBq/μmol when starting with 378 GBq of ^18^F-fluoride (Table 3). In the next step of the radiofluorination optimization, we discovered that Bu_4_NOMs is prone to decomposition during prolonged storage, and that using partially decomposed Bu_4_NOMs in the reaction reduces the RCY. Our original procedure was to prepare a stock solution of Bu_4_NOMs in methanol and store the solution in the dark at 4 °C for up to six months. The original bottle with the solid chemical had been similarly stored for up to six years. Entries 1–12 in Table 3 correspond to the usage of the stock solution of Bu_4_NOMs. When using a new bottle of Bu_4_NOMs for preparation in a fresh solution in methanol (entries 13–15 in the Table 3), the optimal reaction temperature proved to be 120 °C rather than 135 °C, and the reaction time 5 min instead of 8 min. Using the fresh solution of Bu_4_NOMs in methanol at 135 °C led to only 2.1% RCY. Here, we used a smaller 32 mg QMA cartridge equilibrated with potassium phosphate solution instead of the 64 mg QMA cartridge. The milder reaction conditions led to greater survival of **AM-10** during the fluorine isotopic exchange reaction, leading to lower molar activity of the product (Table 3, entries 13 and 14). At the same time, the decay-corrected RCYs were much higher, at 24.2 and 26.9%. To obtain higher molar activity, we halved the amount of the precursor (0.1 mg instead of the previously used 0.2 mg; Table 3, entry 15). Indeed, this procedure gave higher molar activity (18 GBq/μmol compared to 14 GBq/μmol for the entry 14), but RCY dropped to 7.5%. Further optimization of the reaction conditions is underway.

**Table 3:** Synthesis of [^18^F]­F-Labeled **AM-10**

				labeling							
entry	**initial activity ^18^F[GBq]**	**QMA for trapping ^18^F (form, weight)**	eluent/catalyst	**DMSO** [mL]	**AM-10** [μg]	**temperature** [°C]	**time** [min]	separation of AM-10	**total time of synthesis [min]**	**radiochemical yield** [%, d.c.]	**specific activity** [GBq/μmol]
**1**	24	K_2_CO_3_, 130 mg	1 mL 20 mM-Bu_4_NOMs	0.9	200	135	5	HPLC	63	1.1	24.4
**2**	9	C_2_K_2_O_4_, 130 mg	15 mg K_222_ + 5.8 mg C_2_K__2__O_4_ + 0.04 mg K_2_CO_3_ in 0.7 mL MeCN/H_2_O 1:1	0.9	200	135	5	HPLC	67	1.6	n. d.
**3**	12	K_2_CO_3_, 130 mg	1 mL 20 mM-Bu_4_NOMs	0.9	200	135	5	SPE	47	7.9	16.2
**4**	83	K_2_CO_3_, 130 mg	1 mL 20 mM-Bu_4_NOMs	0.9	200	135	5	SPE	38	3.1	n. d.
**5**	63	K_3_PO_4_, 60 mg	1 mL 20 mM-Bu_4_NOMs	0.6	200	135	10	HPLC	73	7.3	43.7
**6**	35	K_2_HPO_4_, 60 mg	1 mL 20 mM-Bu_4_NOMs	0.6	280	135	10	HPLC	79	1.6	53.7
**7**	63	K_3_PO_4_, 60 mg	1 mL 20 mM-Bu_4_NOMs	0.6	200	135	10	HPLC	79	1.8	43.7
**8**	143	K_3_PO_4_, 60 mg	1 mL 20 mM-Bu_4_NOMs+0.1 mL *tert*-amyl alcohol	0.6	200	135	5	HPLC	65	1.7	12.4
**9**	378	K_3_PO_4_, 60 mg	1 mL 20 mM-Bu_4_NOMs+0.1 mL *tert*-amyl alcohol	0.6	200	135	8	HPLC	72	1.8	152.1
**10**	395	K_3_PO_4_, 60 mg	1 mL 20 mM-Bu_4_NOMs+0.1 mL *tert*-amyl alcohol	0.6	200	135	8	HPLC	71	2.0	81.8
**11**	350	K_3_PO_4_, 60 mg	1 mL 20 mM-Bu_4_NOMs	0.6	200	135	8	HPLC	68	2.5	121.7
**12**	307	K_3_PO_4_, 60 mg	1 mL 20 mM-Bu_4_NOMs	0.6	200	135	8	HPLC	65	2.3	116.7
**13**	1.3	K_3_PO_4_, 32 mg	1 mL 20 mM-Bu_4_NOMs	0.6	200	120	5	HPLC	65	24.2	1.7
**14**	10	K_3_PO_4_, 32 mg	1 mL 20 mM-Bu_4_NOMs	0.6	200	120	5	HPLC	66	**26.9**	13.5
**15**	7	K_3_PO_4_, 32 mg	1 mL 20 mM-Bu_4_NOMs	0.6	100	120	5	HPLC	62	7.5	17.9

### In Vitro Autoradiography

We tested our new tracer **[** ^**18**^ **F]­AM-10** in the setting of autoradiography *in vitro*. Displacement studies in 20-μm cryostat sections prepared from the frozen mouse brain indicated widespread specific binding (Figure 2). In saturation binding studies (*n* = 4), we obtained maximal specific binding (*B*_max_) for entire coronal sections of 15.8 ± 2.8 pmol/g and an apparent dissociation constant (*K*_D_) of 16.6 ± 5.1 nM. We present a comparison of these results with literature reports for other P2X_7_ ligands in the literature (Table S1). This binding affinity of **[** ^**18**^ **F]­AM-10** measured in mouse brain cryostat sections falls within the range of reports for other P2X_7_ radioligands in various preparations, which extended from 1 nM [^11^C]­GSK1482160 (ref 17) to 25 nM [^18^F]­FTTM (ref 18). The *B*_max_ in cryostat sections was at least five-fold lower than earlier reports for P2X_7_ ligands in brain membrane preparations (see Table S1), perhaps reflecting binding site compartmentation or the effects of a chaperone lost during membrane processing.^19^

**Figure 2:**
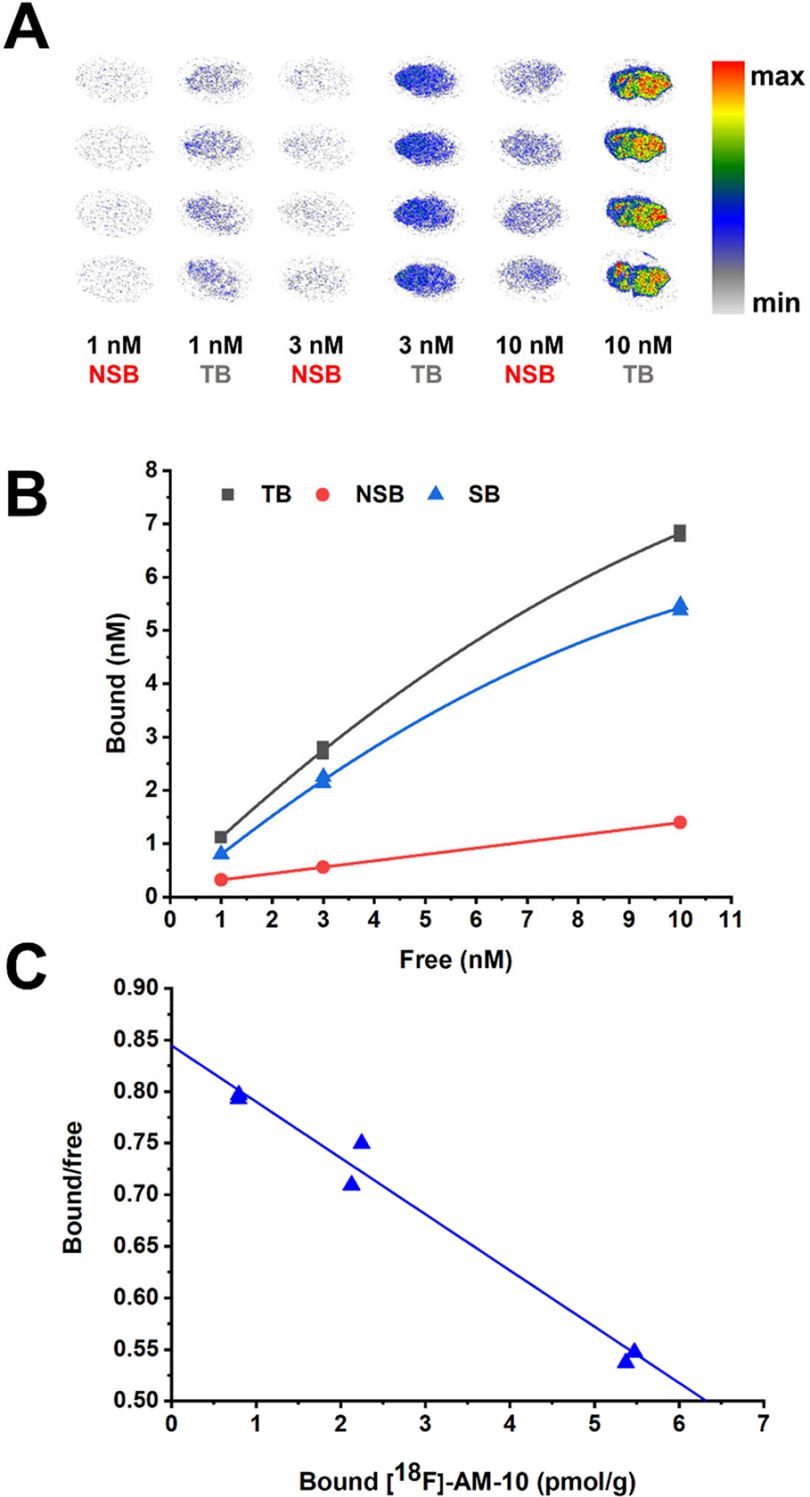
(A) *In vitro* autoradiography of **[** ^**18**^ **F]­AM-10** in mouse brain sagittal cryostat sections (20 μm-thick). (B) Representative binding curves for the P2X_7_ ligand **[** ^**18**^ **F]­AM-10**. Total binding (TB) and nonspecific binding (NSB) are shown for three different radioligand concentrations (1, 3, and 10 nM) after 60 min incubation at room temperature. Nonspecific binding was defined with the addition of 10 μM GSK1482160. Specific binding (SB) was calculated by subtracting nonspecific from total binding. (C) Scatchard plot of the saturation binding data.

### NMR and Computational Study

The ^1^H and ^19^F NMR spectra of the compounds **JNJ-55308942** and **AM-10**, **AM-12**, and **AM-15** at variable temperatures revealed that these compounds exist as a mixture of four rather stable conformers, which interconvert at higher temperature (Figure S2-6 and Table S1). Since a molecule’s conformation affects its pharmacological properties,^20^ we explored that phenomenon more deeply by QM calculation studies of **JNJ-55308942**. In accord with the spectral observations, we identified four energetically meaningful conformers (Figure 3), with populations as shown in Table 4. The conformers have almost identical geometry on the piperidine ring but differ regarding the configuration of their *N*-substituent. The most stable conformers **Aa** and **Ab** carry the carbonyl group close to proton H9b (dihedral angle C9–N8–C10–C11, ExTor2 around 0°), while conformers **Ba** and **Bb** have the carbonyl close to proton H7 (ExTor2 around 180°). Proton H16 lies above the piperazine ring (i.e., on the same side as the methyl group) for conformers **Aa** and **Ba**, whereas H16 lies under the ring for conformers **Ab** and **Bb.**

**Figure 3:**
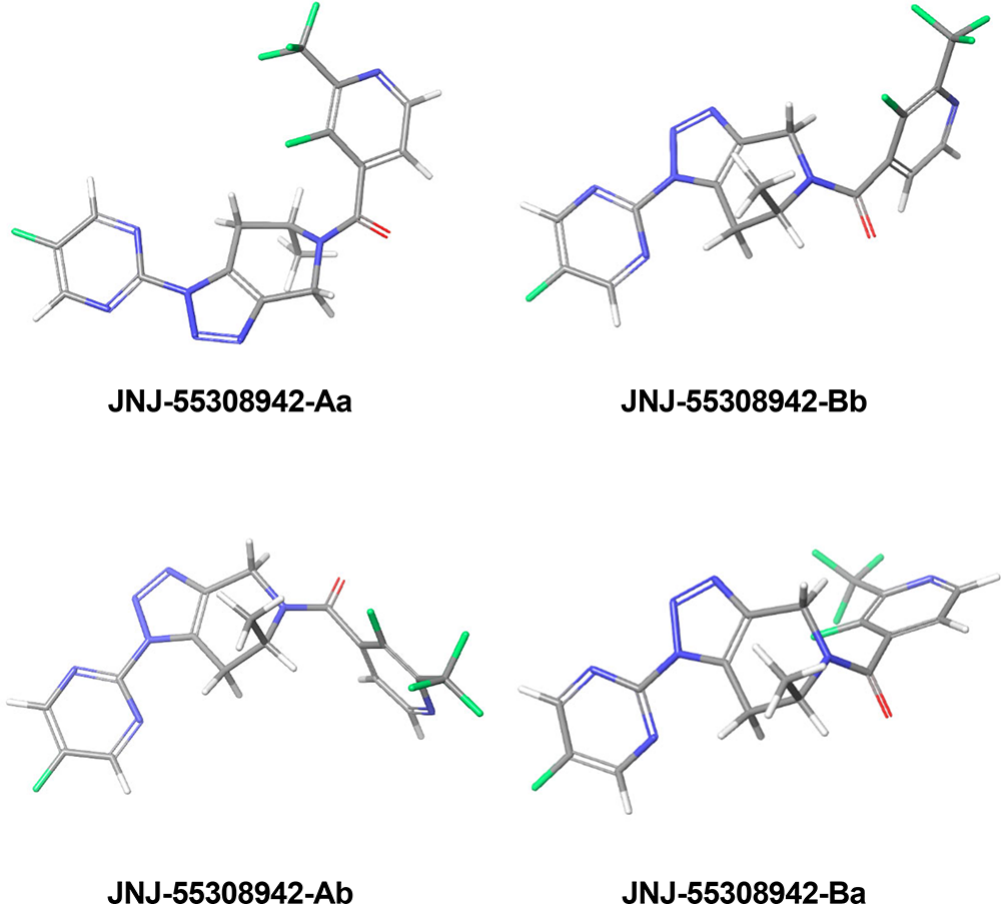
Populated conformers of **JNJ-55308942** found by QM calculation.

**Table 4:** Calculated Populations and Torsion Angles for **JNJ-55308942** (See Figure 3)

conformer	population [%]	N2–N1–C17–N18 [°]	C7–N8–C10–C11 [°]	N8–C10–C11–C16 [°]
**JNJ-55308942-Aa**	47	–19.1	13.4	–124.2
**JNJ-55308942-Bb**	35	–22.8	–176.6	–129.1
JNJ-55308942-Ab	9	–21.2	–1.7	113.2
**JNJ-55308942-Ba**	9	160.1	166.6	118.4

Overall, the DFT computational results agreed very well with the NMR data for **JNJ-55308942**. The additive computed populations of conformers **Aa** and **Ab** (56%) and **Ba** and **Bb** (44%) were close in magnitude to the experimentally observed values (see Table S2). Conformers **Aa** and **Ab** have torsion angles of 13.7° at C7–N8 and – 1.7° at C10–C11 (i.e., close to 0°), while conformers **Ba** and **Bb** have C10–C11 torsion angles close to 180° (166.6 and – 176.6°, respectively).

NMR gave good identification of each of the four conformers for the compounds **AM-10** and **AM-12**, in which the energy barrier of the rotation around the C10–C11 bond was significantly higher due to the bulkiness of chlorine or bromine in the *ortho* position. In the cases of compounds **JNJ-55308942** and **AM-15** (fluorine in the *ortho* position), the rotation barrier was significantly lower such that most of the NMR signals of conformers **Aa** and **Ab** (as well as **Ba** and **Bb**) were around coalescence, i.e., two broad signals for **Aa** and **Ab**, or one averaged signal for both.

Due to its magnetic anisotropy, there is a strong NMR signal from the carbonyl position. The major conformers (**Aa** and **Ab**, or their average **A**) have a significantly higher chemical shift of H9b (5.40–5.55 *vs* 4.20–4.66 ppm) and a lower shift of C9 (34.6–35.1 *vs* 38.7–39.8 ppm). Conversely, the minor conformers (**Ba** and **Bb**, or their average **B**) have a higher chemical shift of H7 (5.39–5.44 versus 3.98–4.26 ppm) and a lower shift of C7 (42.1–42.7 ppm *vs* 48.6–48.8 ppm).

The orientation of the aryl group can be detected by observing the NOE of proton H16 in the NOESY spectrum. Based on the optimized molecular geometry, the **Aa** conformer would show the NOE on the methyl and proton H7, versus the protons H7 and H6b for the **Ab**. This specification enabled identification of the major conformers, i.e., **AM-10-Aa**, **AM-10-Ab**, **AM-12-Aa**, and **AM-12-Ab**. An analogous estimation for the minor conformers **Ba** and **Bb** failed due to low and overlapping signals.

Computation predicted four populated conformers from the computational study of compounds **AM-10** and **AM-15**, but spectroscopy detected only two conformers in the case of compound **AM-12**. Following the same designation of conformers as above, we present the corresponding populations and torsion angles in Table 5.

**Table 5:** Calculated Populations and Torsion Angles for **AM-10**, **AM-15**, and **AM-12**

conformer	population [%]	N2–N1–C17–N18 [°]	C7–N8–C10–C11 [°]	N8–C10–C11–C16 [°]
**AM-10-Ab**	40	160.0	5.4	92.0
**AM-10-Aa**	35	158.8	1.5	–97.1
**AM-10-Bb**	13	159.0	179.2	–107.4
**AM-10-Ba**	12	159.3	171.5	98.8
**AM-15-Aa**	36	159.4	9.2	–118.0
**AM-15-Ab**	32	158.6	–4.2	116.6
**AM-15-Bb1**	23	159.9	–176.3	–128.9
AM-15-Bb2	9	159.3	–178.5	–132.5
**AM-12-Ab**	60	159.4	2.6	96.1
**AM-12-Aa**	40	159.3	5.7	–101.9

As can be seen from the torsion angles, conformer **Ba** is absent for compound **AM-15**, and the two **Bb** conformers (designated as **1** and **2**) are very similar, with RMS for the overlay of only 0.14 Å. For compound **AM-12**, we found only **A** conformers; based on Gibbs free energies, the **B** conformers were unpopulated. For the geometries of populated conformers of compounds **AM-10**, **AM-15**, and **AM-12**, see Supporting Information Figures S33–S35, respectively. Cartesian coordinates of the preferred conformers are also available in the Supporting Information (Table S6).

In order to evaluate rotational flexibility around the C9–N8–C10–C11 (ExTor2) and N8–C10–C11–C16 (ExTor3) torsion angles, we performed free energy relaxed scans at the DFT level. Furthermore, to better assess the effect of substituents in the close vicinity to these torsions, two additional compounds were studied computationally, namely, ethyl (**Et-JNJ-55308942**) and isopropyl (^***i***^ **Pr-JNJ-55308942**) derivatives of **JNJ-55308942** at C7. Comparison of free energy maps of compounds **JNJ-55308942** and **AM-15** (see Figures 4 and S36a, respectively) shows that both maps are very similar, both in the positions of local minima and with respect to the barriers at low-energy transitions between them.

**Figure 4:**
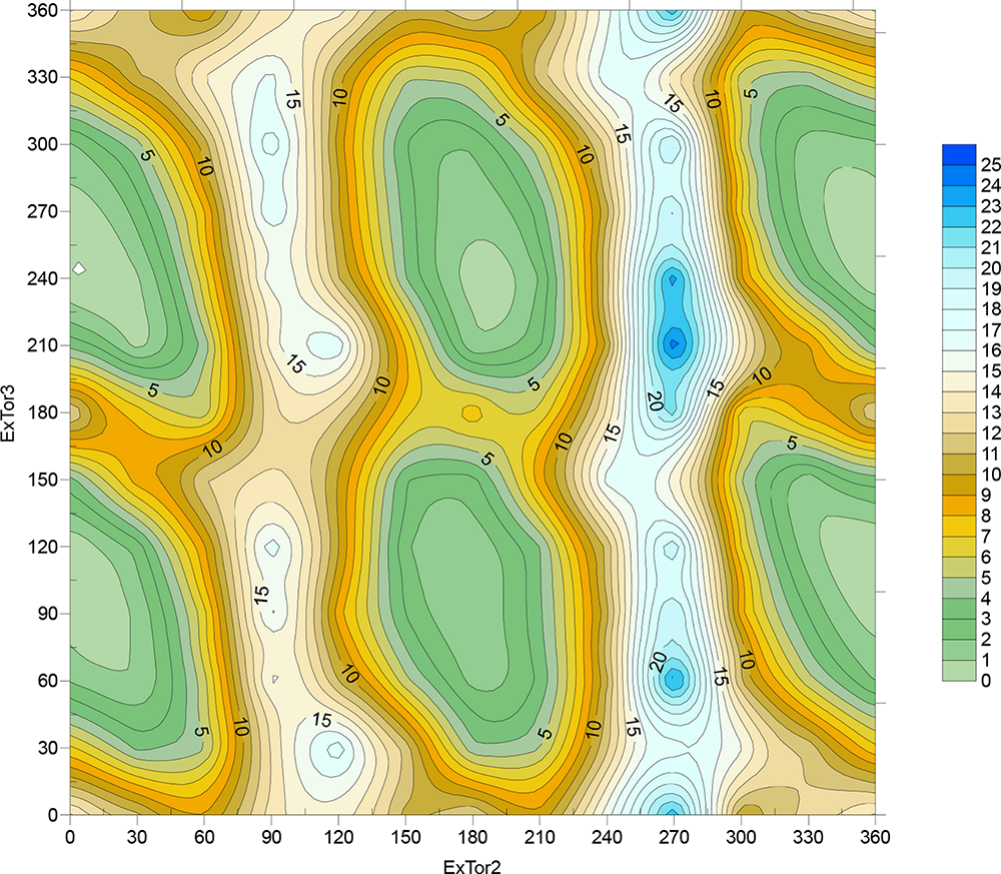
Relaxed scan free energy map of **JNJ-55308942** (energy is given in kcal/mol).

That clearly shows that the atom in position 14, either N or C, has a negligible effect on the flexibility of both torsions. On the contrary, the halogen atom in position 12 has a very significant effect on this flexibility. Energy maps for compounds **AM-10** and **AM-12** (see Figure S36b,c, respectively), with Cl or Br atoms in position 12, respectively, revealed much higher energy barriers on low-energy transitions between various rotamers. Calculations indicated energies of about 13 kcal/mol for compound **AM-10** compared to **JNJ-55308942**, and about 5 kcal/mol for compound **AM-12**. It is evident that a halogen atom larger than fluorine substantially hinders free rotation of vicinal torsions. Substitution of a methyl group in position 7 with an ethyl group in compound **Et-JNJ-55308942** did not lead to a significant change in the energy map (see Figure S36d) compared to **JNJ-55308942**. We conclude that an ethyl group can rotate in a manner not to hinder free rotation around adjacent exocyclic torsions. However, similar rotation of an isopropyl group is impossible, as can be seen from the energy map of compound ^***i***^ **Pr-JNJ-55308942** (see Figure S36e), where the energy barriers for low-energy transitions are about 5 kcal/mol higher compared to **Et-JNJ-55308942**. Free energy relaxed scans thus show that the halogen atom in position 12 is the structural feature having the largest effects on free rotation around the C9–N8–C10–C11 (ExTor2) and N8–C10–C11–C16 (ExTor3) torsion angles.

These considerations may call for an evaluation of the biological activity of the studied compounds (**JNJ-55308942**, **AM-15**, **AM-10**, and **AM-12**, and their derivatives) in light of the *in vivo* availability of their bioactive conformers. Assuming that only one conformation can access the binding site of the target receptor, it follows that the bioactivity of a given drug should depend directly on the content of the proper conformer and on the thermodynamics of its formation by isomerization.

## Conclusions

We demonstrate an efficient fluorine ^19^F/^18^F isotopic exchange without aromatic nucleophilic substitution of chlorine or bromine atoms by fluorine-18 in precursor candidates of tricyclic triazole-based ligands for PET examination of the P2X_7_ receptor. After due optimization, the ^19^F/^18^F isotopic exchange yielded sufficiently high molar activity for **[** ^**18**^ **F]­AM-10** (152 GBq/μmol, starting with 350 GBq of [^18^F]­fluoride) for quantitative autoradiography *in vitro* and, likewise, potentially for PET experiments in experimental animals and humans, without exceeding the conditions for tracer studies (usually <5% occupancy *in vivo*). The decay-corrected RCY was as high as 27% when lower molar activity was acceptable. Autoradiographic evaluation of **[** ^**18**^ **F]­AM-10** in mouse brain cryostat sections demonstrated high specific binding to brain P2X_7_ receptors, with maximal binding (*B*_max_) of 15.8 ± 2.8 pmol/g for the entire sagittal sections, and dissociation constant (*K*_D_) 16.6 ± 5.1 nM, which is in a range of other radioligands for P2X_7_ receptors.^11^ The P2X_7_ ligand [^11^C]­GSK1482160, which had low nM affinity binding in HEK cells, showed displaceable binding in the brains of living mice pretreated with lipopolysaccharide to evoke microglial activation;^25^ it remains to be established if **[** ^**18**^ **F]­AM-10** can likewise reveal specific binding in the living organism.

## Experimental Section

### Materials and Methods

For thin-layer chromatography (TLC), we used aluminum silica gel sheets for detection in UV light (TLC silica gel 60 F254, Merck). For column chromatography, we used 30–60 μm silica gel (ICN Biomedicals, Costa Mesa, USA). A JEOL 400 MHz spectrometer (Peabody, USA) served for basic NMR spectra, and a JEOL 500 MHz instrument (Peabody, USA) provided spectra as a function of temperature. The signals are represented by chemical shift (δ) in ppm, followed by multiplicity and corresponding coupling constants (*J*) in Hz, and by signal assignment, which is based on an analysis of ordinary ^1^H–^1^H COSY, ^1^H–^1^H NOESY, H–^13^C and ^1^H–^15^N HSQC, and on ^1^H–^13^C and ^1^H–^15^N HMBC correlation spectra. The ^1^H and ^13^C chemical shifts are referenced to TMS (using the solvent signals from CHD_2_SOCD_3_ (2.50 ppm) and CD_3_SOCD_3_ (39.52 ppm). The ^15^N and ^19^F chemical shifts are referenced (using the frequency of solvent deuterium) to liquid NH_3_ and CFCl_3_, respectively.

Quadrupole LC/MS (ESI ionization) with an Infinity III LC system (Agilent Technologies, Santa Clara, USA) was used for LR-MS and HPLC analyses (C18 column: 100 mm; UV detection). We used HPLC analyses (C18 column, UV and RAD detection) for the characterization of radioactive products and semipreparative HPLC (C18, UV and RAD detections) for purification. We performed radiosynthesis and purification procedures manually or on the GE TRACERlab FX FN synthesizer. For autoradiography, we used FAD mouse brains, Tissue-Tek gel (Sakura, Torrance, CA, USA), Superfrost Plus microscope slides (Hampton, USA), and a Microtome cryostat (Leica Biosystems, USA).

The following chemicals were purchased from commercial sources and used as delivered: Abcr GmbH (Karlsruhe, Germany): 3-fluoro-2-(trifluoromethyl)­isonicotinic acid (95%), 1-*N*-boc-(*S*)-2-methylpiperidine-4-one (95%), and 2-bromo-3-(trifluoromethyl)­benzoic acid (98%); Fluorochem Ltd. (Hadfield, UK): 3-chloro-2-(trifluoromethyl)­isonicotinic acid (97%); Sigma-Aldrich (Missouri, USA): ethylene glycol-bis­(β-aminoethyl ether)-*N*,*N*,*N′*,*N′*-tetraacetic acid – EGTA (≥97.0%), magnesium chloride (≥98%), *m*-chloroperoxybenzoic acid – *m*-CPBA (≥77%), oxalyl chloride – (COCl)_2_ (≥99%), pyrrolidine (99%), sodium bicarbonate (99.5%), sodium sulfite (≥98%), tetrabutylammonium methanesulfonate – Bu_4_NOMs (≥97%), triethylamine – TEA (99.5%), and tris­(hydroxymethyl)­aminomethane hydrochloride (≥99%); and TCI Europe (Zwijndrecht, Belgium): 2-fluoro-3-(trifluoromethyl)­benzoic acid (≥98%).

#### [^18^F]-(*S*)-(3-(Fluoro)-2-(trifluoromethyl)­pyridin-4-yl)­(6-methyl-1-(pyrimidin-2-yl)-1,4,6,7-tetrahydro-5*H*-[1,2,3]­triazolo­[4,5-*c*]­pyridin-5-yl)­methanone (**[^18^F]­JNJ-64413739**)

##### Manual Synthesis

N.c.a. [^18^F]­fluoride was produced *via* ^18^O­(p,n)^18^F nuclear reaction by proton irradiation (18 MeV 80 μA) of enriched [^18^O]­H_2_O. Irradiated [^18^O]­H_2_O from the cyclotron target (TR-24 cyclotron, Nb-target, ACSI, Canada) was passed through a preconditioned QMA cartridge (10 mL, 0.5 M K_2_CO_3_) to trap [^18^F]­fluoride. Subsequently, the [^18^F]­fluoride was eluted with 1 mL 20 mM NBu_4_OMs in MeOH (Merck, Germany), and then dried by azeotropic distillation using N_2_ at 120 °C and the addition of dry MeCN (2 × 0.5 mL). For experimental details and RCYs, see Table 1.

##### Radiosynthesis Using the TRACERlab FX FN Synthesizer

Irradiated [^18^O]­H_2_O from the cyclotron target was passed through a preconditioned QMA cartridge (10 mL 0.5 M K_2_CO_3_ or 0.25 M K_2_C_2_O_4_, 20 mL water (WFI) to trap the [^18^F]­fluoride. Subsequently, [^18^F]­fluoride was eluted with 1 mL eluent (15 mg Kryptofix 222, 5.8 mg K_2_C_2_O_4_, 0.04 mg K_2_CO_3_) in 50% MeCN. After elution, the [^18^F]­fluoride was dried by azeotropic distillation using N_2_ at 120 °C and the addition of dry MeCN (2 × 0.5 mL). To the dried [^18^F]­fluoride was added 1–3 mg of precursor **JNJ-66410047** in 0.6 mL of DMSO. The mixture was heated (135 °C, 10 min) and then diluted with 2 mL of mobile phase MeCN: 20 mM-phosphate buffer pH 5 30/70 (*v*/*v*). The reaction mixture was injected into the semipreparative HPLC, and the reactor was washed with 2 mL of the mobile phase.

The reaction mixture was analyzed on a semipreparative HPLC (Atlantis Prep T3 column, 250 × 10 mm^2^, Waters; MeCN: 20 mM-phosphate buffer pH 5 30/70 (*v*/*v*; 8 mL/min). The radioactive peak fraction at 15–16 min was diluted with 100 mL of distilled water, and the mixture was passed through a preconditioned tC18 Plus cartridge (Waters, USA; 5 mL of EtOH, 20 mL of H_2_O), eluted with 1 mL of EtOH, and diluted with 10 mL of saline. **[** ^**18**^ **F]­JNJ-64413739** was isolated in 2.1–6.1% radiochemical yield (RCY), > 99% radiochemical purity (RCP), and a total synthesis time of 70 min. The molar activity was not determined due to the absence of the chromatographic standard. The radiolabeled product was identified by HPLC-MS.

### General Procedure (GP) for *N*-Acylation

To a solution of (hetero)­aromatic benzoic acid (1 equiv) in DCM (2 mL) was added (COCl)_2_ (2 equiv). One drop of DMF was added, and the mixture was stirred for 2 h at RT. The solvents were removed under reduced pressure, and the residue was coevaporated with toluene (3 × 5 mL). Crude chloride salt was dissolved in DCM (2–4 mL) and added to amine **s5** (0.5 equiv) in DCM (2 mL). TEA (2 equiv) was added *via* a syringe, and the mixture was stirred overnight at RT. The solvents were then evaporated under reduced pressure, and after aqueous workup, the residue was chromatographed (2–3% MeOH-DCM).

#### (*S*)-(3-Fluoro-2-(trifluoromethyl)­pyridin-4-yl)­(1-(5-fluoropyrimidin-2-yl)-6-methyl-1,4,6,7-tetrahydro-5*H*-[1,2,3]­triazolo­[4,5-*c*]­pyridin-5-yl)­methanone (**JNJ-55308942**)

**JNJ-55308942** (34 mg, 0.08 mmol) was prepared by the GP from **s5** (47 mg, 0.2 mmol) in 40% yield. *R*_F_ = 0.3 in DCM-MeOH 20:1 (*v*/*v*). MS (ESI): for C_17_H_12_F_5_N_7_O calcd 425.10 Da, found *m*/*z* 426.1 [M + H]^+^. ^1^H, ^13^C, and ^19^F NMR characteristics are depicted in Supporting Information Table S2 and NMR spectra are in Figures S2–S7.

#### (*S*)-(3-Chloro-2-(trifluoromethyl)­pyridin-4-yl)­(1-(5-fluoropyrimidin-2-yl)-6-methyl-1,4,6,7-tetrahydro-5*H*-[1,2,3]­triazolo­[4,5-*c*]­pyridin-5-yl)­methanone (**AM-10**)

**AM-10** (60 mg, 0.14 mmol) was prepared by GP from **s5** (0.2 mmol) in 68% yield. *R*_F_ = 0.7 in AcOEt-MeOH 20:1 (*v*/*v*). MS (ESI): for C_17_H_12_ClF_4_N_7_O calcd 441.07 Da; found *m*/*z* 442.0 [M + H]^+^. ^1^H, ^13^C, and ^19^F NMR characteristics are depicted in Supporting Information Table S3 and studied spectra are in Figures S8–S17.

#### (*S*)-(2-Bromo-3-(trifluoromethyl)­phenyl)­(1-(5-fluoropyrimidin-2-yl)-6-methyl-1,4,6,7-tetrahydro-5*H*-[1,2,3]­triazolo­[4,5-*c*]­pyridin-5-yl)­methanone (**AM-12**)

**AM-12** (40 mg, 0.08 mmol) was prepared by the GP from **s5** (0.2 mmol) in 41% yield. *R*_F_ = 0.7 in AcOEt-MeOH 20:1 (*v*/*v*). MS (ESI): for C_18_H_13_BrF_4_N_6_O calcd 484.03 Da; found *m*/*z* 485.0 [M + H]^+^. ^1^H, ^13^C, and ^19^F NMR characteristics are depicted in Supporting Information Table S4 and studied spectra are in Figures S18–S25.

#### (*S*)-(2-Fluoro-3-(trifluoromethyl)­phenyl)­(1-(5-fluoropyrimidin-2-yl)-6-methyl-1,4,6,7-tetrahydro-5*H*-[1,2,3]­triazolo­[4,5-*c*]­pyridin-5-yl)­methanone (**AM-15**)

**AM-15** (38 mg, 0.09 mmol) was prepared by GP from **s5** (0.2 mmol) in 45% yield. *R*_F_ = 0.3 in DCM-MeOH 20:1 (*v*/*v*). MS (ESI): for C_18_H_13_F_5_N_6_O calcd 424.11 Da; found *m*/*z* 425.0 [M + H]^+^. ^1^H, ^13^C, and ^19^F NMR characteristics are depicted in Supporting Information Table S5 and studied spectra are in Figures S26–S32.

#### [^18^F]-(*S*)-(3-Chloro)-2-(trifluoromethyl)­pyridin-4-yl)­(1-(5-fluoropyrimidin-2-yl)-6-methyl-1,4,6,7-tetrahydro-5*H*-[1,2,3]­triazolo­[4,5-*c*]­pyridin-5-yl)­methanone (**[^18^F]­AM-10**)

##### Manual Synthesis

For the production of n.c.a. [^18^F]­fluoride, see the synthesis of **[** ^**18**^ **F]­JNJ-64413739** above. To the dried [^18^F]­fluoride was added **AM-10** (0.2 mg, 0.45 μmol) in 0.9 mL of DMSO. The mixture was heated to 135 °C for five min, whereupon the reaction mixture was quenched with 2 mL of distilled water. The reaction mixture was analyzed on a semipreparative HPLC (Luna C18 column, 250 × 10 mm; 45% MeCN, 0.1% TFA, 4 mL/min). The fraction at 600 s was reinjected to the HPLC along with a cold reference for compound identification (Luna 5 μm C18 column, 150 × 4.6 mm; 25–95% MeCN in 7 min, 0.1% TFA, 1.5 mL/min, *R*_T_ = 5.5 min). Subsequently, the fraction was diluted with 50 mL of distilled water, passed through a preconditioned C18 cartridge (5 mL; 50% EtOH), and eluted with 3 mL of EtOH. **[** ^**18**^ **F]­AM-10** was isolated in 4.7% radiochemical yield (RCY) and 99.9% radiochemical purity (RCP) with a 9.26 MBq/μmol specific molar activity (SA) in a total synthesis time of 50 min.

##### Radiosyntheses Using the TRACERlab FX FN Synthesizer

N.c.a. [^18^F]­fluoride was produced *via* the ^18^O­(p,n)^18^F nuclear reaction, as described above. The radionuclide was eluted into the reactor. After elution with 1 mL of 20 mM Bu_4_NOMs (Merck, Germany) in MeOH, [^18^F]­fluoride was dried as described above. To the dried [^18^F]­fluoride was added **AM-10** (200 μg, 0.45 μmol) in 0.6 mL of DMSO. The mixture was heated to 135 °C for 8 min and then diluted with 2 mL of the mobile phase. The reaction mixture was injected into the semipreprative HPLC, and the reactor was washed with 2 mL of the mobile phase.

HPLC analysis was performed on a Kinetex EVO C18 column (100 × 21.2 mm^2^, Phenomenex) with the eluent MeOH/20 mM-phosphate buffer pH 7.0 57/43 (*v*/*v*) delivered at 5 mL/min. The radioactive peak fraction at 13–16 min was diluted with 100 mL of distilled water, and the mixture was passed through a preconditioned tC18 Plus cartridge (Waters, USA; 5 mL of EtOH, 20 mL of H_2_O) and eluted with 1 mL of EtOH. The diluted saline was eluted just before use.

#### [^18^F]-(*S*)-(2-Bromo-3-(trifluoromethyl)­phenyl)­(1-(5-fluoropyrimidin-2-yl)-6-methyl-1,4,6,7-tetrahydro-5*H*-[1,2,3]­triazolo­[4,5-*c*]­pyridin-5-yl)­methanone (**[^18^F]­AM-12**)

*Manual synthesis.* For the production of n.c.a. [^18^F]­fluoride, see the synthesis of **[** ^**18**^ **F]­JNJ-64413739** above. To the dried [^18^F]­fluoride was added **AM-12** (2.8 mg, 5.4 μmol) in 0.9 mL of DMSO, and the mixture was heated to 135 °C. After heating for 2, 5, and 10 min, 30 μL of samples was taken and diluted with 70 μL of water, and after 18 min at RT, the reaction mixture was quenched with 2 mL of water. Along with cold reference standards for compound identification, the samples and the reaction mixture were injected into the HPLC (Kinetex 2.6 μm C18 column, 150 × 4.6 mm; 35% MeCN, 0.1% TFA, 1.5 mL/min, *R*_T_ = 4.1 min). We did not isolate **[** ^**18**^ **F]­AM-12**; its quantitation by TLC indicated 73% radiochemical conversion (RCC) at 2 min.

### Ab Initio Calculations

For computational studies, low-energy conformers for all compounds (**JNJ-55308942** and **AM-10**, **AM-12**, and **AM-15**) were searched using the Conformational Search routine in the MacroModel module^21^ at the molecular mechanics level using the OPLS4 force field.^22^ Found structures were optimized at the density functional theory (DFT) level with the B3LYP-D3 ^23^ functional and the 6–31G** basis set in implicit water using the CPCM^24^ solvent model. All of the identified local minima were verified by a frequency calculation at the same theoretical level, and Gibbs free energies were used for the estimation of Boltzmann populations of each conformer *p*_*i*_ according to

$$p_{i}=\frac{\exp\left(-\frac{\varepsilon_{i}}{kT}\right)}{\underset{i}{\sum }\exp\left(-\frac{\varepsilon_{i}}{kT}\right)}$$

where ε_*i*_ is the Gibbs free energy of the *i*-th conformer, *k* is the Boltzmann constant, and *T* is the absolute temperature. All DFT calculations were performed in the Jaguar package.^21^

### In Vitro Autoradiography

Brains harvested from male mice of C57BL/6N background were placed on ice prior to immersion in isopentane (−40 °C), followed by storage at −80 °C. A cerebral hemisphere was placed on the cryotome stand and frozen in place with tissue gel Tissue-Tek O.C.T. Compound (Sakura) for cutting at −20 °C. Serial 20 μm-thick sagittal brain sections were mounted on Superfrost Plus adhesive glass slides (Fischer) and stored at −80 °C until use. After thawing and preincubation for 10 min (50 mM Tris-HCl, pH 7.4, 5 mM MgCl_2_, 2 mM EGTA, 0.1% BSA), the sections were incubated for 1 h with 0.3; 1, 3; and 10 nM **[^1^** **^8^F]­AM-10** solutions. Nonspecific binding was assessed on consecutive sections in the additional presence of 10 μM unlabeled GSK1482160 as a blocking agent. Following the washing steps (3 × 1 min in ice-cold incubation buffer and a 30 s dip in distilled water) and drying at room temperature, the brain sections and slides containing spots of known radiochemical concentration were exposed overnight to a phosphor storage screen (Cyclone Plus (PerkinElmer) to obtain autoradiograms. After quantitation of the total nonspecific binding, Scatchard analysis of the specific binding component gave estimates of the saturation binding parameters *B*_max_ and *K*_D_.

## Ethics Statement

The animal study protocol was approved by the Ethics Committee of Samo Biomedical Centre, Pardubice, Czech Republic, for studies involving animals.

## Supplementary Material



## Notes

Reported P2X_7_ ligand values of dissociation constant (*K*_D_) and specific binding (*B*_max_); synthetic procedures; NMR spectra, conformational behavior; and DFT data (PDF)

## ○

Department of Clinical Physiology, Nuclear Medicine and PET, Rigshospitalet, Blegdamsvej 9, 2100 Copenhagen, Denmark

## ◆

RadioMedic, Husinec - Řež 289, 250 68 Řež, Czech Republic

## ¶

Czech Institute of Informatics, Robotics and Cybernetics, Czech Technical University in Prague, Jugoslávských partyzánů 1580/3, 160 00 Praha, Czech Republic

## )

Samo Biomedical Centre, Na Klínku 1082, 530 06 Pardubice, Czech Republic
