# Supplementary material for: Triazole-Based Radioligands for PET of P2X7R: Syntheses, Conformational Studies, and Preliminary Autoradiographic Evaluation of [18F]AM-10
Source: ACS Omega. 2025 Aug 6;10(32):36340–50. doi: 10.1021/acsomega.5c04531 (PMC12368693; doi:10.1021/acsomega.5c04531)
Supplement: Supplementary file 1 [file ao5c04531_si_001.pdf]

Triazole-Based Radioligands for PET of P2X$_7$R: Syntheses, Conformational Studies and Preliminary Autoradiographic Evaluation of [^{18}F]AM-10

Anna Marešová[1], Michal Jurášek[1*], Ivan Raich[1], Bohumil Dolenský[2], Vladimir Shalgunov[3,4], Matthias Manfred Herth[3,4], Petr Džubák[5,6], Libor Procházka[7,8], Hana Vinšová[7,13], Daniel Seifert[7,9], Ondřej Lebeda[7], Pavel B. Drašar[1], Paul Cumming[10,11] and Alexander Popkov[12,13*]

[1] Department of Chemistry of Natural Compounds, University of Chemistry and Technology, Technická 5, 160 00 Prague, Czech Republic

[3] Department of Drug Design and Pharmacology, University of Copenhagen, Jagtvej 162, 2100 Copenhagen, Denmark

[5] Institute of Molecular and Translational Medicine (IMTM), Faculty of Medicine and Dentistry, Palacký University and University Hospital in Olomouc, Hněvotínská 1333/5, 779 00 Olomouc, Czech Republic

[6] Laboratory of Experimental Medicine, IMTM, University Hospital Olomouc, Hněvotínská 976/3, 779 00 Olomouc, Czech Republic

[7] Department of Radiopharmaceuticals, Nuclear Physics Institute of the Czech Academy of Sciences, Husinec - Řež 130, 250 68 Řež, Czech Republic

[10] Department of Nuclear Medicine, University Hospital Bern, Freiburgstrasse 18, 3010 Bern, Switzerland

[11] School of Psychology and Counselling, Queensland University of Technology, Kelvin Grove QLD 4059, Australia

[12] Institute of Organic Chemistry, Johannes Kepler University Linz, Altenberger Straße 69, 4040 Linz, Austria

[13] Samo Biomedical Centre, Na Klínku 1082, 530 06 Pardubice, Czech Republic

Corresponding authors:
Alexander Popkov, e-mail: oleksandr.popkov@jku.at
Michal Jurášek, e-mail: michal.jurasek@vscht.cz

[4] Current address: Department of Clinical Physiology, Nuclear Medicine and PET, Rigshospitalet, Blegdamsvej 9, 2100 Copenhagen, Denmark

[8] Current address: RadioMedic, Husinec - Řež 289, 250 68 Řež, Czech Republic

[9] Current address: Czech Institute of Informatics, Robotics and Cybernetics, Czech Technical University in Prague, Jugoslávských partyzánů 1580/3, 160 00 Praha, Czech Republic

[13] Current address: Samo Biomedical Centre, Na Klínku 1082, 530 06 Pardubice, Czech Republic

Table of content

1. Reported $P2X_7$ ligand values of dissociation constant (K_D) and specific binding (B_{max}) S-3
2. Supplementary synthetic procedures S-4
3. NMR spectra, conformational behavior and DFT data S-7

1. Reported P2X$_7$ ligand values of dissociation constant (K$_D$) and specific binding (B$_{max}$)

Table S1. Table of reported P2X$_7$ ligand values dissociation constant (K$_D$) and specific binding (B$_{max}$).

Compound	K$_D$ [nM]	B$_{max}$	Where?	Method	Ref.
[11C]GSK1482160 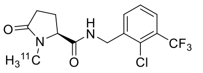 [11C]GSK1482160	5.09 ± 0.98	5520 cpm/1×10^6 cells	HEK293 cells expressing human recombinant P2X7 receptors	Cells binding assay	s1
	1.15 ± 0.12	3.03 ± 0.10 pmol/mg	HEK293 cells expressing human recombinant P2X7 receptors	Homogenate binding assay	s2
	1.4 ± 0.15	5.43 ± 0.55 pmol/mg protein	HEK293 cells expressing human recombinant P2X7 receptors	Homogenate binding assay	s3
	1.1 ± 0.25	5.73 ± 0.55 pmol/mg protein	U-2 cells expressing human recombinant P2X7 receptors		
[123I]TZ6019 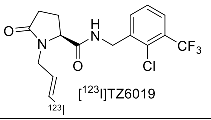 [123I]TZ6019	19.3 ± 2.8	262±10 pmol/g	HEK293 cells expressing human recombinant P2X7 receptors	Intact cell binding assay	s4
[11C]SMW139 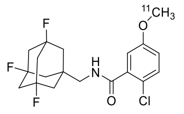 [11C]SMW139	20.6 ± 1.7	N/A	HEK293 cells expressing rat recombinant P2X7 receptors	Homogenate binding assay	s5
	4.6 ± 0.8	N/A	HEK293 cells expressing human recombinant P2X7 receptors		
[3H]A-804598 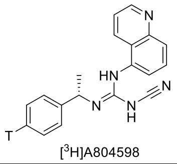 [3H]A804598	2.4 ± 0.51	0.56 ± 0.11 pmol/mg protein	1321N1 cells expressing recombinant rat P2X7 receptors	Cell membrane binding assay	s6
	3.08 ± 0.56	112.90 ± 5.57 pmol/g	Rat cortex	Homogenate binding assay	s7
[3H]JNJ-54232334 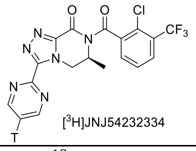 [3H]JNJ54232334	4.9 ± 1.3	350 pmol/g protein	Rat brain	Homogenate binding assay	s8
[18F]FTTM 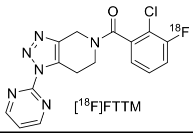 [18F]FTTM	25.35 ± 3.47	22.77 fmol/cell	RAW264.7 mouse macrophage cells	Intact cell binding assay	s9
[18F]AM-10 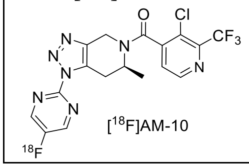 [18F]AM-10	16.6 ± 5.1	15.8 ± 2.8 pmol/g	Mice brain slices	Autoradiography binding assay	Our article

2. Synthetic procedures

Reproduction of the synthesis of [^{18}F]JNJ-64413739 according to Kolb *et al.*[s10]

i. (*S*)-1-(3-Chloro-2-(trifluoromethyl)isonicotinoyl)-2-methylpiperidin-4-one (**s1**). To a solution of 3-chloro-2-(trifluoromethyl)isonicotinic acid (360 mg, 1.6 mmol) in DCM (5 mL) $(COCl)_2$ (1 mL) and three drops of DMF were added. The mixture was stirred at RT for 17 h. The solvents were evaporated under reduced pressure and the residue was co-evaporated with toluene (3×5 mL). The crude chloride thus obtained was dissolved in DCM (15 mL), to which (*S*)-2-methyl-4-oxopiperidine hydrochloride (240 mg, 1.6 mmol) and TEA (0.5 mL) were added, respectively. The mixture was stirred for 3 h at RT, followed by solvent removal under reduced pressure. The residue was purified by column chromatography ($CHCl_3$-MeOH 99:1, *v/v*). Compound **s1** (414 mg, 1.29 mmol) was obtained as a slightly yellowish solid in 81% yield. MS (ESI): for $C_{13}H_{12}ClF_3N_2O_2$ calcd 320.1 Da, found *m*/*z* 321.0 $[M+H]^+$.

ii. (*S*)-(3-Chloro-2-(trifluoromethyl)pyridin-4-yl)(6-methyl-1-(pyrimidin-2-yl)-1,4,6,7-tetrahydro-5*H*-[1,2,3]triazolo[4,5-*c*]pyridin-5-yl)methanone (**JNJ-64410047**). Compound **s1** (200 mg, 0.62 mmol) was dissolved in toluene (5 mL) and heated to 110 °C. A solution of 2-azidopyrimidine (98 mg, 0.81 mmol) in toluene (5 mL) was added by syringe, followed by addition of pyrrolidine (56 µL, 0.68 mmol). The resulting mixture was stirred at 110 °C for 4 h, followed by addition of DCM (5 mL) and cooling to 0 °C on an ice bath. Solid $NaHCO_3$ (104 mg, 1.24 mmol) and *m*-CPBA (214 mg, 1.24 mmol) were then added, and the mixture was removed from the ice bath. After addition of 1N NaOH (15 mL) the mixture was stirred for 30 min. The aqueous layer was separated and extracted with DCM (3×50 mL), and the combined organic layer was dried over Na_2SO_4 followed by evaporation of the solvents under reduced pressure. The residue was purified twice by column chromatography ($CHCl_3$-MeOH 99:1, *v/v*). The product **JNJ-64410047** (157 mg, 0.37 mmol) was obtained as a slightly yellowish solid in 59% yield. **^{1}H NMR** (400 MHz, $CDCl_3$) δ: 8.88 – 8.80 (m, 2H), 8.70 – 8.58 (m, 1H), 7.50 – 7.34 (m, 2H), 5.81 – 5.51 (m, 1H), 4.67 – 4.26 (m, 2H), 4.02 – 3.92 (m, 1H), 3.54 – 3.16 (m, 2H), 1.33 – 1.19 (m, 6H). MS (ESI): for $C_{17}H_{13}ClF_3N_7O$ calcd 423.1 Da, found *m*/*z* 424.1 $[M+H]^+$.

2-Azido-5-fluoropyrimidine (**s2**). *i.* To a solution of 2-chloro-5-fluoropyrimidine (4 g, 30 mmol) in EtOH (40 mL) hydrazine monohydrate (9 mL) was added *via* syringe. The mixture was heated to 80 °C in a thermoblock for 5 h. After removal from heating, the solvents were removed under reduced pressure to a half volume. The remaining volume was poured into water (200 mL) and the product was extracted with DCM (5 × 30 mL). The combined organic layers were dried over $MgSO_4$, filtered, and the solvents removed under reduced pressure. We obtained the hydrazine intermediate (2.4 g, 18.7 mmol) as a white solid in 62% yield. R_F = 0.2 in CyHex-AcOEt 1:1 (*v*/*v*). *ii.* The hydrazine intermediated (2.4 g) was transferred to a 500 mL round bottom flask, and dissolved in HOAc (30 mL) and water (15 mL). The

mixture was cooled to 0 °C (ca. 20 min) followed by addition of sodium nitrite (1.94 g, 28 mmol) dissolved in water (5 mL) dropwise *via* a Pasteur pipette. The mixture was stirred for 1 h at 0 °C, and then diluted with diethyl ether/petroleum ether mixture (1:1 *v/v*; 50 mL). After adjusting the pH by addition of 1 N NaOH (pH~11), the organic layer was removed, and the aqueous layer was extracted with diethyl ether (3×50 mL). The combined organic layers were dried over $MgSO_4$, filtered, and the solvents evaporated under reduced pressure. The residue was passed through a short silica gel column (petroleum ether-diethyl ether 4:1 *v/v*). The azide **s1** (1.5 g, 10.8 mmol) was obtained as an opalescent liquid in 58% yield. The yield of **s1** over two steps was 36 %. R_F = 0.4 in CyHex-AcOEt 5:1 (*v/v*). ^{1}H NMR (400 MHz, $CDCl_3$) δ ppm: 8.46 (s, 2 H). ^{13}C NMR (101 MHz, $CDCl_3$) δ ppm: 146.92, 155.18, 158.13. ^{19}F NMR (376 MHz, $CDCl_3$) δ: -144.30. MS (ESI): for $C_4H_2FN_5$ calcd 139.09 Da, found *m/z* 140.0 $[M+H]^+$.

tert-Butyl (*S*)-1-(5-fluoropyrimidin-2-yl)-4-methyl-1,4,6,7-tetrahydro-5*H*-[1,2,3]triazolo[4,5-*c*]pyridine-5-carboxylate (**s3**) and *tert*-butyl (*S*)-1-(5-fluoropyrimidin-2-yl)-6-methyl-1,4,6,7-tetrahydro-5*H*-[1,2,3]triazolo[4,5-*c*]pyridine-5-carboxylate (**s4**). To a preheated solution of 1-*N*-boc-2(*S*)-methylpiperidine-4-one (1.5 g, 7.0 mmol) in toluene (40 mL) at 100 °C, 2-azido-5-floropyrimidine **s1** (1.3 g, 9.3 mmol) in toluene (6 mL) and pyrrolidin (500 mg, 7.0 mmol) in toluene (3 mL) were added *via* syringe, respectively. The mixture was stirred at 100 °C for 20 min. After removal from heating, the mixture was stirred ON at RT. The mixture was then cooled on an ice bath and diluted with DCM (30 mL), followed by addition of solid $NaHCO_3$ (1.5 g, 17.5 mmol) and *m*-CPBA (2.7 g, 15.4 mmol). The resultant mixture was stirred for 30 min at RT and then poured into 1 M NaOH (100 mL). The product was extracted with DCM (4 × 30 mL), and the combined organic layer washed with brine (1×50 mL). The separated organic layer was dried over $MgSO_4$, filtered, and the solvents evaporated under reduced pressure. The residue was purified by column chromatography (CyHex-AcOEt 3:1→2:1→1:1 *v/v*; column 30 × 160 mm). $\mathbf{R_F}$ = 0.4 in AcOEt-petroleum ether 1:1 (*v/v*). The mass of the matter after first chromatography (1.1 g, 3.3 mmol) corresponding to a total 47% yield. **MS (ESI)**: for $C_{15}H_{19}FN_6O_2$ calcd 334.16 found *m/z* 335.2 $[M+H]^+$. A second chromatography was performed with $CHCl_3$-MeOH 100:1 (*v/v*; column 30 × 300 mm). After identifying the product elution by TLC in the $CHCl_3$-MeOH 50:1 (*v/v*) system, we collected 20 mL fractions. All five fractions had the same $\mathbf{R_F}$, i.e. 0.3 in $CHCl_3$-MeOH 50:1 (*v/v*), Fractions F1-F3 were identified by NMR to contain **s4** of sufficient purity and were combined, yielding pure **s4** (556 mg, 1.7 mmol) in 24% yield after the second chromatography. The combined F4 and F5 (526 mg) was chromatographed once again in $CHCl_3$-MeOH 100:1 (*v/v*; column 30 × 200 mm). Five consecutive fractions (20 mL) with product were collected, evaporated under reduced pressure, and evaluated by NMR. Fraction F1 contained pure **s4** isomer (189 mg, 0.57 mmol), F2 (188 mg) was a mix of **s4** and **s3**, and F3 was almost pure **s3** isomer (125 mg, 0.37 mmol). The F3 fraction was dissolved in MeOH and crystalized at 4 ºC to obtain analytically pure compound **s3** (30

mg). The total isolated yields were 0.05% for **s3** and 32% for **s4**. Triazole **s3**. **^{1}H NMR** (400 MHz, $CDCl_3$, Fig S1A) δ: 8.71 (s, 2H), 5.39 (s, 1H), 4.48 (s, 1H), 3.33 – 3.15 (m, 1H), 3.15 – 2.98 (m, 2H), 1.51 (d, *J* = 6.8 Hz, 3H), 1.48 (s, 9H). **^{13}C NMR** (101 MHz, $CDCl_3$, Figure S1B) δ: 156.71, 154.53, 151.52, 146.97, 132.07, 80.49, 47.00, 35.90, 28.52, 24.59, 18.97. **^{19}F NMR** (376 MHz, $CDCl_3$) δ: -137.78. Triazole **s4**. **^{1}H NMR** (400 MHz, $CDCl_3$, Fig S1A) δ: 8.68 (s, 2H), 5.09 (d, *J* = 16.6 Hz, 1H), 4.89 (s, 1H), 4.19 (d, *J* = 16.5 Hz, 1H), 3.26 (dd, *J* = 17.6, 6.1 Hz, 1H), 3.09 (d, *J* = 17.5 Hz, 1H), 1.43 (s, 9H), 1.10 (d, *J* = 7.0 Hz, 3H). **^{13}C NMR** (101 MHz, $CDCl_3$, Figure S1B) δ: 156.65, 154.59, 151.40, 146.89, 141.05, 131.17, 80.52, 44.19, 37.21, 29.70, 28.39, 17.61. **^{19}F NMR** (376 MHz, $CDCl_3$) δ: -137.73.

(*S*)-1-(5-Fluoropyrimidin-2-yl)-6-methyl-4,5,6,7-tetrahydro-1*H*-[1,2,3]triazolo[4,5-*c*]pyridine (**s5**). To a solution of **4s** (500 mg, 1.5 mmol) in DCM (10 mL), HCl (1.5 mL) dissolved in 1,4-dioxane (5 mL) was added *via* Pasteur pipette. After 30 min, MeOH (5 mL) was added and the mixture stirred for 3 days at RT. The solvents were evaporated under reduced pressure. The residue was dissolved in DCM (20 mL) and MeOH (2 mL), whereupon saturated $NaHCO_3$ solution (30 mL) was added to the mixture. After stirring at RT for 30 min, the organic layer was removed, and the aqueous layer was extracted with DCM (5×10 mL). The combined organic layers were dried over $MgSO_4$, filtered, and the solvent removed under reduced pressure to obtain **s5** (312 mg, 1.3 mmol, 87%) as a yellowish honey. The honey was dissolved in AcOEt (*ca* 3 mL) and left at 4 ºC ON. The resultant solids (101 mg, 0.43 mmol) were filtered on a paper, and dried under reduced pressure. After evaporating the mother liquor, the product was re-crystalized from AcOEt to obtain another crop (70 mg, 0.3 mmol) of solid amine. Combining the two crystallizations, we obtained 49% (0.73 mmol) of the pure amine. **^{1}H NMR** (400 MHz, $CDCl_3$, Figure S1A) δ: 8.69 (s, 2H), 4.17 (dd, *J* = 15.3, 1.3 Hz, 1H), 4.07 (dt, *J* = 15.3, 2.0 Hz, 1H), 3.24 (ddd, *J* = 17.3, 4.0, 1.8 Hz, 1H), 3.04 (dqd, *J* = 10.3, 6.4, 4.0 Hz, 1H), 2.70 (dddd, *J* = 17.3, 9.9, 2.2, 1.4 Hz, 1H), 1.65 (s, 1H), 1.32 (d, *J* = 6.4 Hz, 3H). **^{13}C NMR** (101 MHz, $CDCl_3$, Figure S1B) δ: 156.60, 151.62, 146.85, 143.88, 133.01, 49.04, 42.26, 32.92, 21.83. **^{19}F NMR** (376 MHz, $CDCl_3$) δ: -138.19. MS (ESI): for $C_{10}H_{11}FN_6$ calcd 234.10 Da, found *m/z* 235.1 $[M+H]^+$.

3. NMR spectra, conformational behavior and DFT data

A

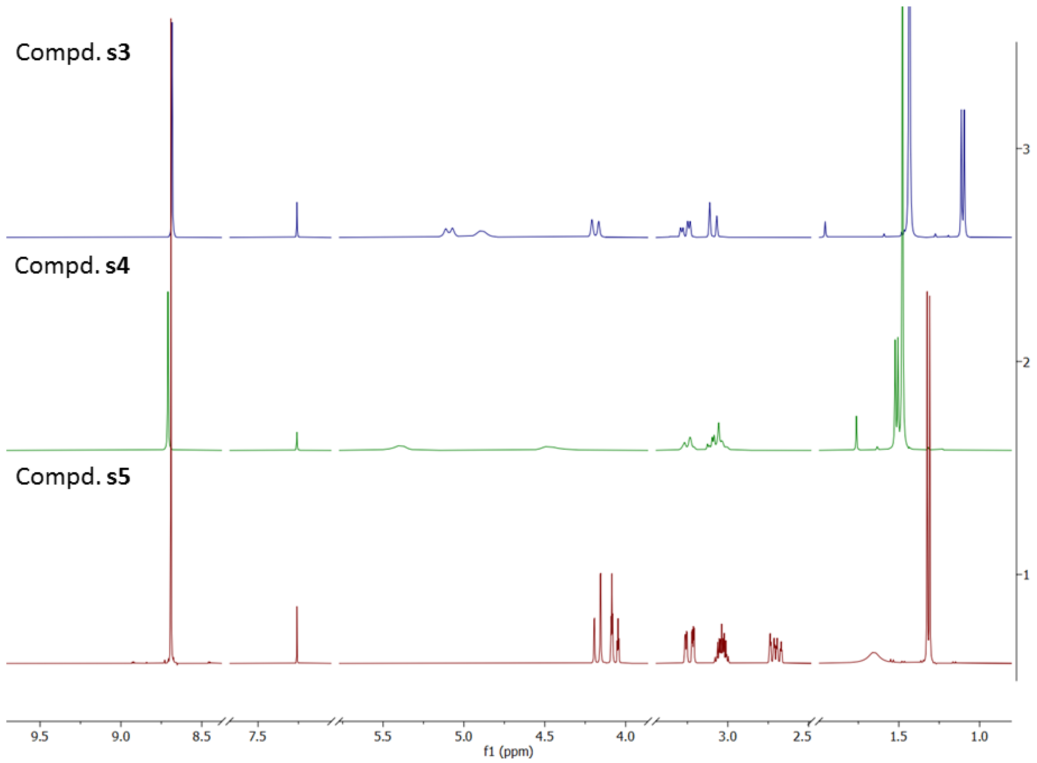

B

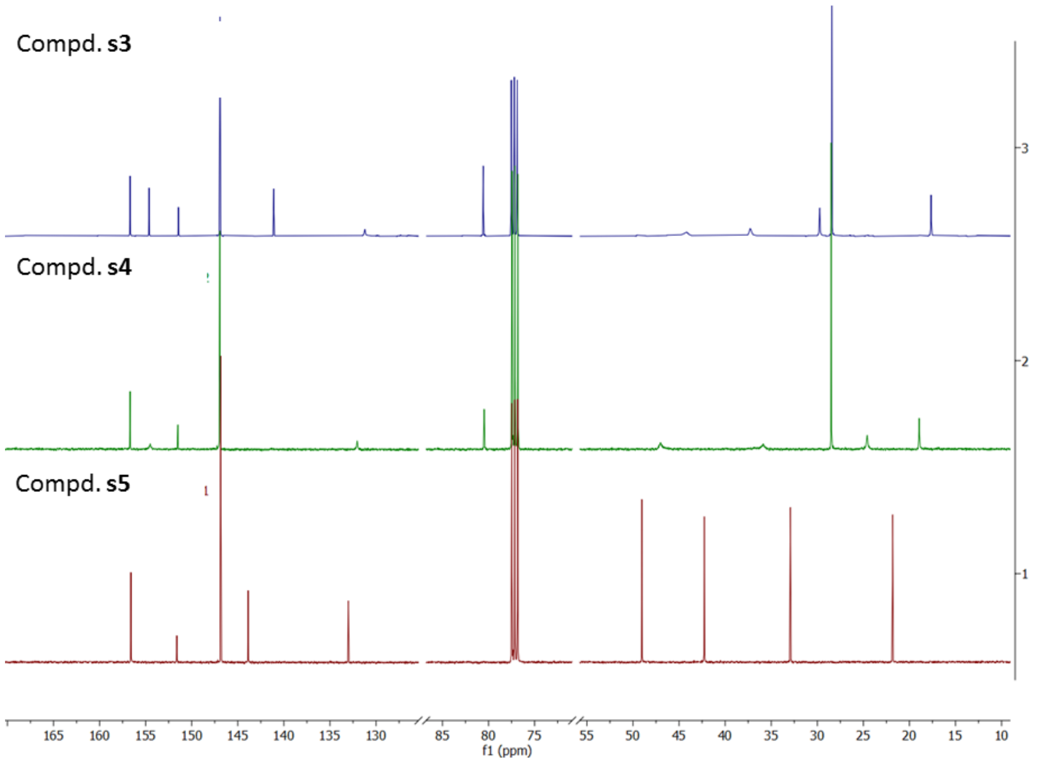

Figure S1. ^{1}H NMR (A) and ^{13}C NMR (B) spectra of the Boc-triazoles **s3** and **s4** amine **s5** in $CDCl_3$.

Table S2. The ^{13}C, ^{1}H and ^{19}F NMR characteristics of compound **JNJ-55308942** in DMSO-d_6 at 25°C if not stated otherwise; the following abbreviations are used: cov – the signal is covered by another; app – apparent; 2nd – the signal of second order; br – broad signal; vbr – very broad signal; the value written in the cursive are rough as it was impossible to read accurate one; other abbreviations are common in NMR description.

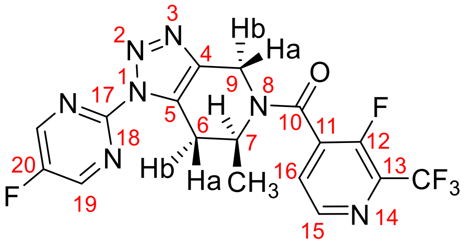

Pos.	Compound **JNJ-55308942**	
	**JNJ-Aa ↔ JNJ-Ab**<br>70%	**JNJ-Ba ↔ JNJ-Bb**<br>30%
4	138.64 s	138.93 br s
5	130.67 vbr s	130.96 s
6	29.44 vbr s<br>H6a 3.058 vbr s<br>H6b 3.252 vbr s, cov	28.39 s<br>H6a 3.234 vbr d, 17.4, cov<br>H6b 3.303 vbr dd, 17.4, 5.6, cov
7-CH_3	17.98 vbs<br>1.159 d, 6.8	16.90 s<br>1.234 d, 7.0
7	48.60 s<br>4.260 vbr s	42.69 s<br>5.389 app kv, 6.5
9	35.12<br>H9a 4.382 br d, 16.5<br>H9b 5.501 br d, 16.5	39.53 cov<br>H9a 4.569 dt, 16.0, 1.6<br>H9b 4.662 d, 16.0
10	161.39	161.62
11	127.41 vbr s	not recognized
12	*152.34* vbr d, *266*<br>^{19}F at 25°C *-129.12* vbr s and *-128.87* vbr s<br>^{19}F at 125°C -128.24 br q, 17.1	*152.16* vbr d, *269*<br>^{19}F at 25°C *-128.56* vbr s
13	134.43 br q, 16.3	*134.13* br q, 16.3
13-CF_3	^{13}C not recognized<br>^{19}F at 25°C -63.95 br d, 15.9<br>^{19}F at 125°C -64.04 d, 17.1	^{13}C not recognized<br>^{19}F at 25°C -64.00 d, 15.8
14	- - -	- - -
15	146.45 d, 5.7<br>8.719 br d, 4.7	146.24 d, 5.8<br>8.693 br d, 4.7
16	*127.38* vbr, cov<br>8.032 br t, 4.7	*127.38* vbr, cov<br>7.945 vbr s
17	150.67 d, 3.1	150.70 d, 3.1
19	147.55 d, 23.1<br>9.106 d, 0.6	147.59 d, 23.1<br>9.128 d, 0.6
20	156.87 d, 261.3<br>^{19}F at 25°C -138.45 vbr s and -138.33 vbr s<br>^{19}F at 125°C -138.52 s	156.88 d, 261.3<br>^{19}F at 25°C -138.31 s

Table S3. The ^{13}C, ^{1}H and ^{19}F NMR characteristics of **AM-10** in DMSO-d_6 at 25°C if not stated otherwise; see also the caption of the Table S1.

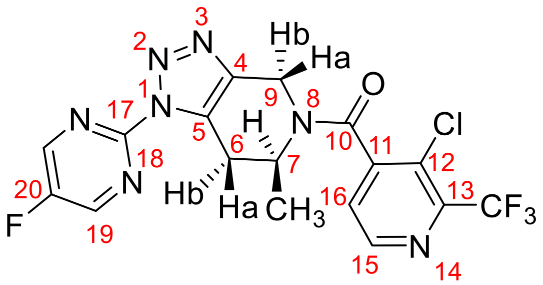

Pos.	Compound **AM-10**			
	**AM-10-Ab**<br>40%	**AM-10-Aa**<br>34%	**AM-10-Ba** or **AM-10-Bb**<br>14%	**AM-10-Ba** or **AM-10-Bb**<br>12%
4	138.676	138.735	138.550	139.153
5	130.916	130.448	131.187	130.760
6	29.209<br>H6a 2.983 dd, 17.3, 1.2<br>H6b 3.269 dd, 17.3, *7*, cov	29.809<br>H6a 3.104 dd, 17.5, 1.0<br>H6b 3.256 dd, 17.5, *7*, cov	28.388<br>3.233 dd, cov<br>3.344 dd, cov	28.476<br>3.268, 2nd
7-CH_3	18.175<br>1.185 d, 6.8	17.640<br>1.143 d, 6.8	16.675<br>1.227 d, 7.0	16.733<br>1.245 d, 7.1
7	48.483<br>4.088 app kv, 6.6	48.512<br>4.156 app kv, 6.6	*42.503*<br>5.409 app kv, cov	*42.412*<br>5.401 app kv, cov
9	34.787<br>H9a 4.380 ddd, 16.4, 2.3, 1.4<br>H9b 5.527 dd, 16.3, 1.3	34.966<br>H9a 4.371 ddd, 16.7, 2.0, 1.2<br>H9b 5.518 dd, 16.6, 1.3	38.695<br>4.514, 2nd<br>4.507, 2nd	39.673<br>4.492 dt, *16.3*, *1.6*, cov<br>4.462 d, 16.3
10	163.223	163.347	163.659	163.412
11	146.727	146.721	146.349	146.303
12	125.473	125.235	125.156	125.391
13	143.637 q, 33.5	143.715 q, 33.5	*143.6*	*143.6*
13-CF_3	120.951 q, 275.4<br>-64.513	120.924 q, 275.4<br>-64.534	*120.93* q<br>-64.550	*120.93* q<br>-64.509
14	- - -	- - -	- - -	- - -
15	148.276<br>8.785 d, 4.8	148.202<br>8.823 d, 4.8	*148.26*<br>8.754 d, 4.8	148.083<br>8.813 d, 4.8
16	125.661<br>7.991 dd, 4.8, 0.4	125.722<br>7.997 dd, 4.8, 0.4	126.008<br>7.803 dd, 4.8, 0.4	125.748<br>7.950 dd, 4.8, 0.4
17	*150.7*	*150.7*	*150.7*	*150.7*
19	147.589 d, 23.1<br>9.111 br d, 0.6	147.540 d, 23.1<br>9.105 br d, 0.6	147.611 d, 23.1<br>9.137 d, 0.6	147.563 d, 23.1<br>9.127 d, 0.6
20	-138.306<br>*156.9* d, *261.3*	-138.413<br>*156.9* d, *261.3*	-138.279<br>*156.9* d, *261.3*	-138.320<br>*156.9* d, *261.3*

Table S4. The ^{13}C, ^{1}H and ^{19}F NMR characteristics of compound **AM-12** in DMSO-d_6 at 25°C if not stated otherwise; see also the caption of the Table S1.

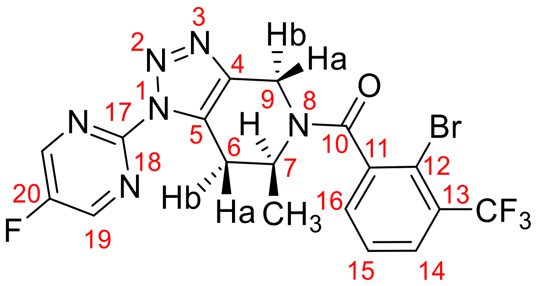

Pos.	Compound **AM-12**			
	**AM-12-Ab**<br>36%	**AM-12-Aa**<br>35%	**AM-12-Ba** or **AM-12-Bb**<br>15%	**AM-12-Ba** or **AM-12-Bb**<br>14%
4	138.976	139.074	138.597	139.285
5	130.912	130.384	*130.36* cov	*130.91* cov
6	29.205<br>H6a 3.003 dt, 17.2, 1.2<br>H6b 3.212 ddt, 17.2, 6.0, 1.8	29.645<br>H6a 3.124 dt, 17.4, 1.1<br>H6b 3.333 ddt, *16.7*, *8.0*, cov	28.494<br>H6a 3.229 dt, 17.5, 1.3, cov<br>H6b 3.336 cov	28.407<br>*3.27*, *2nd*
7-CH_3	18.001<br>1.215 d, 6.8	17.623<br>1.124 d, 6.8	16.427<br>1.235 d, 7.0	16.842<br>1.237 d, 7.0
7	48.567<br>3.980 app kv	48.601<br>4.036 app kv	42.293<br>*5.438* app kv, cov	42.148<br>*5.423* app kv, cov
9	34.647<br>H9a 4.328 ddd, 16.3, 2.2, 1.4<br>H9b 5.552 dd, 16.3, 1.2	34.826<br>H9a 4.338 ddd, 16.6, 2.2, 1.4<br>H9b 5.541 dd, 16.6, 1.3	38.825<br>H9a 4.517 dt, 15.7, 1.8<br>H9b 4.274 dd, 15.7, 1.2	*39.80*<br>H9a 4.468 dt, 16.1, 1.8<br>H9b 4.203 d, 16.1
10	166.175	166.334	166.539	166.280
11	141.245	141.265	140.982	140.880
12	*116.16*	*115.88*	*115.9*	*115.9*
13	*129.4* q, *30*	*129.4* q, *30*	*129.4* q, *30*	*129.4* q, *30*
13-CF_3	*122.9* q, *274*<br>-60.88 or -60.92	*122.9* q, *274*<br>-60.88 or -60.92	*122.9* q, *274*<br>-60.87 or -60.97	*122.9* q, *274*<br>-60.87 or -60.97
14	*128.41* q, *5.7*<br>7.925 dd, 7.9, 1.7	*128.41* q, *5.7*<br>7.933 dd, 7.9, 1.7	*128.07* q, *5.5*<br>*7.878* dd, *7.7*, *1.8*	*128.07* q, *5.5*<br>*7.878* dd, *7.7*, *1.8*
15	129.054<br>7.688 cov	129.119<br>7.736 cov	*128.91*<br>*7.67* cov	*128.91*<br>*7.67* cov
16	131.336<br>7.790 dd, cov	131.427<br>7.802 dd, cov	*131.72* cov<br>*7.63* cov	*131.72* cov<br>*7.63* cov
17	*150.67*	*150.67*	*150.67*	*150.67*
19	*147.57* d, *23.1*<br>*105.10* d, 0.6	*147.57* d, *23.1*<br>*105.10* d, 0.6	*147.57* d, *23.1*<br>9.136 d, 0.6	*147.57* d, *23.1*<br>9.127 d, 0.6
20	-138.333 or -138.374<br>*156.85* d, *261.3*	-138.333 or -138.374<br>*156.85* d, *261.3*	-138.285 or -138.326<br>*156.87* d, *261.3*	-138.285 or -138.326<br>*156.87* d, *261.3*

Table S5. The ^{13}C, ^{1}H and ^{19}F NMR characteristics of compound **AM-15** in DMSO-d_6 at 25°C if not stated otherwise; see also the caption of the Table S1.

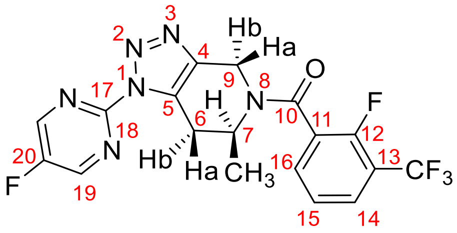

Pos.	Compound **AM-15**	
	**AM-15-Aa ↔ AM-15-Ab**<br>71%	**AM-15-Ba ↔ AM-15-Bb**<br>29%
4	*138.95*	*138.95*
5	130.76 vbr and 130.49 vbr	131.16 br
6	29.61 vbr and 29.61 vbr<br>3.05 vbr d, *17*<br>3.18 vbr d, *17*	28.45<br>H6a 3.22 br d, *17.5*<br>H6b 3.30 br dd, *17.5*, *5.6*
7-CH_3	17.97 vbr s and 17.83 vbr s<br>1.154 d, 6.8	16.93 sharp<br>1.231 d, 7.0
7	48.75<br>4.189 vbr s	42.58<br>5.409 app kv, 6.6
9	*35.08* br<br>H9a 4.347 d, 16.5<br>H9b 5.507 d, 16.5	*39.57* br, cov<br>H9a 4.590 d, 15.9<br>H9b 4.463 d, 15.9
10	163.30	163.41 br
11	*125.77* br, cov	not recognized
12	*154.72* vbr d, *256*<br>^{19}F at 20.6°C -*120.4* br s and -*120.5* br s<br>^{19}F at 148°C -119.3 m	^{13}C not recognized<br>^{19}F at 20.6°C -*119.9* vbr s and -*120.1* vbr s
13	*117.23* br qd, *32*, *11*	not recognized
13-CF_3	122.41 q, 272.4<br>^{19}F at 20.6°C -*59.8* br d, cov<br>^{19}F at 148°C -59.6 d, 14.7, sat dd 272.7, 14.7	not recognized<br>^{19}F at 20.6°C -*59.9* vbr, cov
14	*128.53* br, cov<br>^{1}H at 25°C 7.91 br t, cov<br>^{1}H at 125°C 7.86 br ddd, 7.8, 7.2, 2.1	*128.53* br, cov<br>^{1}H at 25°C 7.91 br t, cov<br>^{1}H at 125°C 7.86 br ddd, 7.8, 7.2, 2.1
15	*125.84* br cov<br>^{1}H at 20.6°C 7.54 br, cov<br>^{1}H at 125°C 7.53 t, 7.8	*125.84* br cov<br>^{1}H at 25°C 7.54 br, cov<br>^{1}H at 125°C 7.53 t, 7.8
16	*133.33* two br signals, cov<br>^{1}H at 25°C 7.89 br t, cov<br>^{1}H at 125°C 7.79 br ddd, 7.8, 6.2, 1.9	*133.33* br cov<br>^{1}H at 25°C 7.82 vbr<br>^{1}H at 125°C 7.79 br ddd, 7.8, 6.2, 1.9
17	150.71 d, 3.2	not recognized
19	147.53 d, 23.1<br>9.093 s, sat dd 191.1, 3.4	147.57 d, 23.1<br>9.117 s
20	156.89 d, 261.4<br>^{19}F at 20.6°C -138.4 vbr s and -138.5 vbr s<br>^{19}F at 148°C -138.6 s, sat d 262.6	^{13}C not recognized<br>^{19}F at 20.6 °C -138.4 s

Table S6. 3D geometry of **JNJ-55308942**

N	-0.10540	-0.42680	-0.66080
N	1.14060	-0.98470	-0.53970
N	1.22960	-1.47550	0.66300
C	0.05940	-1.24760	1.32650
C	-0.26550	-1.66560	2.72490
N	-1.70660	-1.44950	2.93920
C	-2.27890	-0.17690	2.43610
C	-2.17680	-0.12560	0.89280
C	-0.81000	-0.57710	0.50280
C	-0.52610	0.14490	-1.88780
N	0.42990	0.46830	-2.75790
C	0.01150	0.99100	-3.91420
C	-1.34220	1.19250	-4.15710
C	-2.25760	0.83070	-3.17740
N	-1.84290	0.28850	-2.02820
C	-2.36490	-2.33460	3.72840
O	-1.81400	-3.26860	4.31790
C	-1.60840	1.02460	3.11510
F	-1.75410	1.72610	-5.31410
C	-3.86130	-2.18600	3.88560
C	-4.75150	-2.30910	2.82400
C	-6.12760	-2.28160	3.04360
N	-6.65220	-2.13630	4.26080
C	-5.80840	-2.02620	5.29520
C	-4.42130	-2.05930	5.15870
F	-4.28190	-2.44660	1.56800
C	-7.07590	-2.40570	1.86560
F	-6.82850	-3.53150	1.15780
F	-8.35830	-2.43880	2.25410
F	-6.92950	-1.36450	1.01350
H	0.32210	-1.10260	3.46130
H	-0.05350	-2.72400	2.88190
H	-3.33380	-0.16570	2.70500
H	-2.37810	0.88840	0.53890
H	-2.93200	-0.77570	0.44410
H	0.75620	1.26300	-4.65680
H	-3.32430	0.97170	-3.32490
H	-0.55770	1.12050	2.82690
H	-2.12330	1.94320	2.82050
H	-1.66570	0.92540	4.20250
H	-6.25920	-1.90970	6.27650
H	-3.77770	-1.98140	6.02760

Table S7. 3D geometry of **AM-10-Ab**

N	0.04170	-0.26900	-0.86390
N	0.65250	0.68800	-0.09590
N	0.22190	0.53870	1.12350
C	-0.65990	-0.50190	1.16170
C	-1.39480	-1.00470	2.36420
N	-2.06400	-2.26420	1.99820
C	-2.74230	-2.31890	0.68200
C	-1.70040	-2.16380	-0.45220
C	-0.79160	-1.03570	-0.09610
C	0.32830	-0.37910	-2.24810
N	0.02780	-1.55320	-2.80270
C	0.27360	-1.66730	-4.11170
C	0.83420	-0.61100	-4.81820
C	1.12390	0.56800	-4.14040
N	0.85760	0.69260	-2.83690
C	-2.02800	-3.29120	2.88030
O	-1.51150	-3.23330	3.99860
C	-3.86610	-1.27920	0.60540
F	1.09040	-0.72460	-6.12760
C	-2.61890	-4.61400	2.43820
C	-3.94220	-4.97380	2.71130
C	-4.37980	-6.26060	2.36670
N	-3.58140	-7.16130	1.79320
C	-2.31490	-6.81820	1.53570
C	-1.79140	-5.56450	1.83910
Cl	-5.00760	-3.79560	3.45390
C	-5.81030	-6.71810	2.62240
F	-6.01120	-7.97790	2.21020
F	-6.11340	-6.66630	3.93900
F	-6.69960	-5.93180	1.97470
H	-2.13090	-0.27200	2.71740
H	-0.71560	-1.21260	3.19210
H	-3.18510	-3.30940	0.59010
H	-2.20620	-1.98710	-1.40430
H	-1.12580	-3.08930	-0.56480
H	0.03240	-2.60600	-4.60220
H	1.56320	1.41520	-4.65980
H	-3.47540	-0.25760	0.61690
H	-4.42560	-1.41590	-0.32390
H	-4.55160	-1.40320	1.44750
H	-1.69470	-7.57590	1.06570
H	-0.75840	-5.32560	1.61040

Table S8. 3D geometry of **AM-15-Aa**

N	-0.62660	0.05490	1.09520
N	-0.58820	-0.86920	0.08370
N	-0.17230	-1.99040	0.60020
C	0.05740	-1.80980	1.93310
C	0.52450	-2.84400	2.90870
N	0.34570	-2.29550	4.26240
C	0.79660	-0.90060	4.48060
C	-0.06320	0.07380	3.63830
C	-0.22390	-0.50950	2.27430
C	-1.10090	1.37030	0.85790
N	-1.10120	1.78850	-0.40660
C	-1.56790	3.02460	-0.60950
C	-1.99730	3.80950	0.45490
C	-1.94330	3.28190	1.73770
N	-1.49830	2.03770	1.94030
C	-0.15650	-3.11020	5.22720
O	-0.36580	-4.31520	5.05520
C	2.29700	-0.76390	4.18940
F	-2.45340	5.05110	0.24570
C	-0.46540	-2.50530	6.57500
C	-1.48870	-1.58070	6.75150
C	-1.83230	-1.07680	8.00460
C	-0.10630	-2.46610	8.97570
C	0.21250	-2.95950	7.71240
F	-2.17450	-1.14970	5.66580
C	-2.93520	-0.06180	8.12040
F	-2.66470	1.05380	7.40260
F	-4.11370	-0.54340	7.66010
F	-3.13160	0.32450	9.39850
H	1.57390	-3.11530	2.73740
H	-0.06540	-3.75780	2.83320
H	0.63800	-0.67170	5.53310
H	0.41540	1.05570	3.59750
H	-1.04000	0.21450	4.10670
H	-1.59480	3.40440	-1.62660
H	-2.26760	3.86110	2.59730
H	2.86390	-1.50390	4.76110
H	2.52060	-0.89580	3.12670
H	2.63310	0.23490	4.48110
H	0.43770	-2.81390	9.84710
H	1.00080	-3.69590	7.59180
C	-1.12570	-1.52460	9.12210
H	-1.38040	-1.13570	10.1009<br>0

Table S9. 3D geometry of **AM-12-Ab**

N	3.54160	-1.11820	-0.17290
N	4.01790	-2.31060	-0.65640
N	3.01180	-3.13420	-0.71380
C	1.88650	-2.49690	-0.27720
C	0.51410	-3.08220	-0.17350
N	-0.42950	-2.00070	0.14880
C	-0.00240	-1.00540	1.15850
C	1.22760	-0.22320	0.63450
C	2.19840	-1.20750	0.07440
C	4.40170	-0.00130	-0.02320
N	5.70790	-0.25590	0.04450
C	6.50260	0.81030	0.17260
C	5.97060	2.09310	0.25160
C	4.59160	2.24740	0.18470
N	3.79530	1.18450	0.03330
C	-1.63170	-1.98780	-0.48640
O	-1.97620	-2.83300	-1.31440
C	0.25690	-1.67040	2.51570
F	6.77190	3.15650	0.39160
C	-2.56830	-0.84130	-0.17440
C	-3.47960	-0.90090	0.88240
C	-4.39350	0.13930	1.10160
C	-3.48790	1.30520	-0.82120
C	-2.58460	0.26720	-1.02870
Br	-3.42440	-2.43490	2.02610
C	-5.37760	0.10230	2.24670
F	-6.20570	-0.96440	2.16560
F	-4.75140	0.02930	3.44410
F	-6.15600	1.20450	2.27550
H	0.47200	-3.86450	0.59540
H	0.19720	-3.53280	-1.11490
H	-0.82080	-0.29660	1.27450
H	1.67140	0.36400	1.44200
H	0.92230	0.48640	-0.14170
H	7.57550	0.64850	0.22400
H	4.13320	3.23010	0.24460
H	0.48250	-0.90320	3.26130
H	1.10460	-2.36040	2.47610
H	-0.63200	-2.21660	2.83590
H	-3.49300	2.16460	-1.48230
H	-1.87870	0.30910	-1.85200
C	-4.38790	1.23970	0.24080
H	-5.09280	2.04410	0.40970

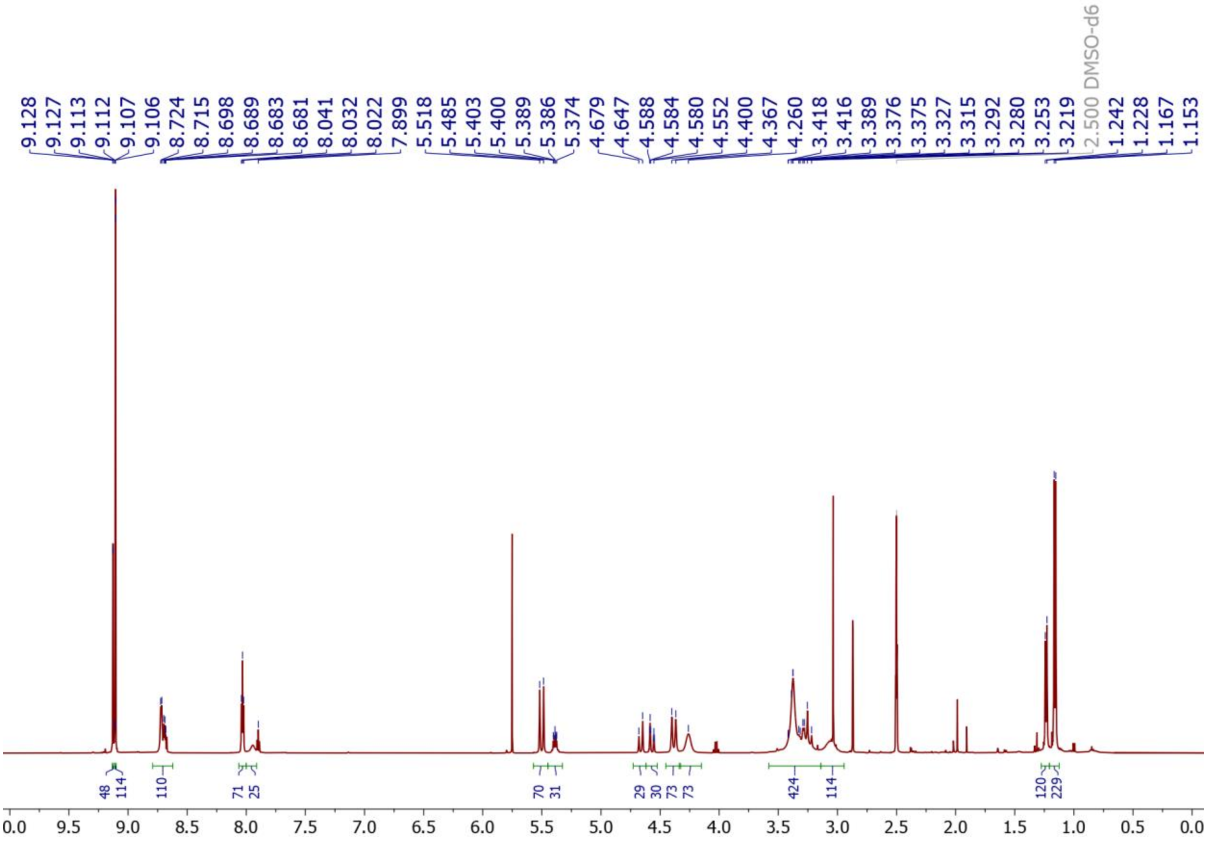

Figure S2. Compound **JNJ-55308942**, ^{1}H NMR (500 MHz, DMSO-d_6, 25°C)

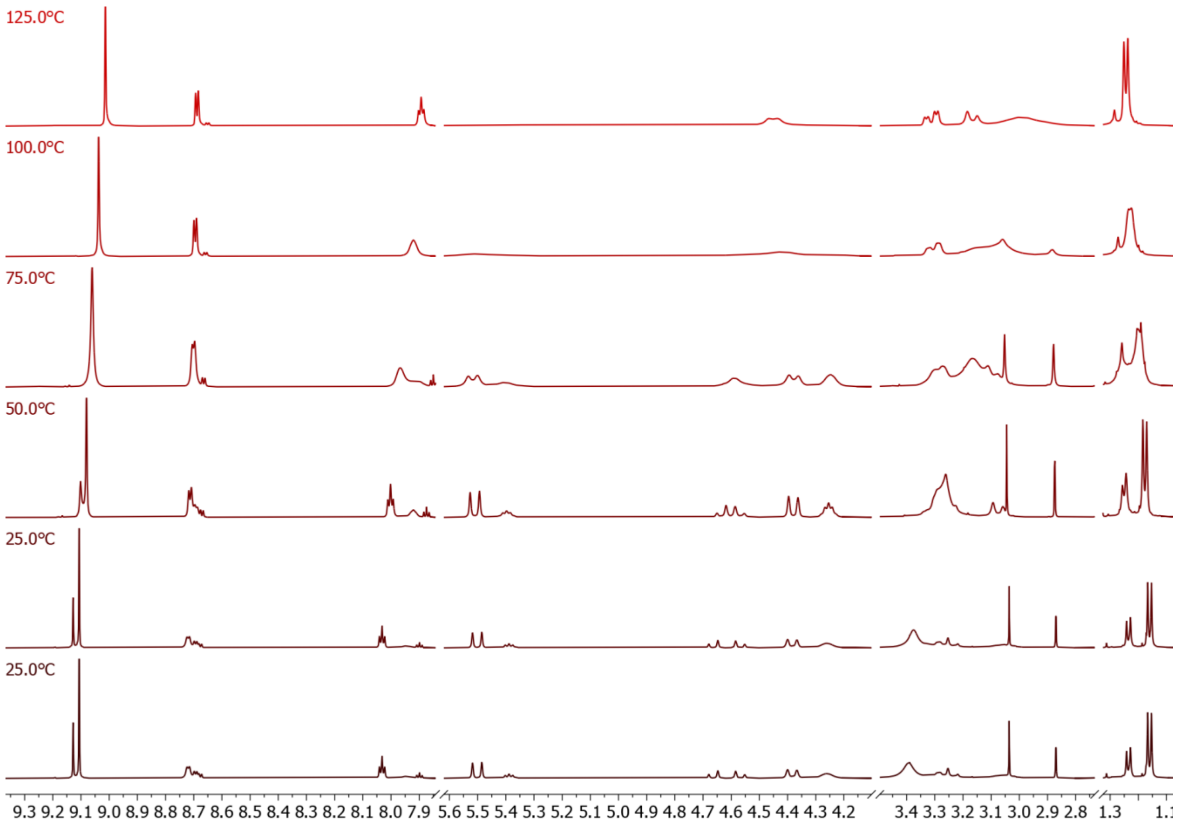

Figure S3. Compound **JNJ-55308942**, ^{1}H NMR (500 MHz, DMSO-d_6) at various temperature

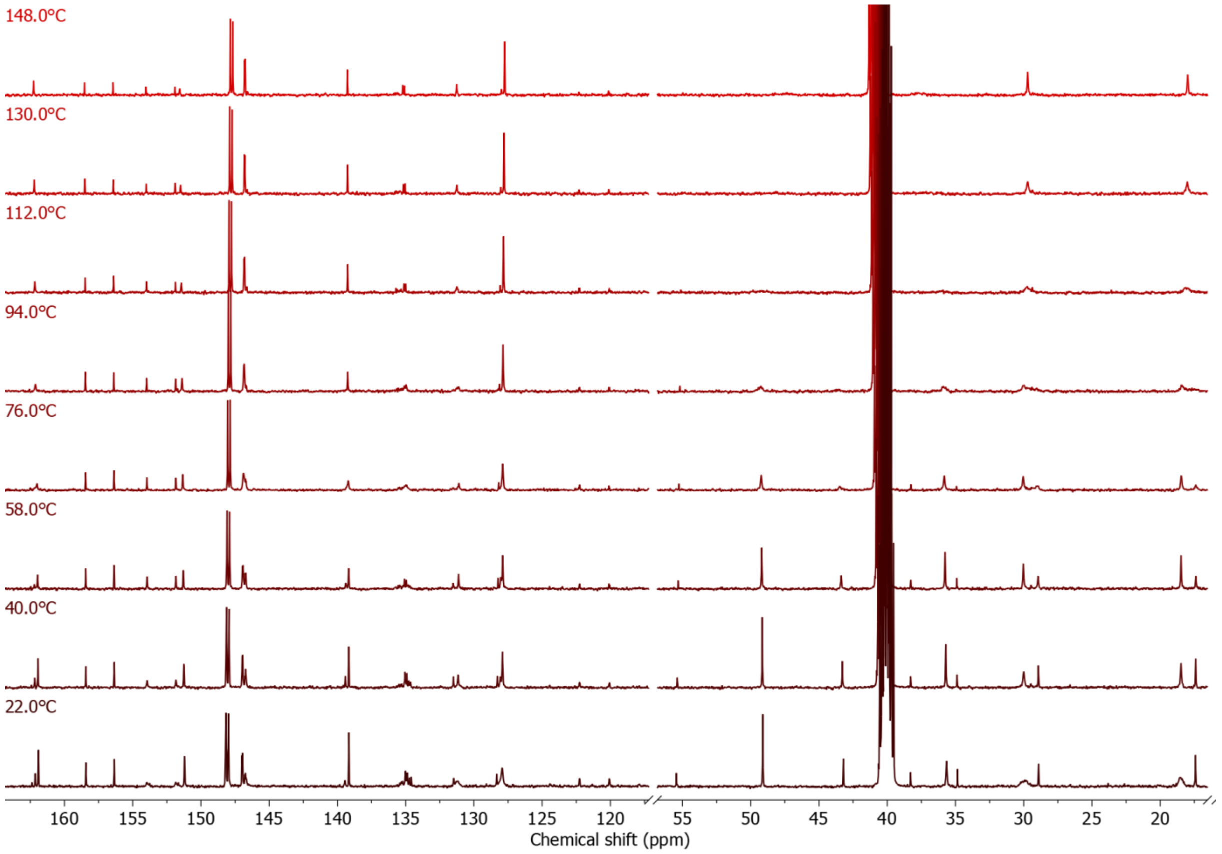

Figure S4. Compound **JNJ-55308942**, ^{13}C NMR (126 MHz, DMSO-d_6) at various temperature

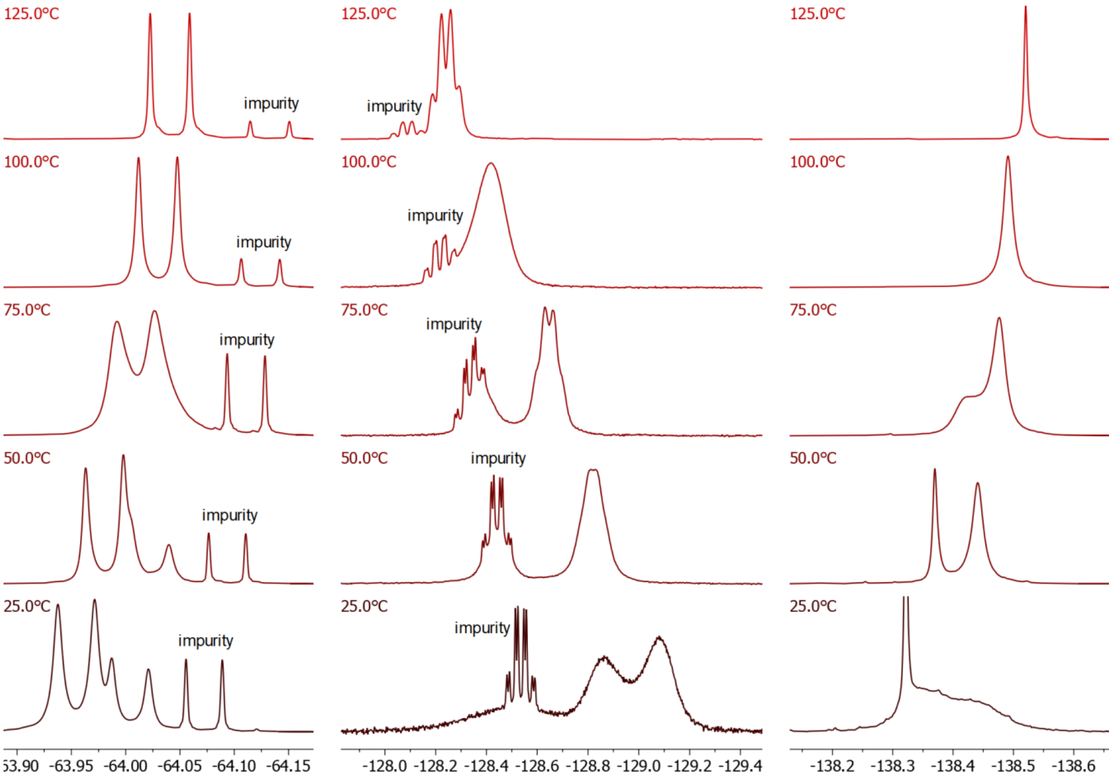

Figure S5. Compound **JNJ-55308942**, ^{19}F NMR (471 MHz, DMSO-d_6) at various temperature

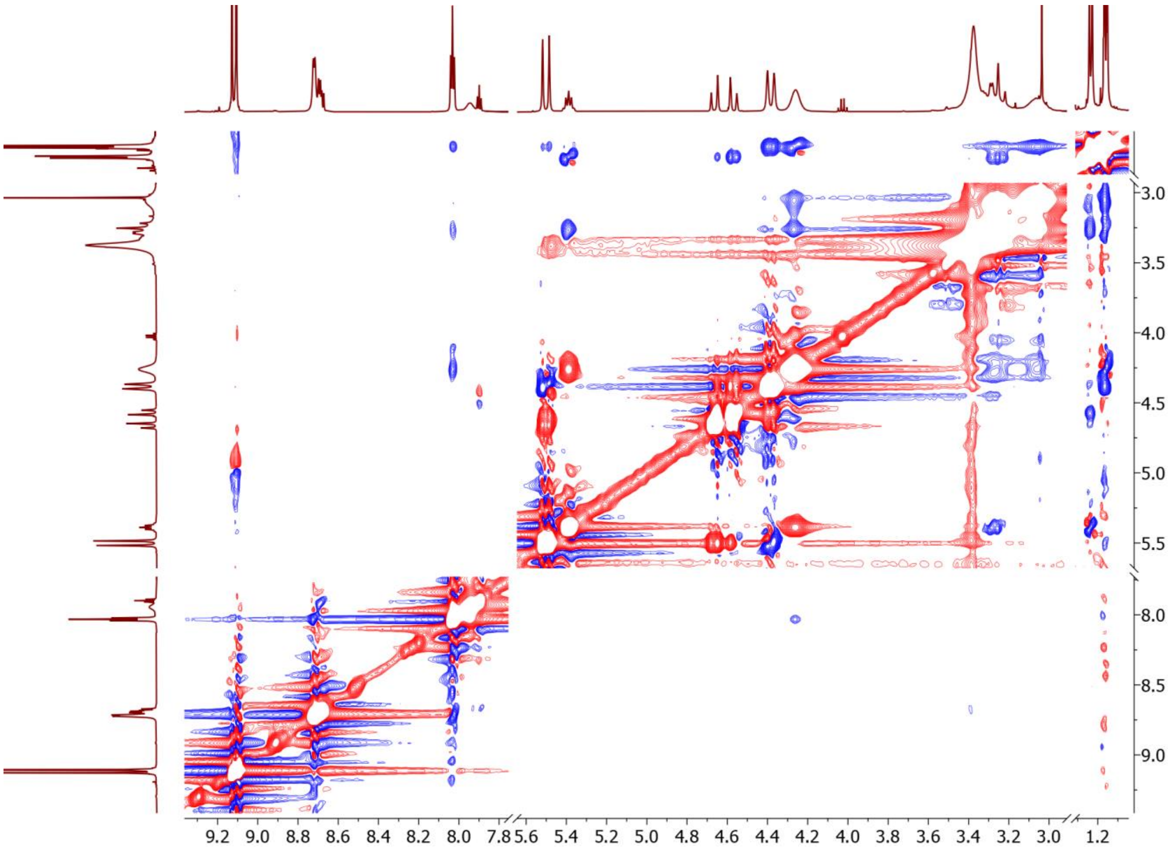

Figure S6. Compound **JNJ-55308942**, ^{1}H-^{1}H NOESY NMR (500 MHz, DMSO-d_6, 25°C, mixing time 80 ms)

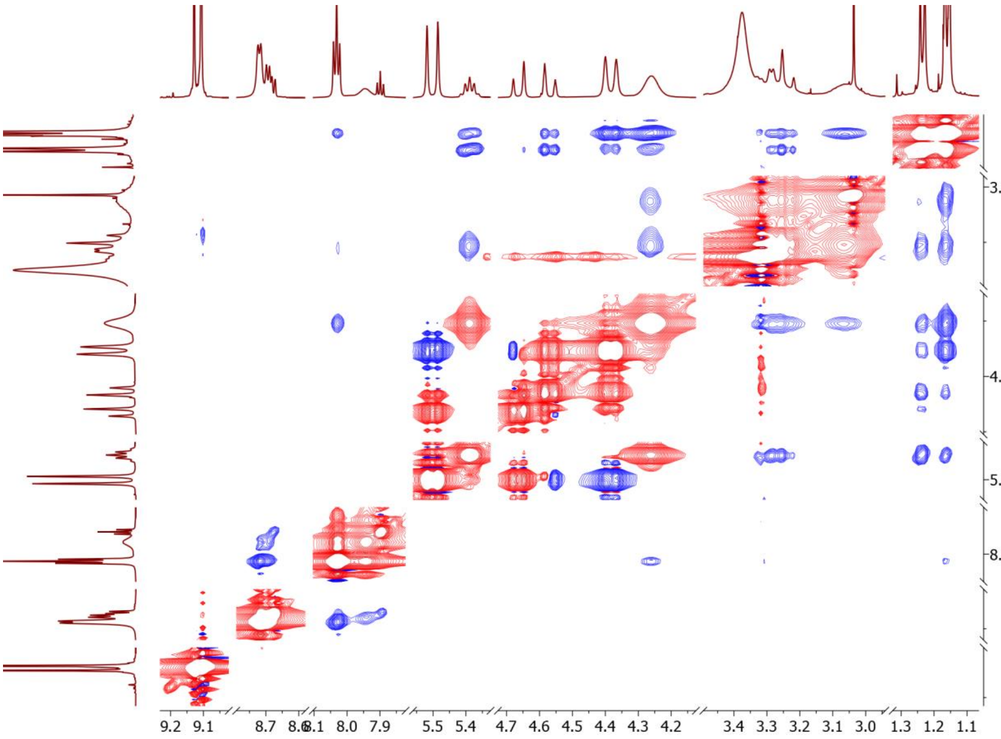

Figure S7. Compound **JNJ-55308942**, ^{1}H-^{1}H NOESY NMR (500 MHz, DMSO-d_6, 25°C, mixing time 450 ms)

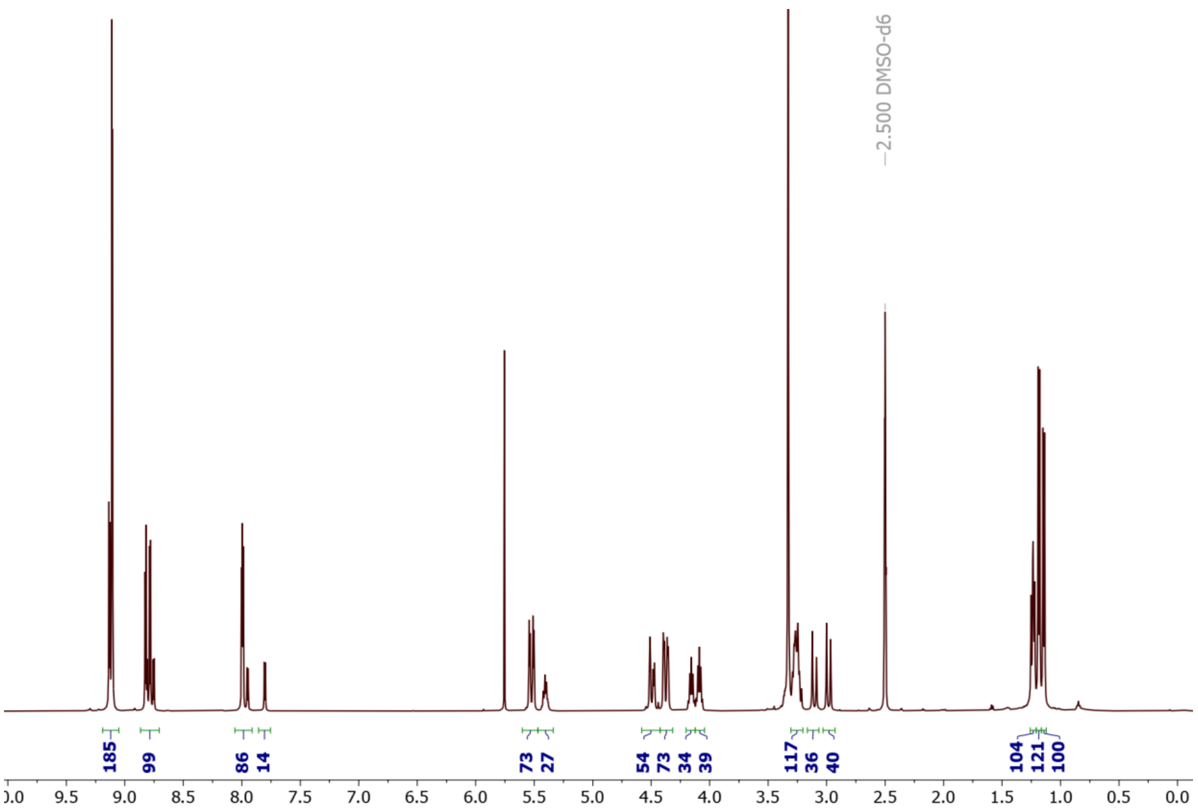

Figure S8. Compound **AM-10**, ^{1}H NMR (500 MHz, DMSO-d_6, 22°C)

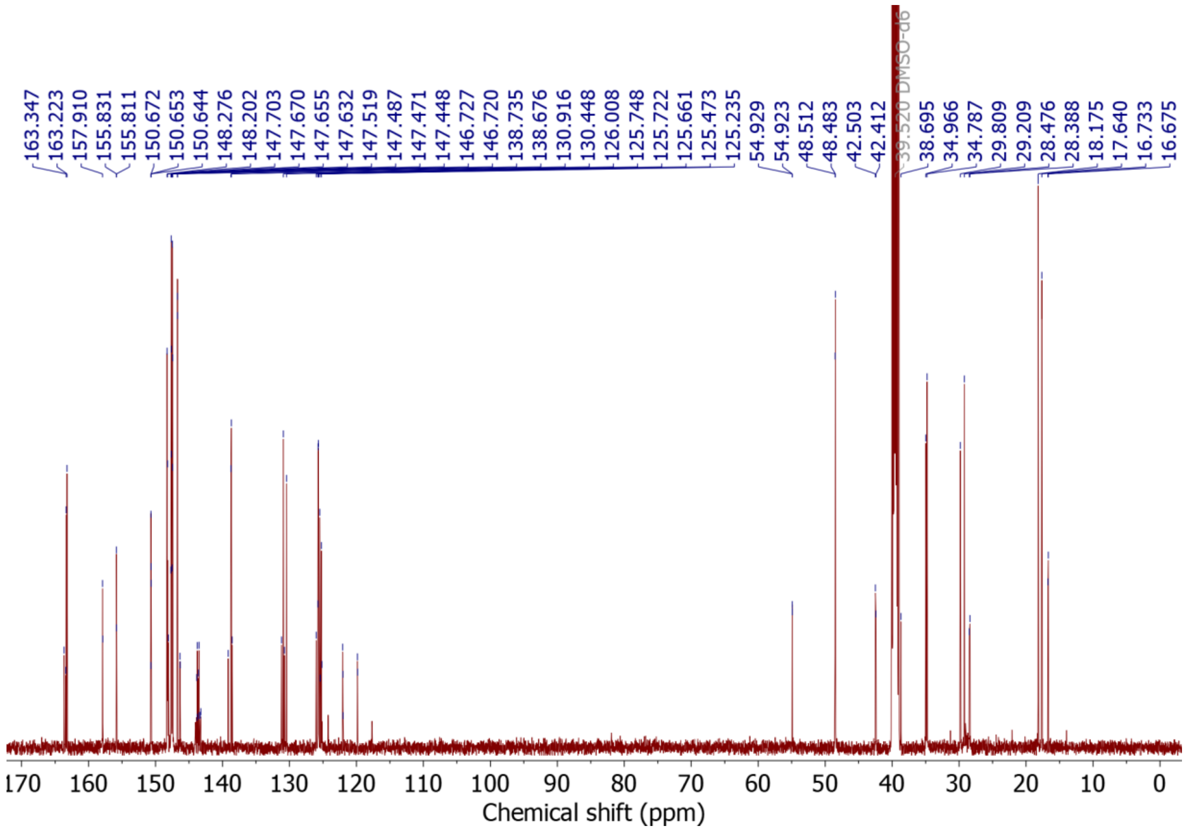

Figure S9. Compound **AM-10**, ^{13}C NMR (126 MHz, DMSO-d_6, 22°C)

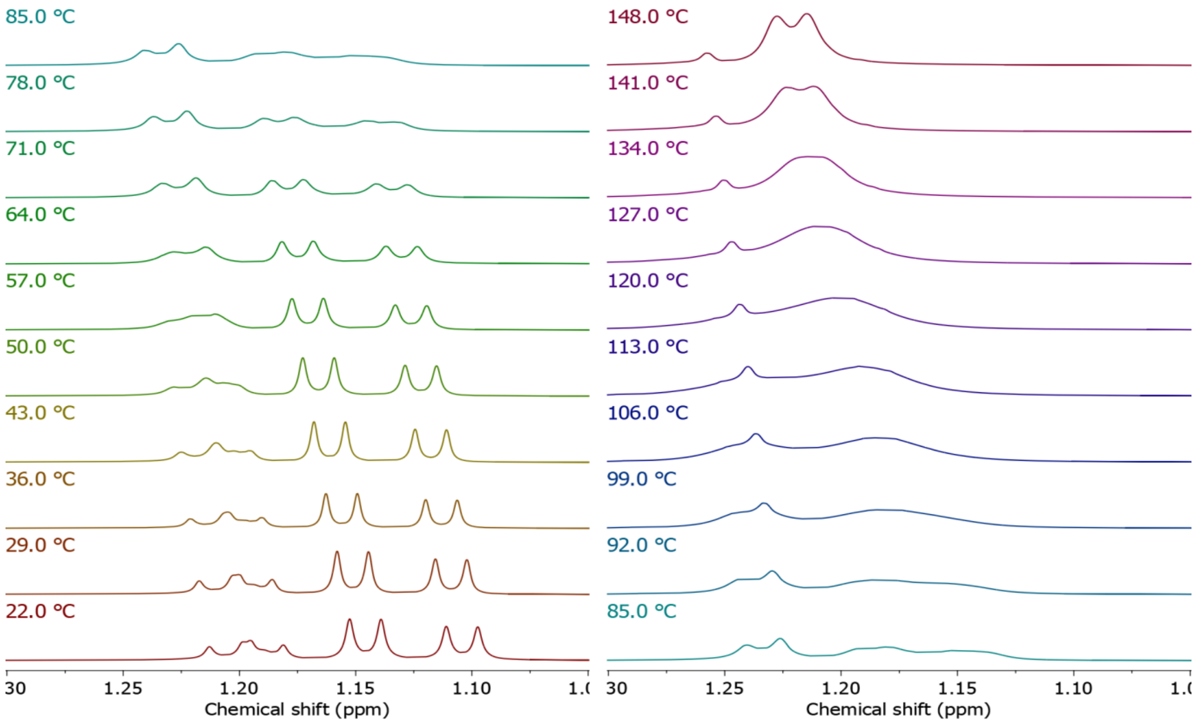

Figure S10. Compound **AM-10**, ^{1}H NMR (500 MHz, DMSO-d_6), the methyl signals at various temperature

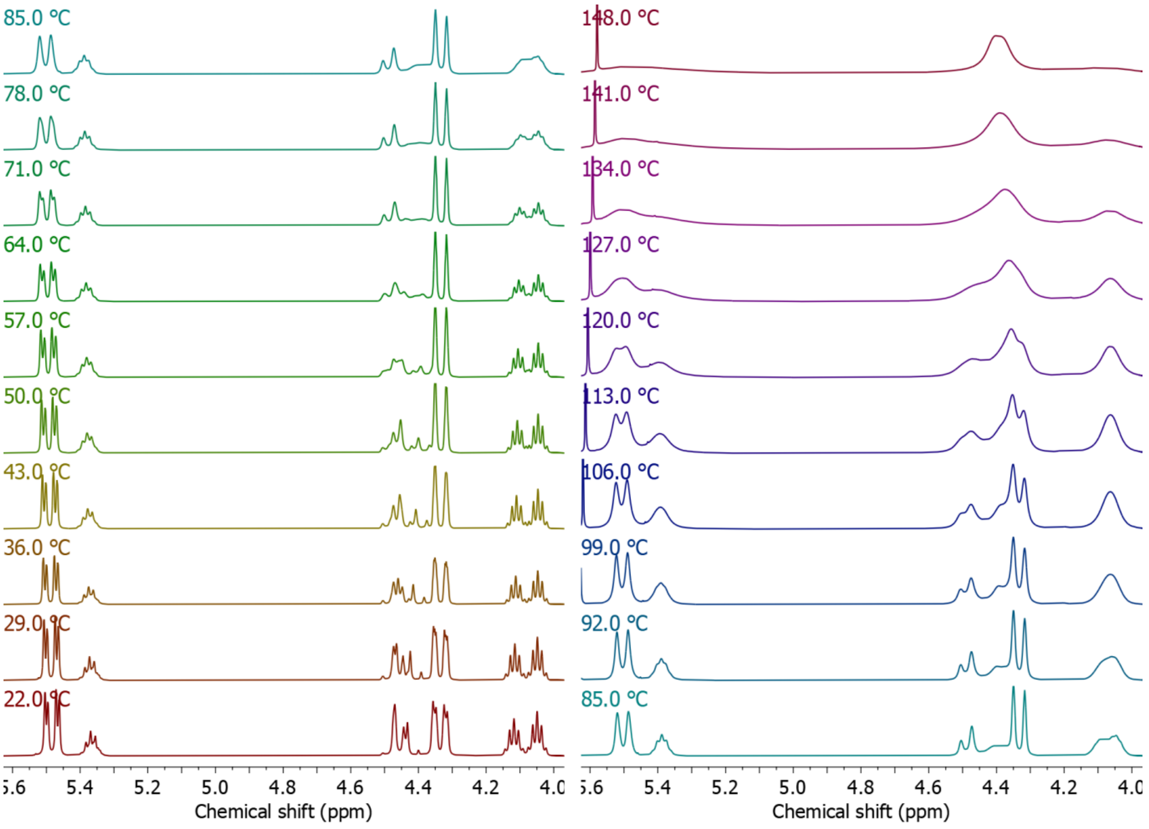

Figure S11. Compound **AM-10**, ^{1}H NMR (500 MHz, DMSO-d_6), the signals H7, H9a and H9b at various temperature

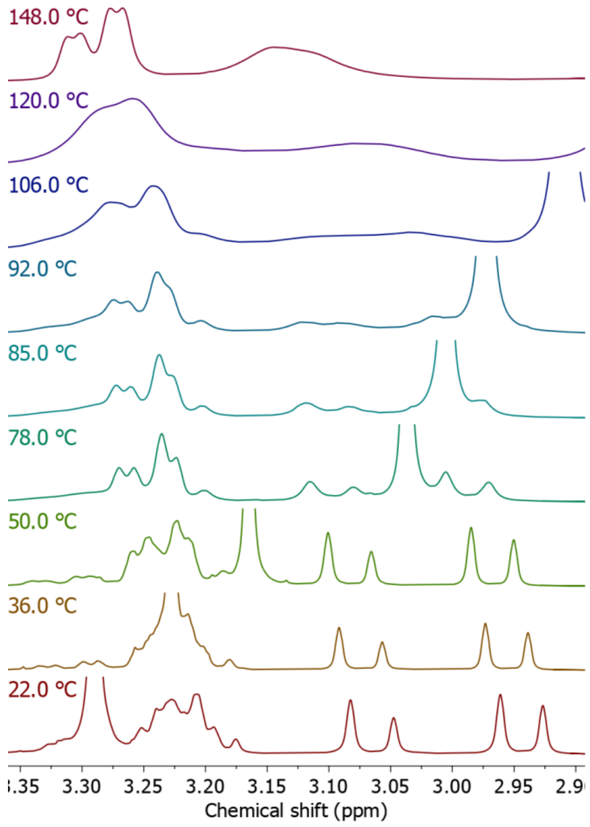

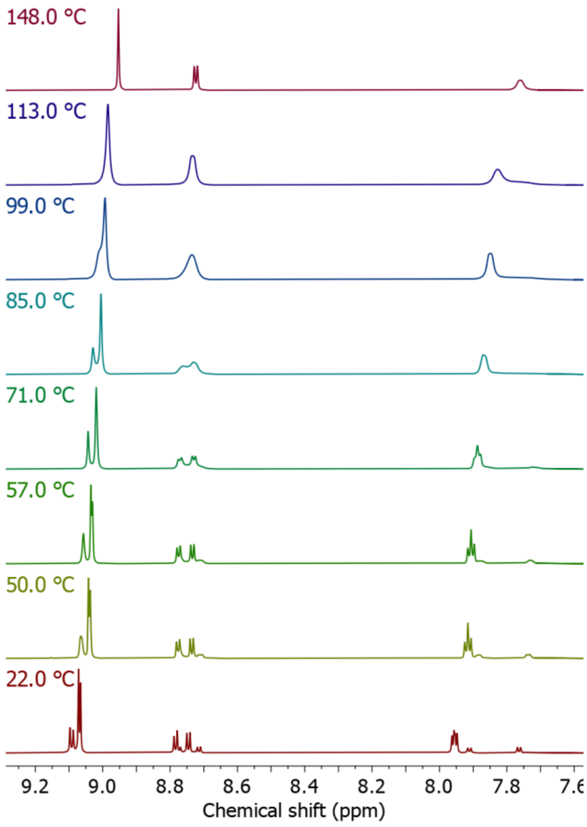

Figure S12. Compound **AM-10**, ^{1}H NMR (500 MHz, DMSO-d_6), H6a, H6b, H19, H16, and H15 at various temperature

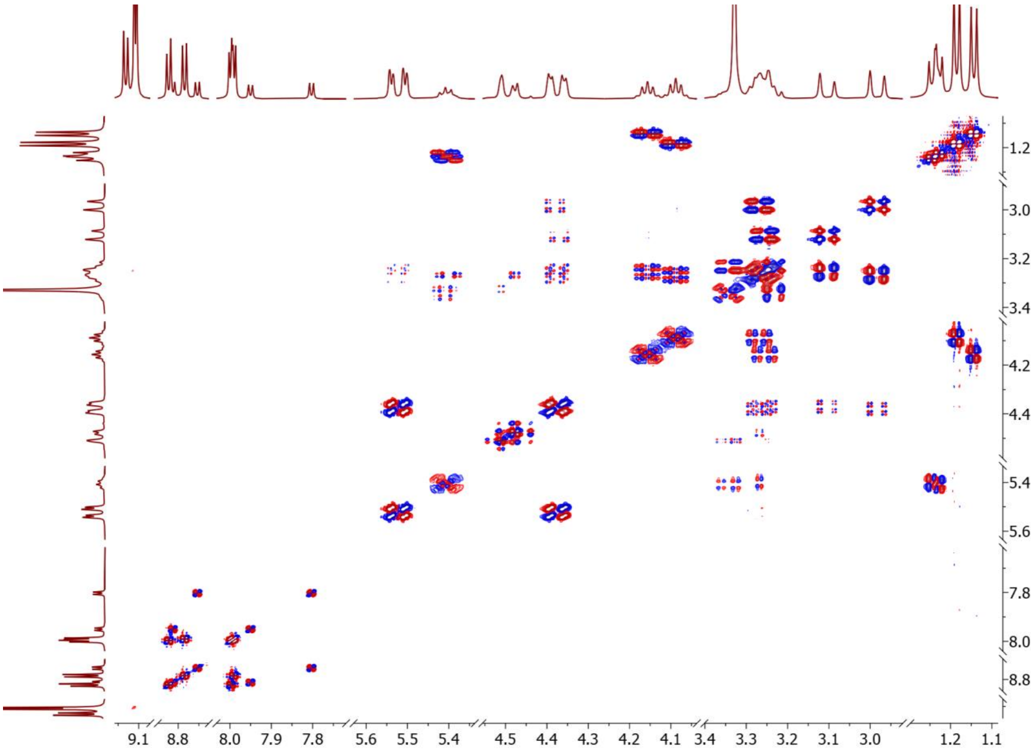

Figure S13. Compound **AM-10**, ^{1}H-^{1}H DQF COSY NMR (500 MHz, DMSO-d_6, 22°C)

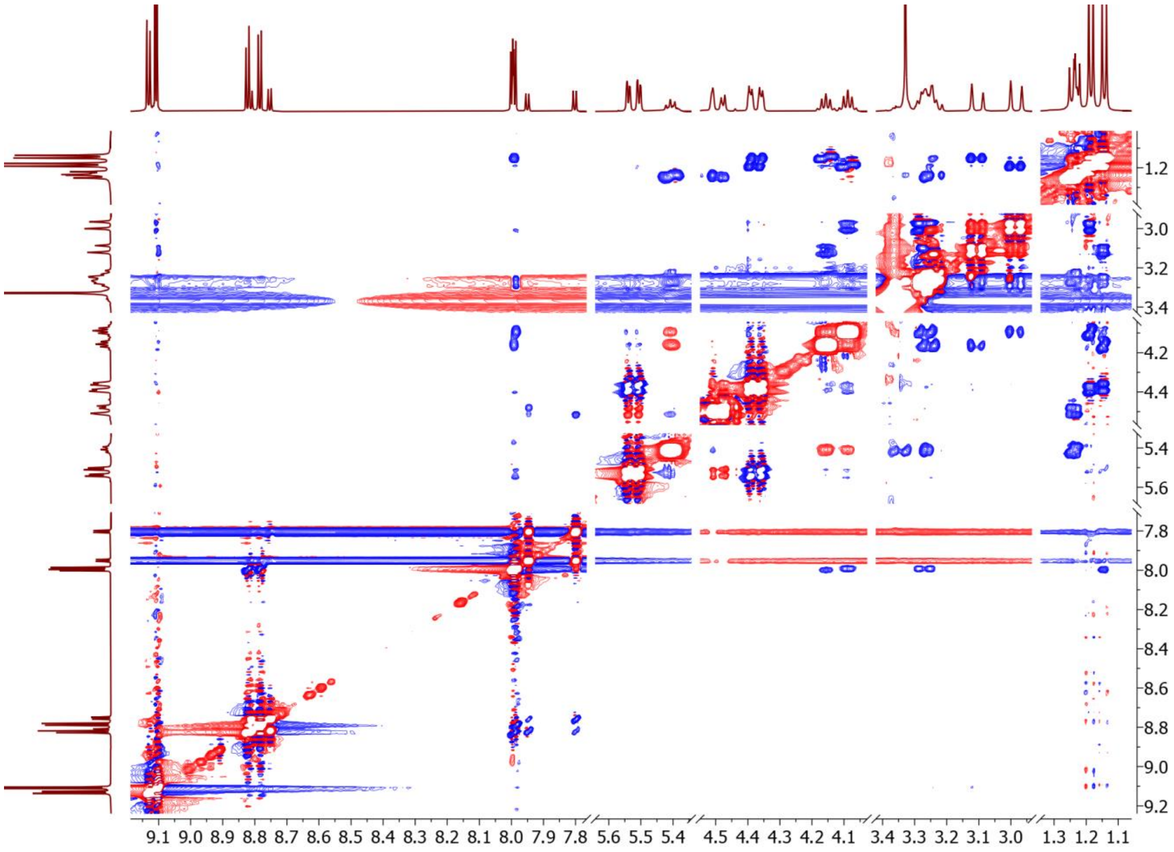

Figure S14. Compound **AM-10**, ^{1}H-^{1}H NOESY NMR (500 MHz, DMSO-d_6, 25°C, mixing time 320 ms)

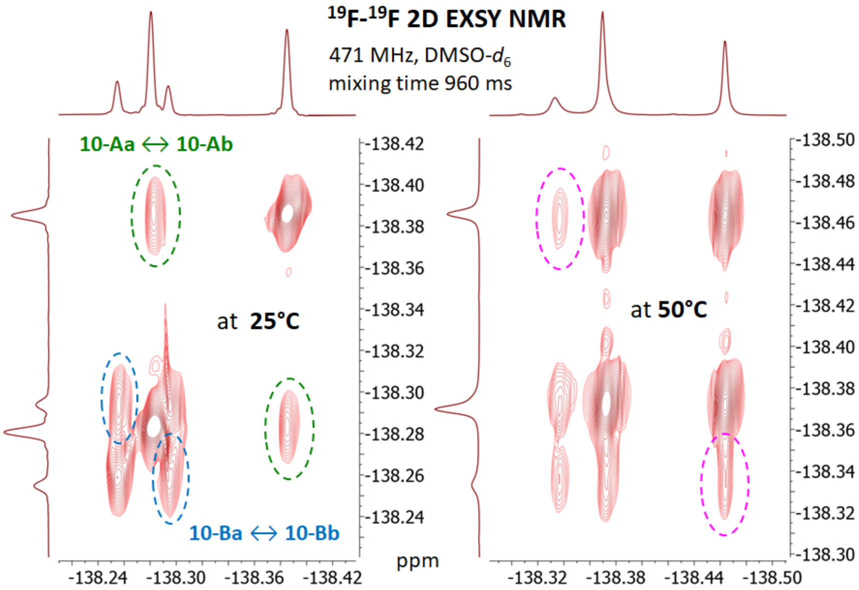

Figure S15. Compound **AM-10**, ^{19}F-^{19}F 2D EXSY NMR spectra at 25°C (only the chemical exchange due to the aryl rotation is observed) and at 50°C (also the chemical exchange due to the carbonyl group rotation is observed); the signals of the fluorine F20 are shown.

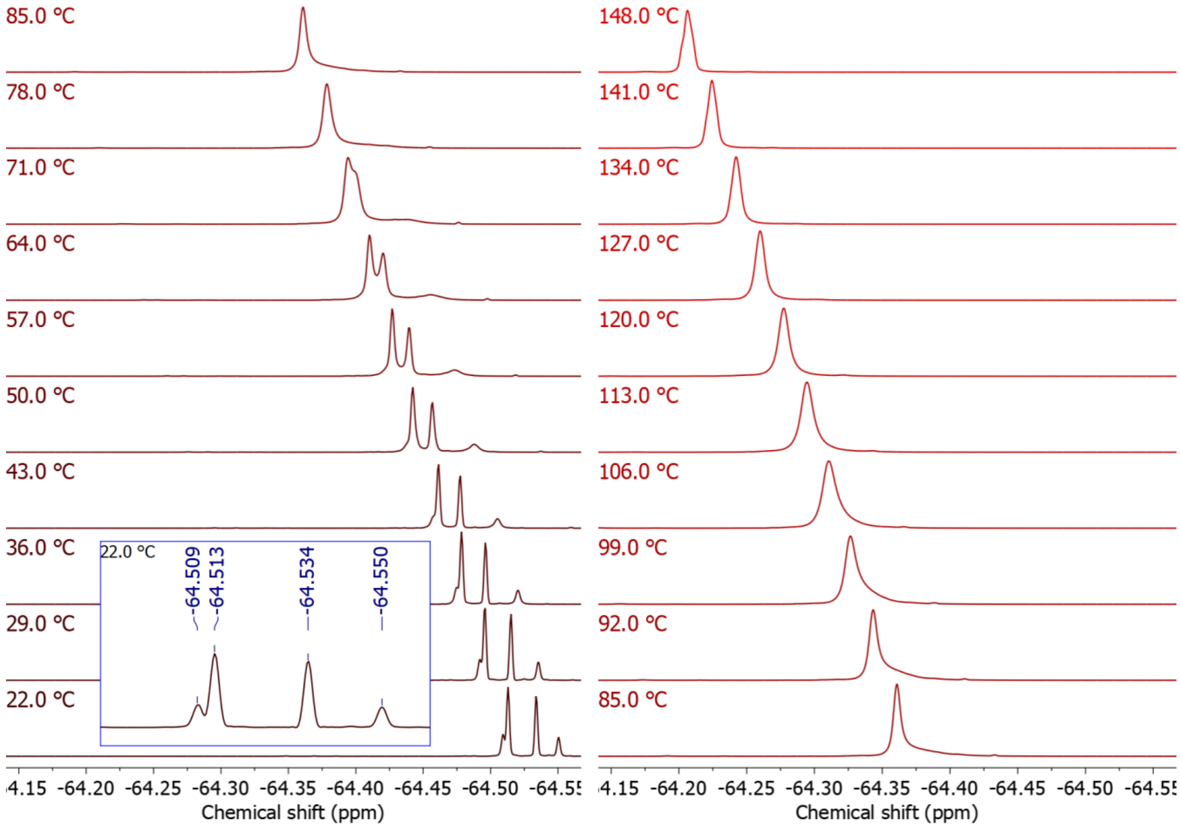

Figure S16. Compound **AM-10**, ^{19}F NMR (471 MHz, DMSO-d_6), the signals of the CF_3 groups at various temperature

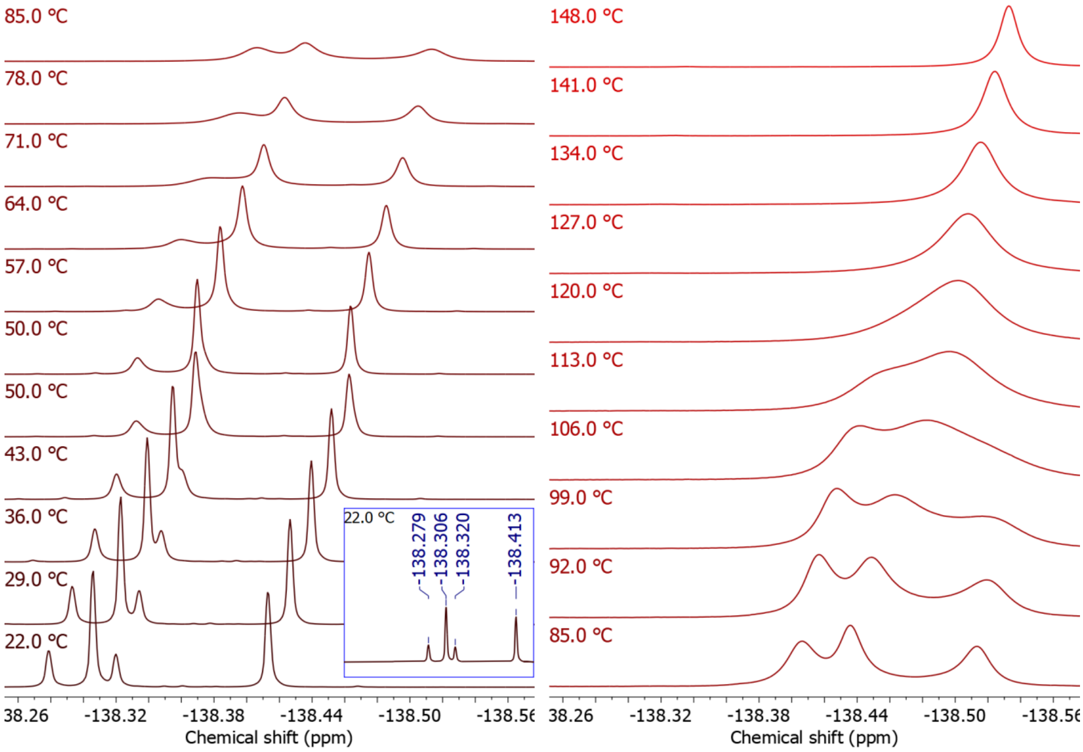

Figure S17. Compound **AM-10**, ^{19}F NMR (471 MHz, DMSO-d_6), the signals of the fluorine F20 at various temperature

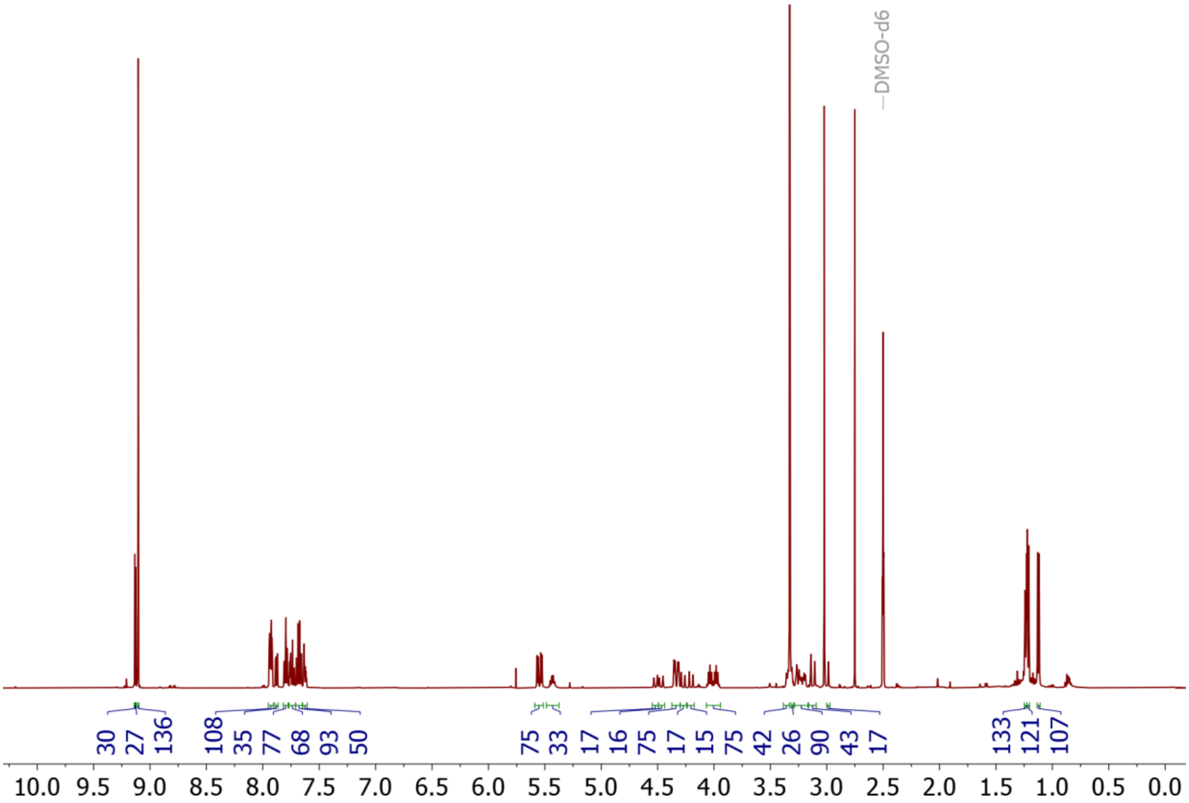

Figure S18. Compound **AM-12**, ^{1}H NMR (500 MHz, DMSO-d_6, 22°C)

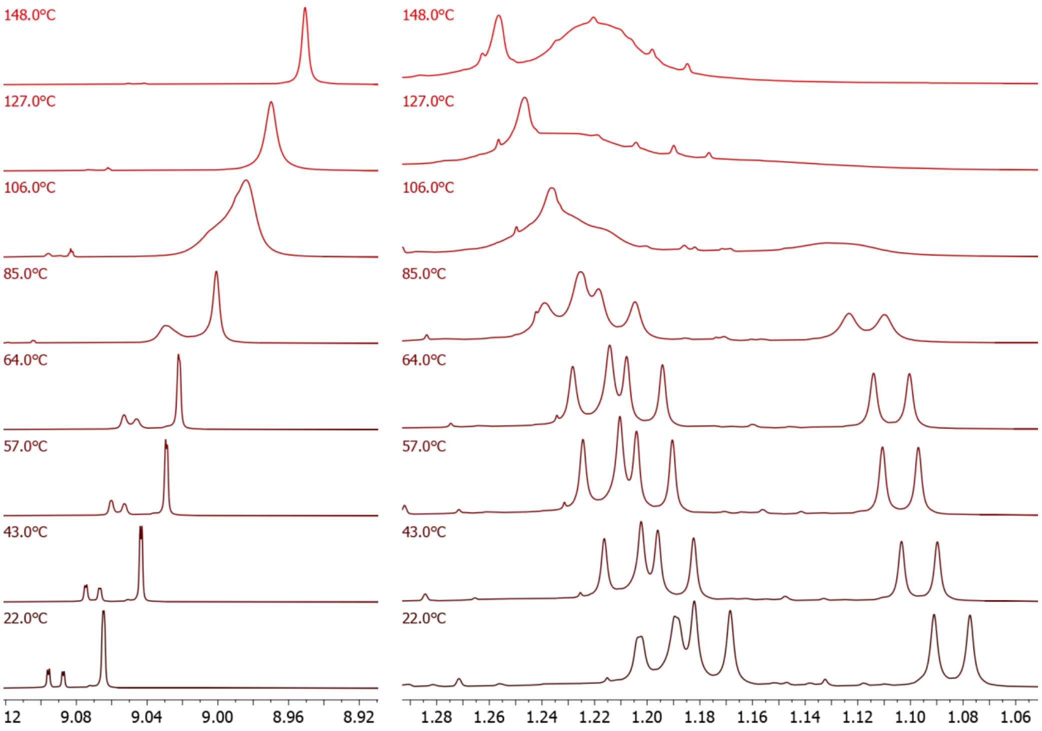

Figure S19. Compound **AM-12**, ^{1}H NMR (500 MHz, DMSO-d_6), H19 and the methyl signals at various temperature

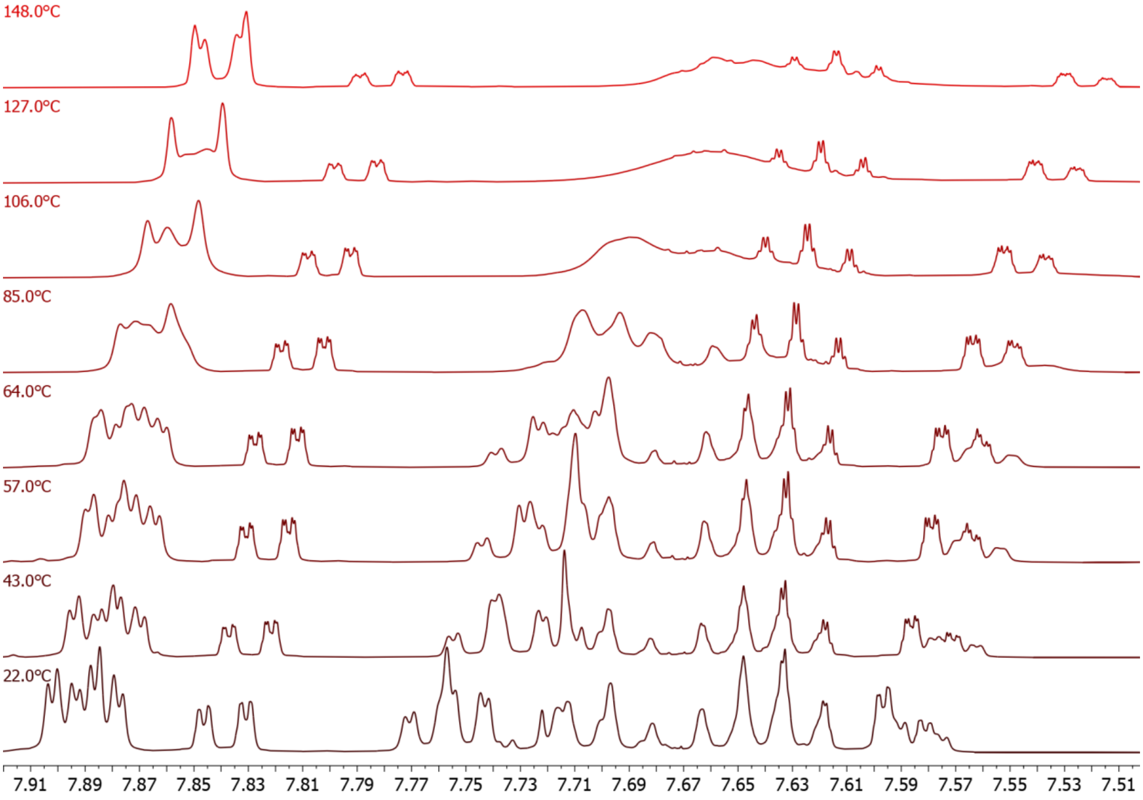

Figure S20. Compound **AM-12**, ^{1}H NMR (500 MHz, DMSO-d_6), H14, H15, and H6 at various temperature

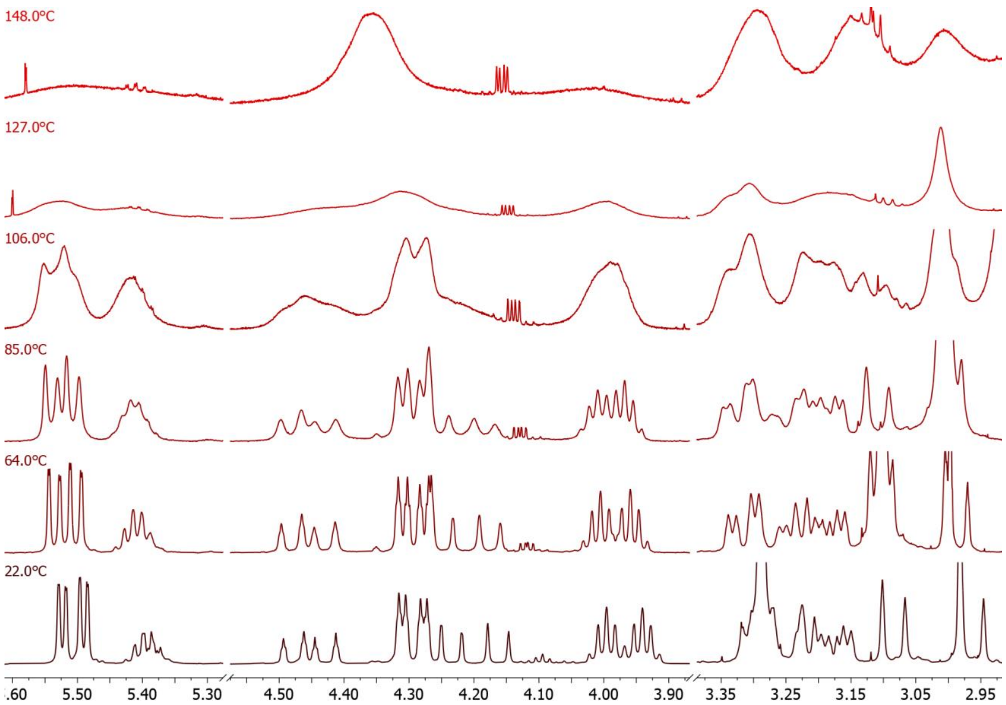

Figure S21. Compound **AM-12**, ^{1}H NMR (500 MHz, DMSO-d_6), H6a, H6b, H7, H9a and H9b at various temperature

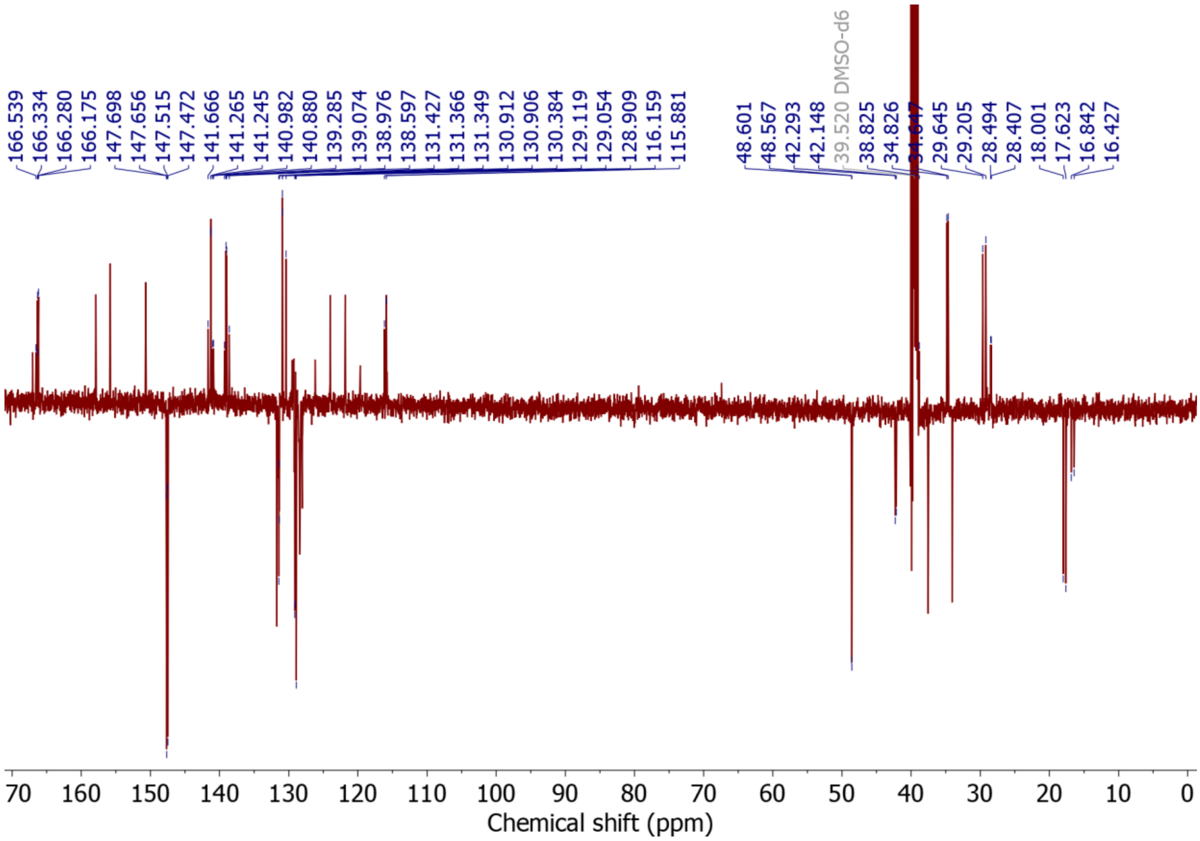

Figure S22. Compound **AM-12**, ^{13}C APT NMR (126 MHz, DMSO-d_6, 22°C)

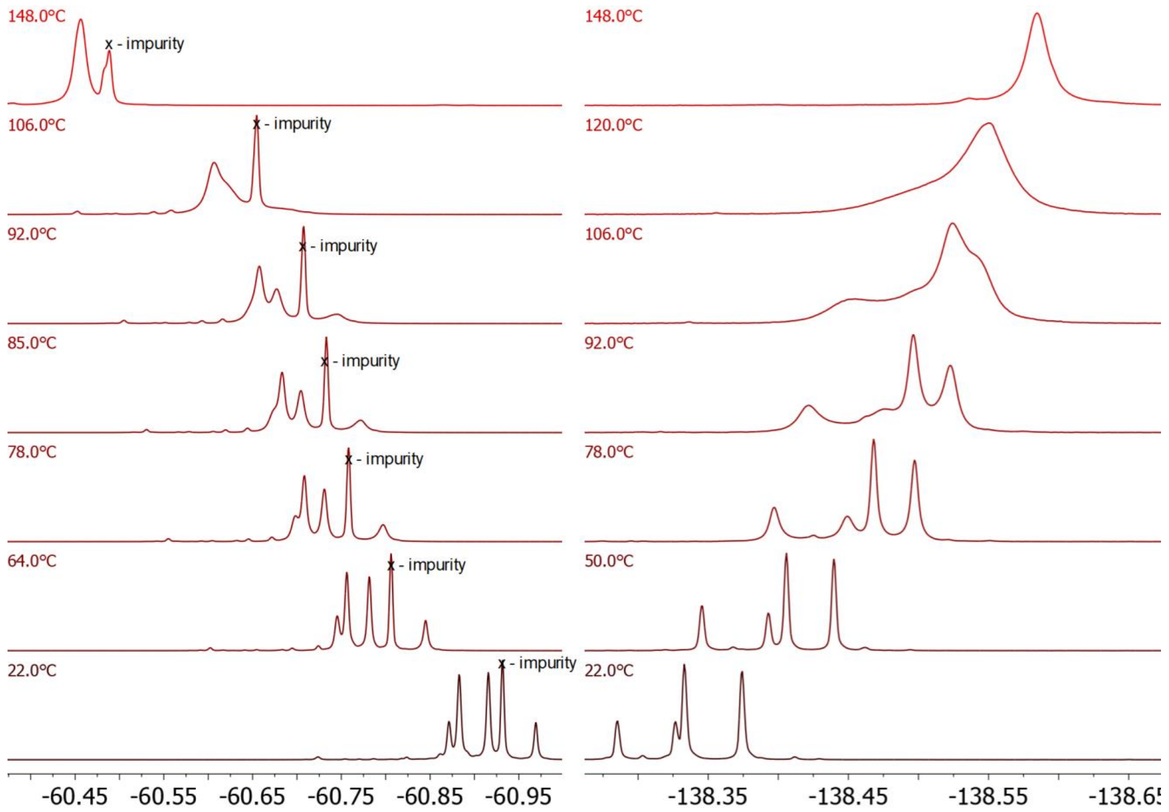

Figure S23. Compound **AM-12**, ^{19}F APT NMR (471 MHz, DMSO-d_6) at various temperature

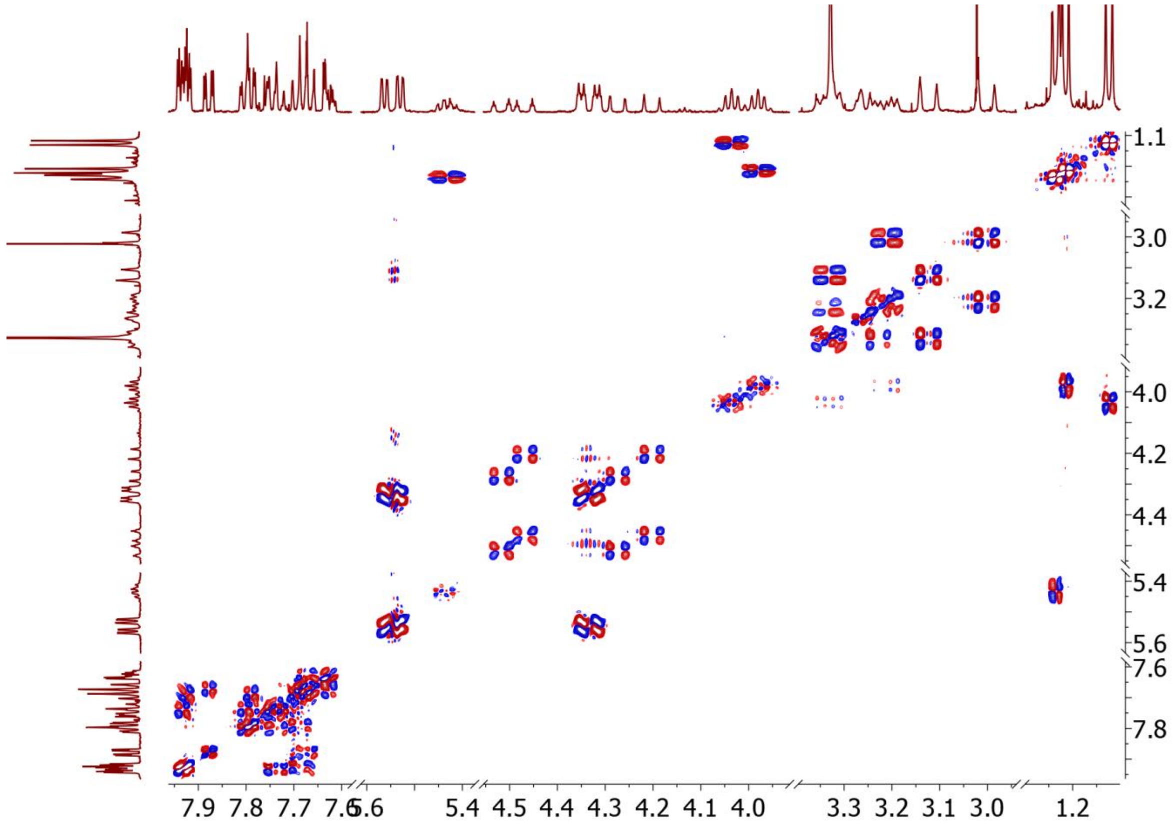

Figure S24. Compound **AM-12**, ^{1}H-^{1}H DQF COSY NMR (500 MHz, DMSO-d_6, 22°C)

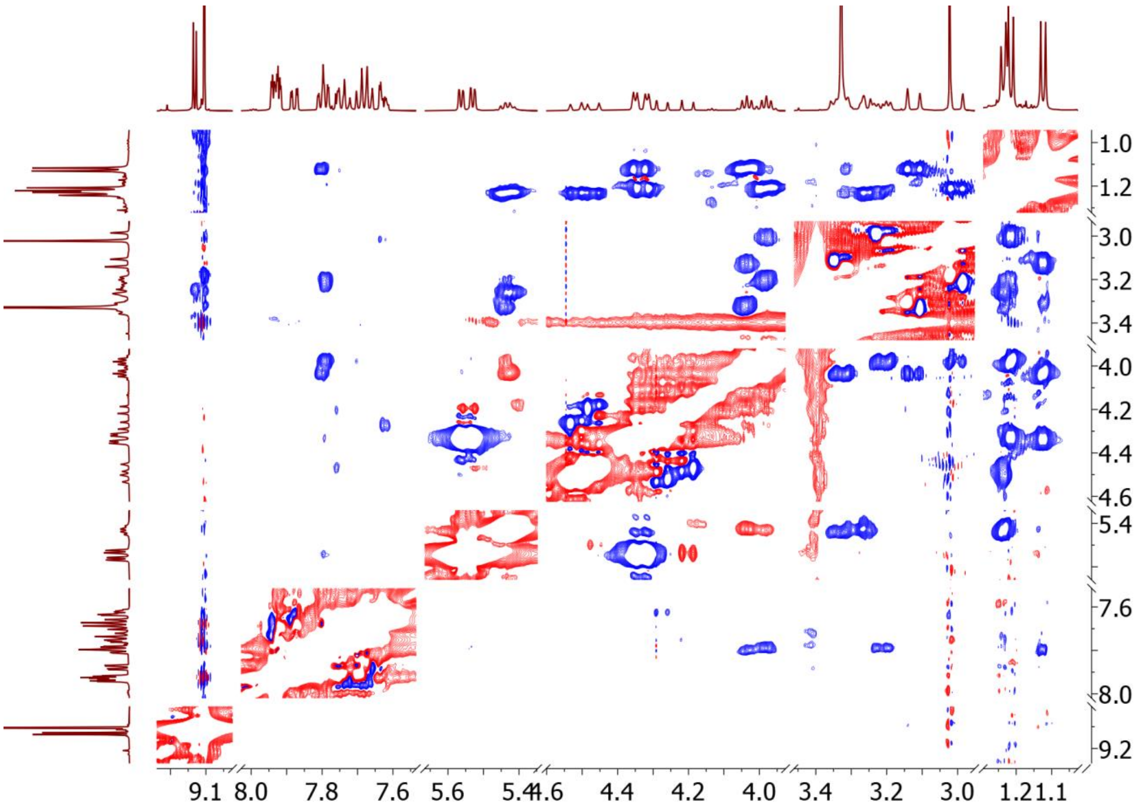

Figure S25. Compound **AM-12**, ^{1}H-^{1}H NOESY NMR (500 MHz, DMSO-d_6, 25°C, mixing time 350 ms)

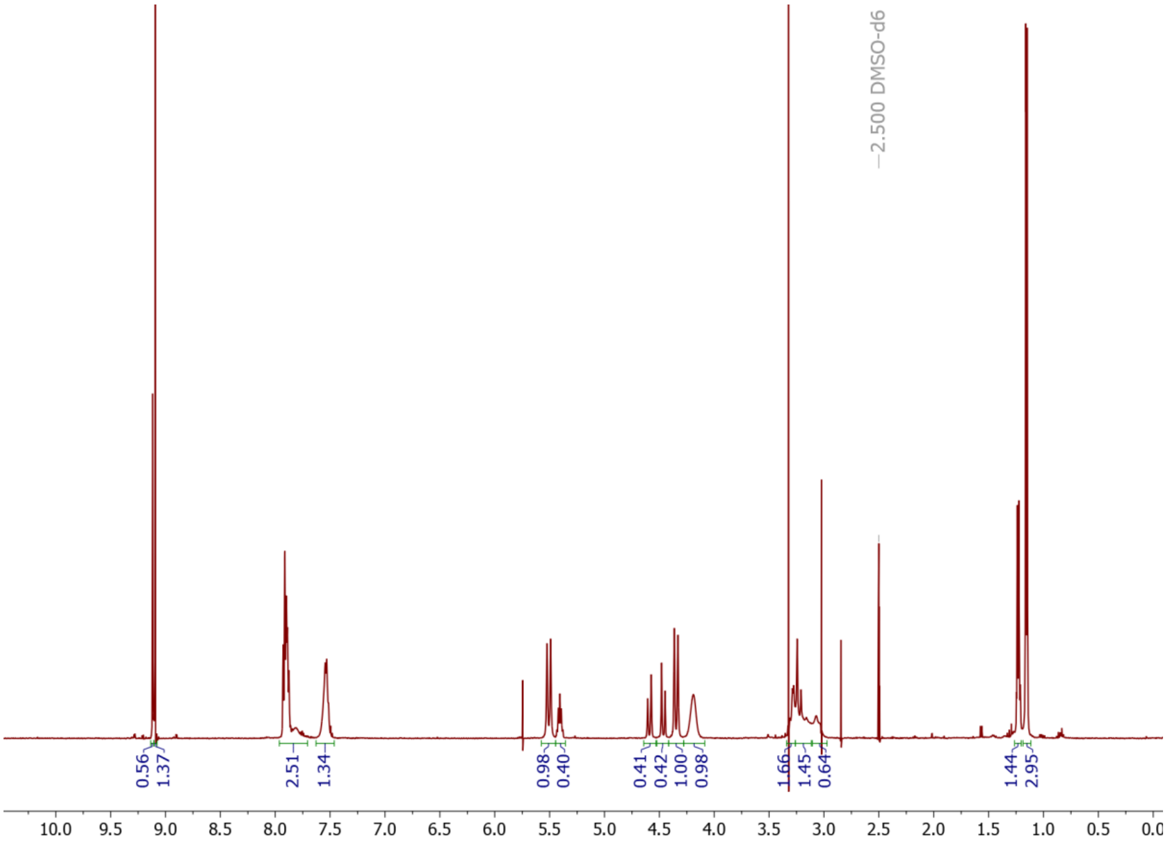

Figure S26. Compound **AM-15**, ^{1}H NMR (500 MHz, DMSO-d_6, 25°C)

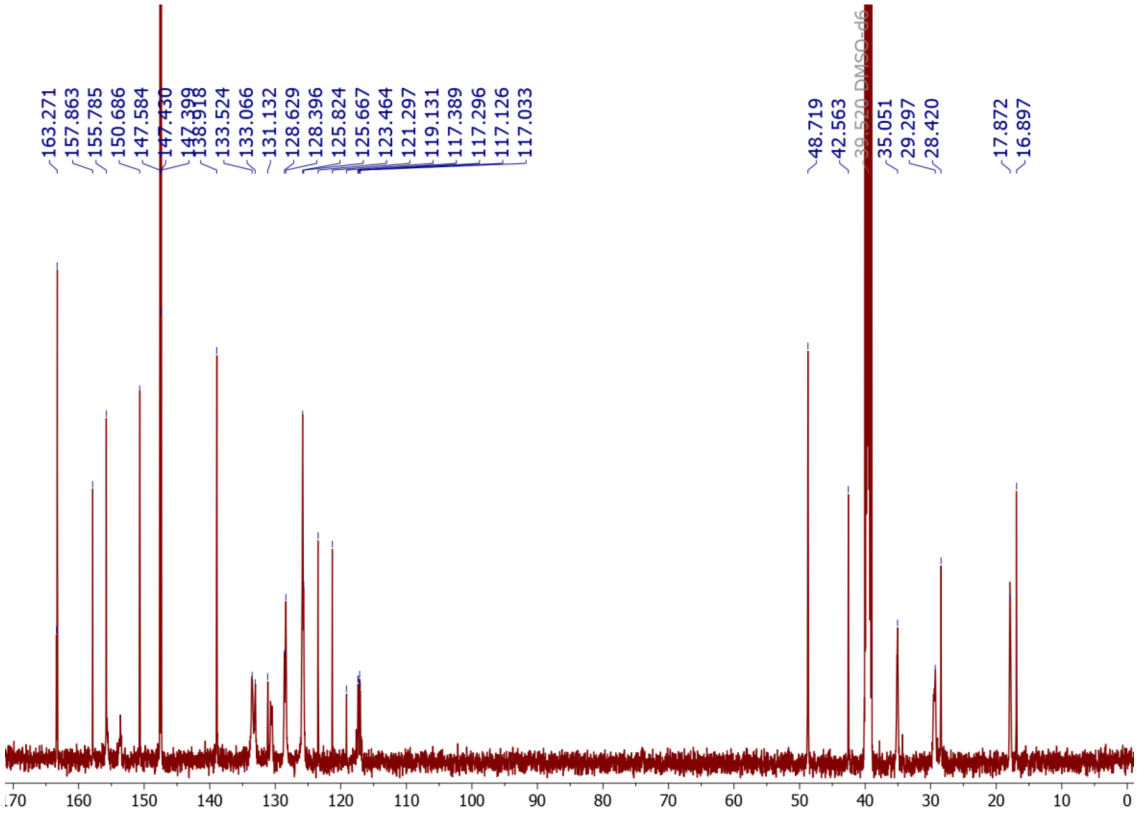

Figure S27. Compound **AM-15**, ^{13}C NMR (126 MHz, DMSO-d_6, 25°C)

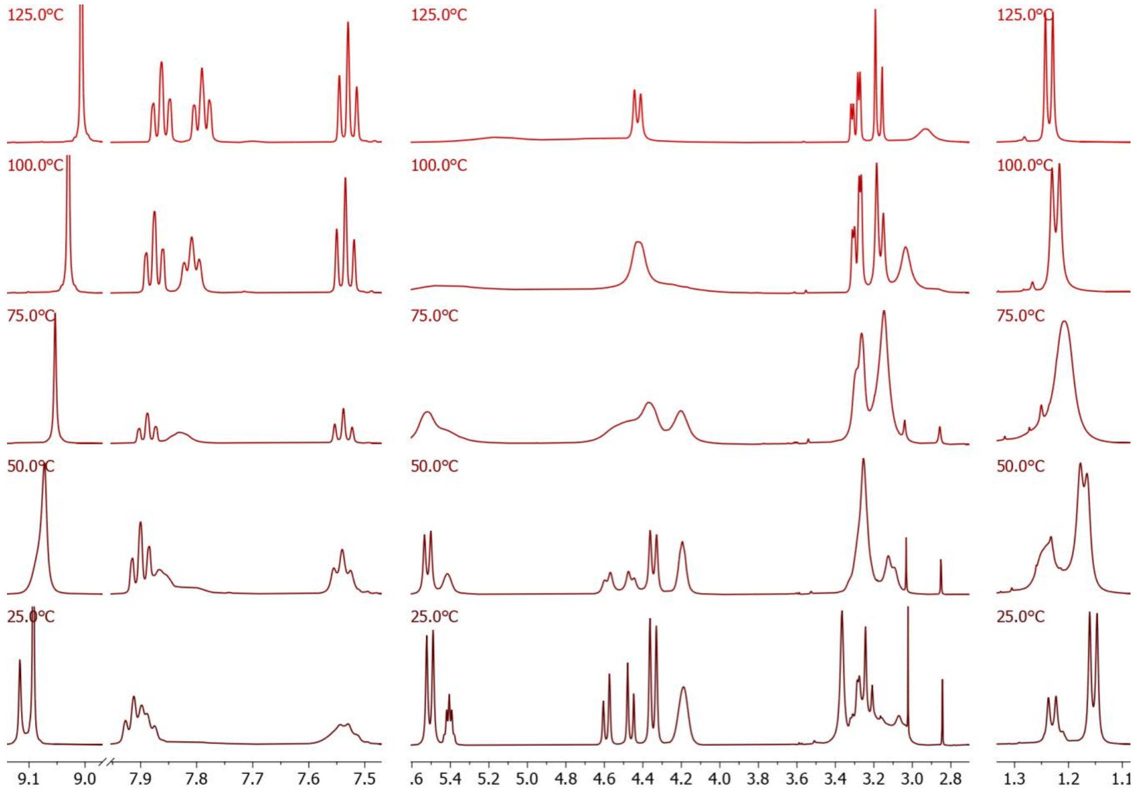

Figure S28. Compound **AM-15**, ^{1}H NMR (126 MHz, DMSO-d_6) at various temperature

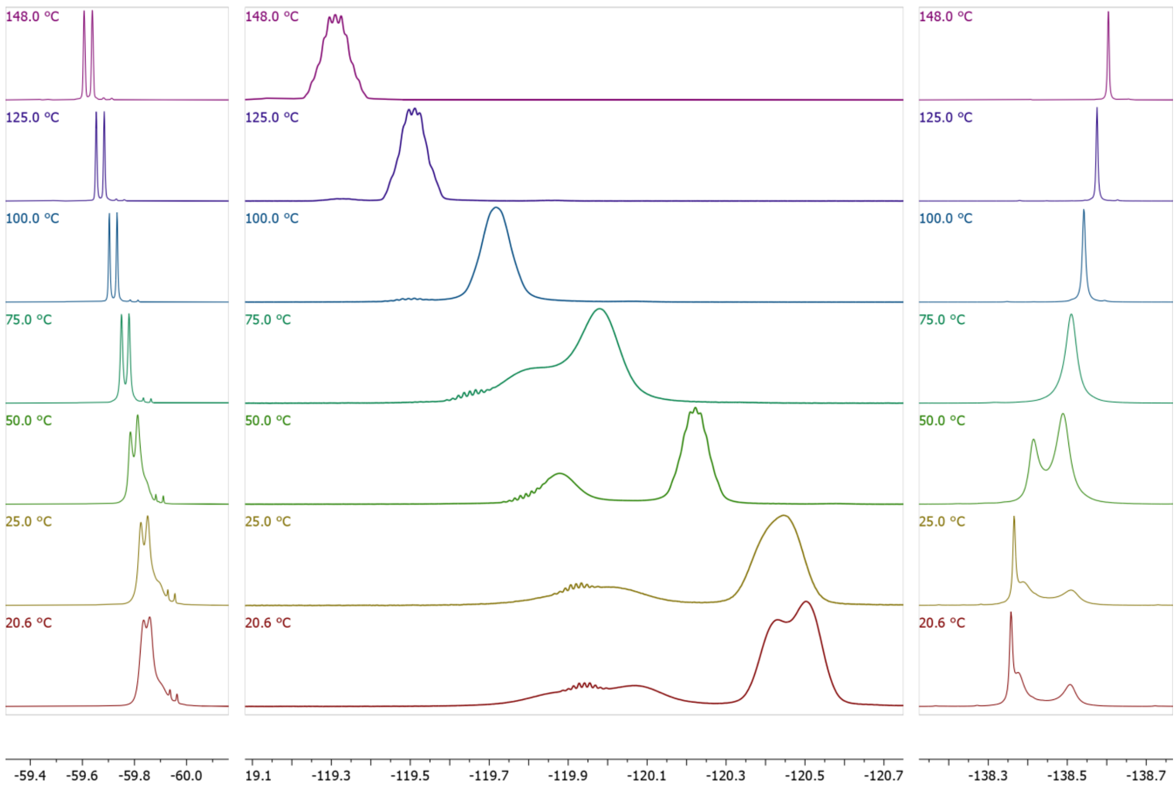

Figure S29. Compound **AM-15**, ^{19}F NMR (471 MHz, DMSO-d_6) at various temperature

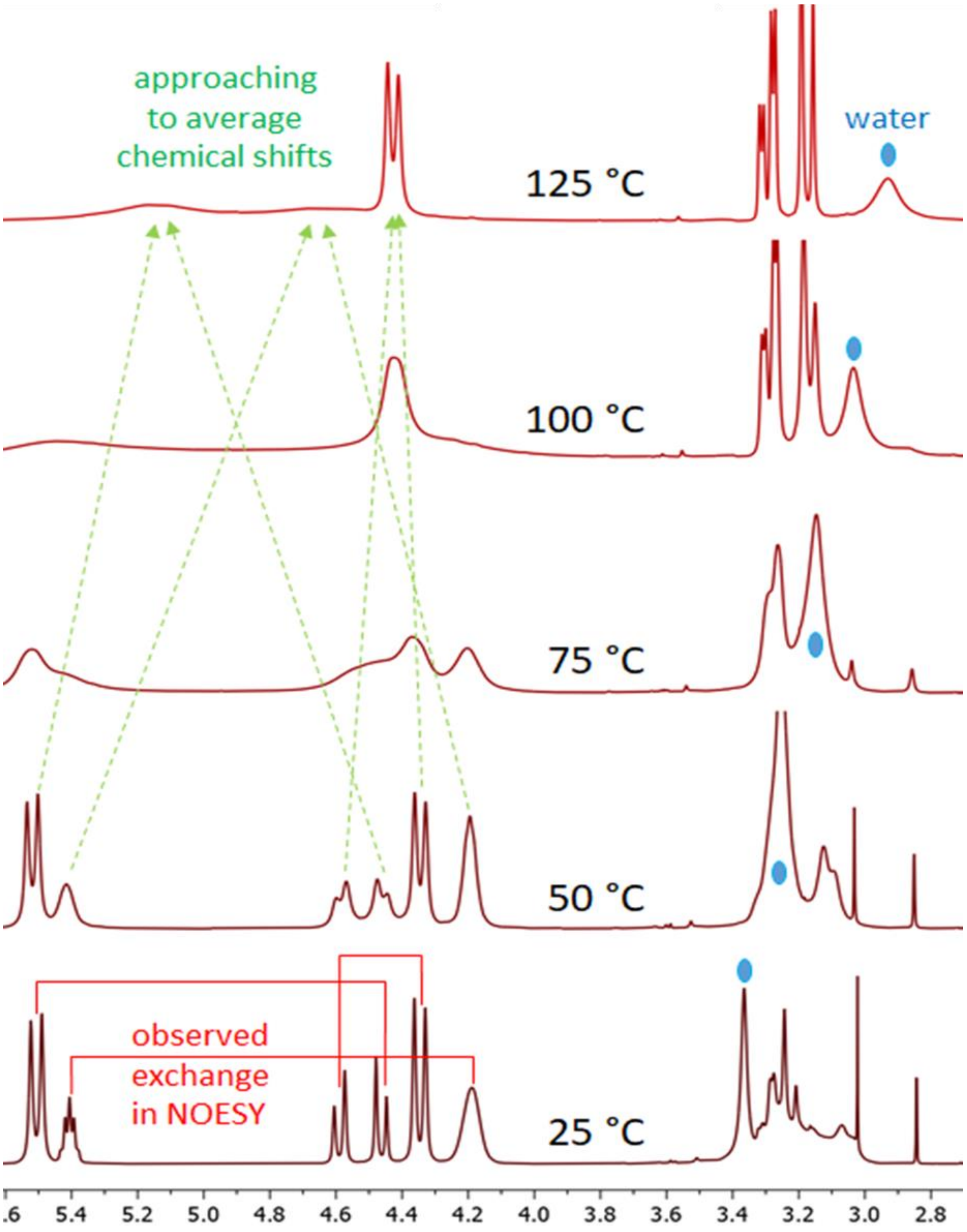

Figure S30. Compound **AM-15**, ^{1}H NMR (126 MHz, DMSO-d_6) at various temperature

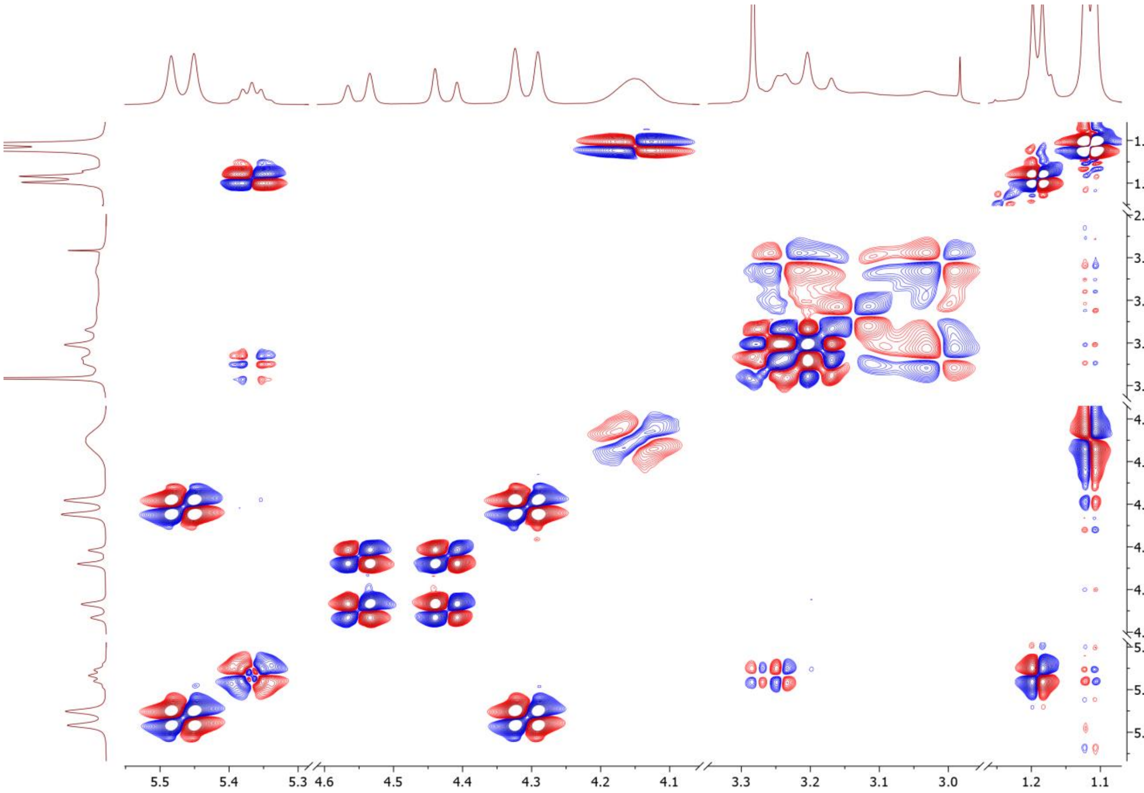

Figure S31. Compound **AM-15**, ^{1}H-^{1}H DQF COSY NMR (500 MHz, DMSO-d_6, 25°C)

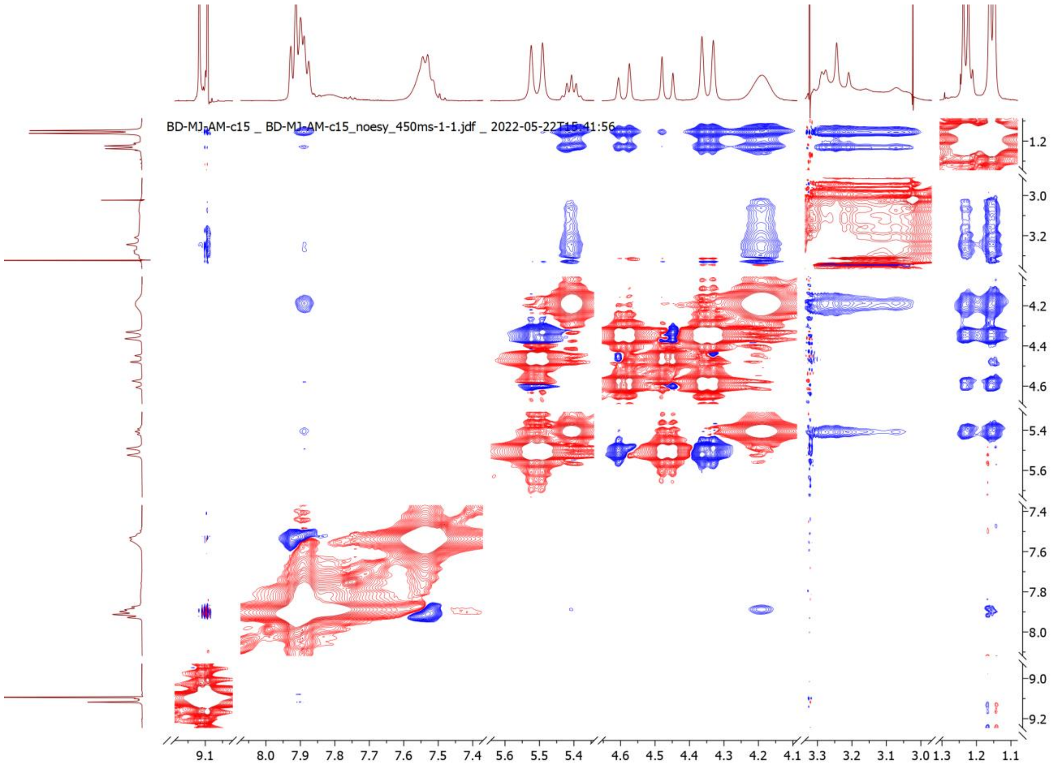

Figure S32. Compound **AM-15**, ^{1}H-^{1}H NOESY NMR (500 MHz, DMSO-d_6, 25°C, mixing time 450 ms)

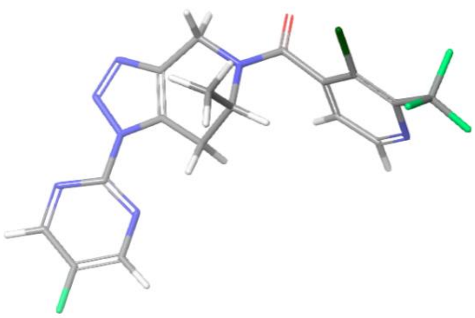

AM-10-Ab

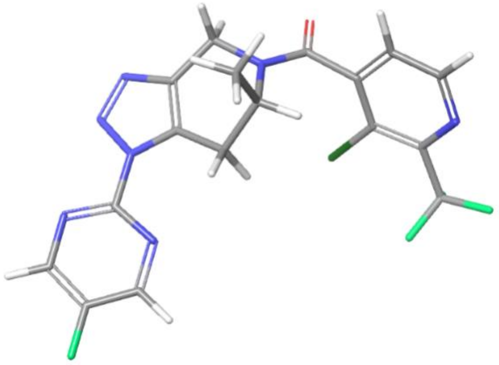

AM-10-Aa

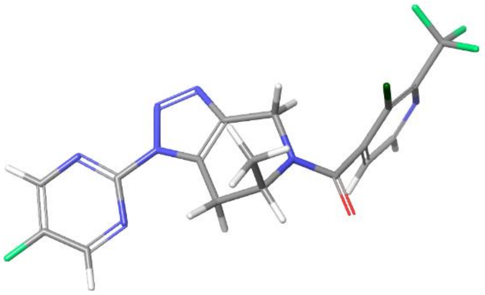

AM-10-Bb

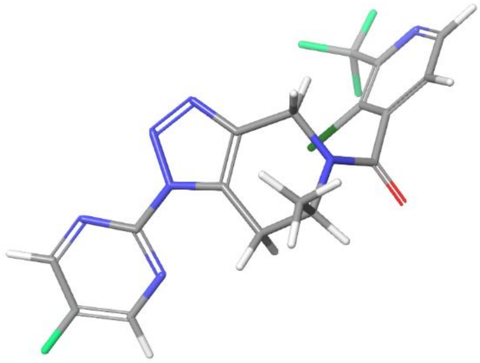

AM-10-Ba

Figure S33. Populated conformers of **AM-10** found by QM calculation.

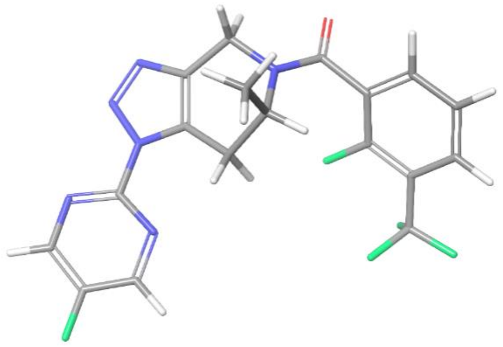

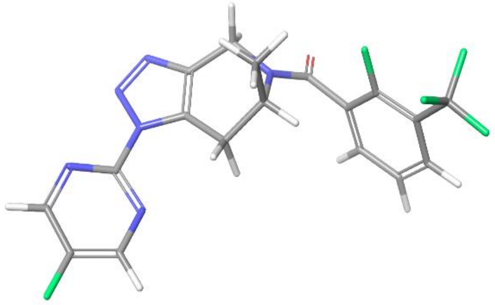

AM-15-Aa **AM-15-Ab**

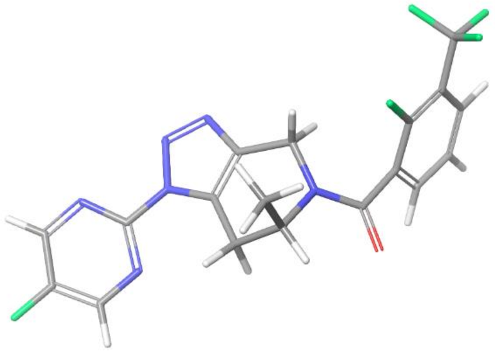

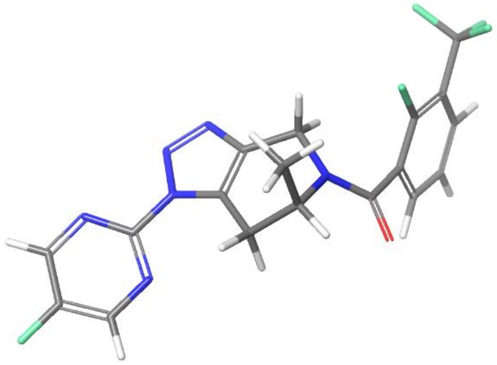

AM-15-Bb1 **AM-15-Bb2**

Figure S34. Populated conformers of **AM-15** found by QM calculation.

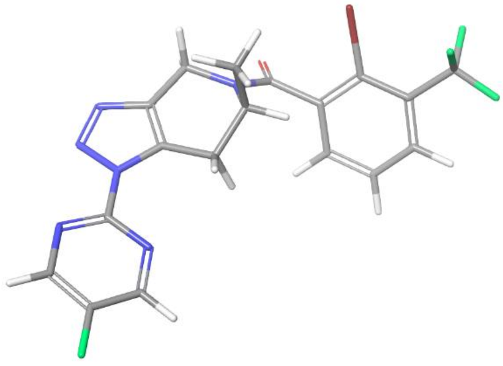

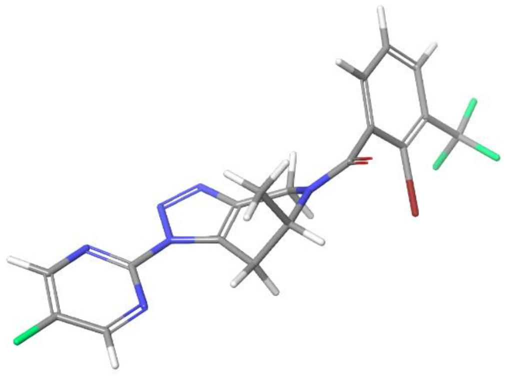

AM-12-Ab **AM-12-Aa**

Figure S35. Populated conformers of **AM-12** found by QM calculation.

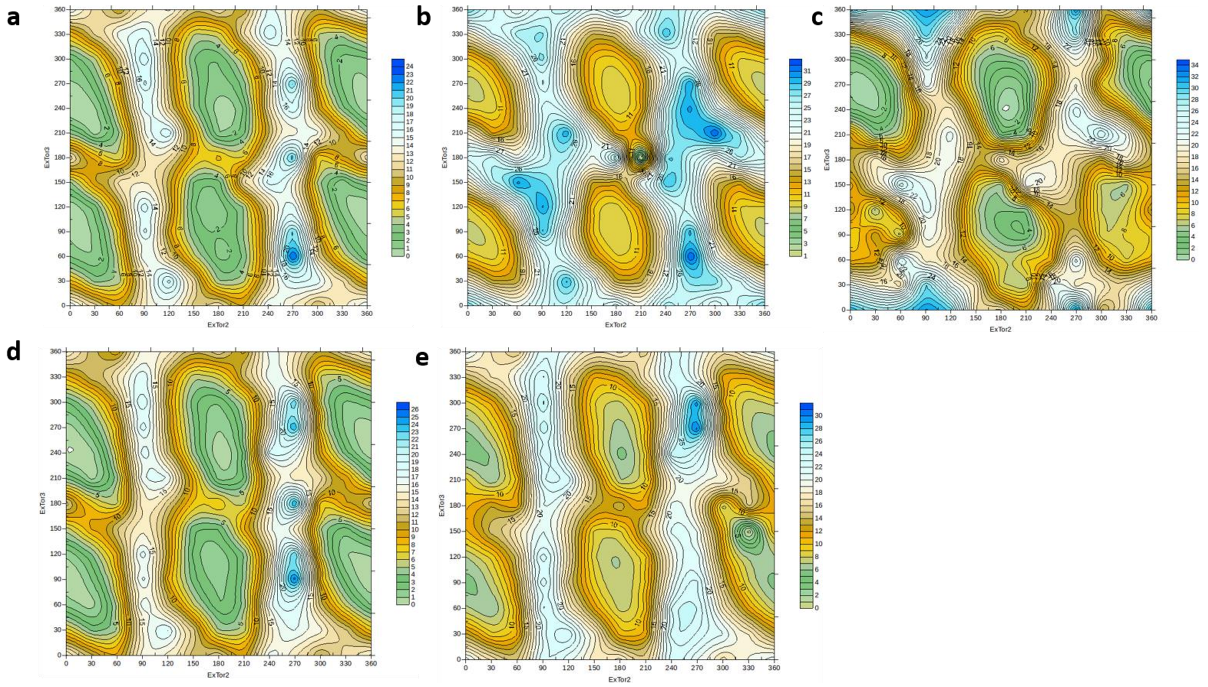

Figure S36. Relaxed scan free energy maps (energies are given in kcal/mole); a, AM-15; b, AM-10; c, AM-12; d, Et-JNJ-55308942; e, ^{i}Pr-JNJ-55308942.

Supplementary references

(s1) Han, J.; Liu, H.; Liu, C.; Jin, H.; Perlmutter, J. S.; Egan, T. M.; Tu, Z. Pharmacologic characterizations of a P2X7 receptor-specific radioligand, [11C]GSK1482160 for neuroinflammatory response. *Nucl. Med. Commun.* **2017**, *38* (5), 372-382.

(s2) Territo, P. R.; Meyer, J. A.; Peters, J. S.; Riley, A. A.; McCarthy, B. P.; Gao, M.; Wang, M.; Green, M. A.; Zheng, Q.-H.; Hutchins, G. D. Characterization of 11C-GSK1482160 for targeting the P2X7 receptor as a biomarker for neuroinflammation. *J. Nucl. Med.* **2017**, *58* (3), 458-466.

(s3) Michel; Chambers; Clay; Condreay; Walter; Chessell. Direct labelling of the human P2X7 receptor and identification of positive and negative cooperativity of binding. *Br. J. Pharmacol.* **2007**, *151* (1), 84 - 95.

(s4) Jin, H.; Han, J.; Resing, D.; Liu, H.; Yue, X.; Miller, R. L.; Schoch, K. M.; Miller, T. M.; Perlmutter, J. S.; Egan, T. M.; et al. Synthesis and in vitro characterization of a P2X7 radioligand [123I]TZ6019 and its response to neuroinflammation in a mouse model of Alzheimer disease. *Eur. J. Pharmacol.* **2018**, *820*, 8-17.

(s5) Beaino, W.; Janssen, B.; Kooijman, E.; Vos, R.; Schuit, R. C.; O'Brien-Brown, J.; Kassiou, M.; van het Hof, B.; Vugts, D. J.; de Vries, H. E.; et al. PET imaging of P2X7R in the experimental autoimmune encephalomyelitis model of multiple sclerosis using [11C]SMW139. *J. Neuroinflammation* **2020**, *17* (1), 300.

(s6) Donnelly-Roberts, D. L.; Namovic, M. T.; Surber, B.; Vaidyanathan, S. X.; Perez-Medrano, A.; Wang, Y.; Carroll, W. A.; Jarvis, M. F. [3H]A-804598 ([3H]2-cyano-1-[(1S)-1-phenylethyl]-3-quinolin-5-ylguanidine) is a novel, potent, and selective antagonist radioligand for P2X7 receptors. *Neuropharmacology* **2008**, *56* (1), 223-229.

(s7) Able, S. L.; Fish, R. L.; Bye, H.; Booth, L.; Logan, Y. R.; Nathaniel, C.; Hayter, P.; Katugampola, S. D. Receptor localization, native tissue binding and ex vivo occupancy for centrally penetrant P2X7 antagonists in the rat. *Br. J. Pharmacol.* **2010**, *162* (2), 405-414.

(s8) Lord, B.; Ameriks, M. K.; Wang, Q.; Fourgeaud, L.; Vliegen, M.; Verluyten, W.; Haspeslagh, P.; Carruthers, N. I.; Lovenberg, T. W.; Bonaventure, P.; et al. A novel radioligand for the ATP-gated ion channel P2X7: [3H] JNJ-54232334. *Eur. J. Pharmacol.* **2015**, *765*, 551-559.

(s9) Fu, Z.; Lin, Q.; Xu, Z.; Zhao, Y.; Cheng, Y.; Shi, D.; Fu, W.; Yang, T.; Shi, H.; Cheng, D. P2X7 receptor-specific radioligand 18F-FTTM for atherosclerotic plaque PET imaging. *Eur. J. Nucl. Med. Mol. Imaging* **2022**.

(s10) Kolb, H. C.; Barret, O.; Bhattacharya, A.; Chen, G.; Constantinescu, C.; Huang, C.; Letavic, M.; Tamagnan, G.; Xia, C. A.; Zhang, W.; et al. Preclinical evaluation and nonhuman primate receptor occupancy study of 18F-JNJ-64413739, a PET radioligand for P2X7 receptors. *J. Nucl. Med.* **2019**, *60* (8), 1154-1159.
